# Synthesis, Docking Profiles, and Biological Insights into Selenium-NHC Adducts and Benzimidazolium Salts with Antimicrobial Potential

**DOI:** 10.1021/acsomega.5c10880

**Published:** 2026-06-01

**Authors:** Boutheina Boualia, Abd Sandeli, Houssem Boulebd, Hüseyin Karci, Muhammed Dündar, Ilknur Özdemir, Nevin Gürbüz, Ahmet Koç, Ismail Özdemir

**Affiliations:** † The Molecular and Structural Environmental Chemistry Research Unit, Faculty of Exact Sciences, Brother’s Mentouri Constantine 1 University, Constantine 25000, Algeria; ‡ Pharmaceutical Science Research Center, Constantine 25000, Algeria; § Laboratory of Synthesis of Molecules with Biological Interest, Frères Mentouri Constantine 1 University, Constantine 25017, Algeria; ∥ Inönü University Drug Administration and Research Center, 44280 Malatya, Türkiye; ⊥ Inönü University, Faculty of Arts and Sciences, Department of Molecular Biology and Genetics, 44280 Malatya, Türkiye; # 37520 Inönü University, Faculty of Arts and Sciences, Department of Chemistry, 44280 Malatya, Türkiye; ∇ Inönü University Faculty of Medicine, Department of Medical Genetics, 44280 Malatya, Türkiye

## Abstract

In this research, we present the successful synthesis of benzimidazolium salts (**2a**–**e**) and their corresponding selenium-NHC adducts (**3a**–**e**), achieved with satisfactory yields ranging from 75% to 88%, and characterized using spectroscopic techniques, including NMR, FTIR, and mass spectrometry analysis. This work is the first to evaluate the antimicrobial activities and molecular docking studies of these novel compounds, shedding light on the limited enhancement provided by selenium incorporation and providing valuable insights into their enzyme inhibition mechanisms. The antimicrobial and antifungal activities of these compounds were evaluated against various bacterial and yeast strains using disk diffusion and minimum inhibitory concentration (MIC) methods. Benzimidazolium salts, particularly 2d and 2e, demonstrated superior antimicrobial efficacy against *Staphylococcus aureus* with inhibition zones of 26.73 mm and 18.10 mm and MIC values of 1.56 μg/mL, and against *Candida albicans* with inhibition zones of 18.10 mm and MIC values of 25 μg/mL and 12.5 μg/mL respectively, significantly outperforming the reference agents Ampicillin (15.33 mm inhibition zone; MIC: 25 μg/mL for *S. aureus*) and Caspofungin (14.30 mm inhibition zone; MIC: 25 μg/mL for *C. albicans*). Additionally, compounds 2d and 2e exhibited better activity against *Escherichia coli* with MIC values of 12.5 μg/mL compared to Ampicillin (25 μg/mL). Conversely, selenium-NHC compounds exhibited moderate to weak activity, with inhibition zones ranging from 8.35 to 11.93 mm and MIC values ranging from 100 to 800 μg/mL, and did not outperform the reference agents. To elucidate the potential mechanism of action, molecular docking studies were performed on compound 2d and its derivative 3d against three key bacterial enzymes—DNA gyrase, dihydrofolate reductase (DHFR), and tyrosyl-tRNA synthetase (TyrRS)—in addition to the fungal sterol 14-α-demethylase (CYP51) to assess possible antifungal interactions. The results revealed strong binding affinities for both bacterial and fungal enzymes. The compounds interact with crucial amino acids in the enzyme active sites, mirroring the interactions of native ligands. However, the presence of selenium in complex 3d did not enhance its inhibitory activity significantly compared to the salt **2d**. The findings highlight the potential of benzimidazolium salts, particularly **2d** and **2e**, as promising therapeutic agents for microbial infections, with selenium incorporation offering limited enhancement in activity.

## Graphical abstract

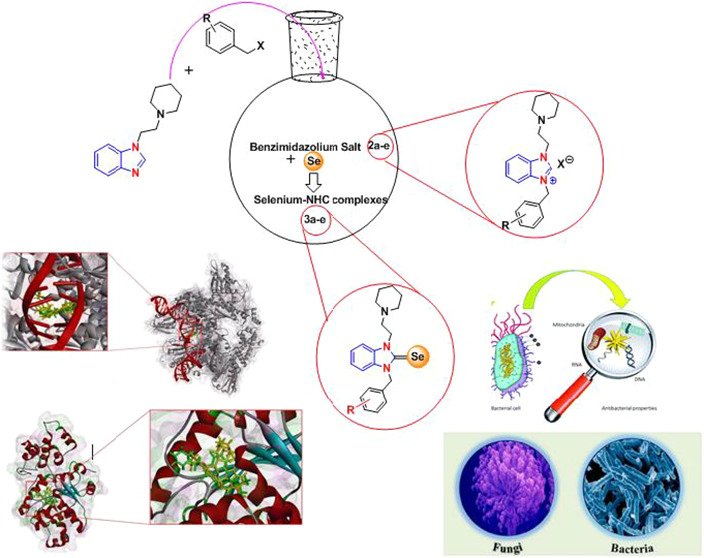

## 1 Introduction

As a metalloid, selenium (Se) exhibits chemical traits comparable to oxygen and sulfur. Selenium is found in trace amounts in food and beverages, where it plays a vital role in human health.^1^ While selenium is nontoxic to humans at low concentrations,^2–5^ its higher concentrations can exhibit toxic effects,^6–8^ underscoring the importance of proper dosage in supplementation. Selenium’s role in medicinal chemistry has gained increasing attention due to its selectivity, biocompatibility, and efficacy.^9^ Selenium-containing compounds have shown significant promise in various therapeutic areas, including cancer therapy, antimicrobial, antiviral, antioxidant, anti-inflammatory, and antineurodegenerative activities.^10–14^

However, delivering selenium to target sites effectively remains a challenge. Its increasing relevance in medicinal chemistry stems from reports highlighting its selectivity, biocompatibility, and high efficacy. Selenium’s application in medicinal chemistry is beneficial for treating various illnesses due to its compatibility with biological systems, in contrast to other elements in the periodic table. For instance, selenium supplements are used to address deficiencies, while selenium sulfide is included in shampoos for dandruff treatment.^15,16^ Recent interest in selenium-containing derivatives for drug discovery has increased, largely due to their biocompatibility and lower risk of side effects.^17–20^ However, one of the most significant challenges lies in effectively delivering selenium to target sites within biological systems. Efforts to address this challenge are ongoing, recognizing the crucial role selenium plays. Furthermore, research indicates that metal-based antimicrobial agents show promise in addressing bacterial resistance and could potentially serve as future antimicrobial drugs.^21,22^

Despite their superior antimicrobial activities compared to organic compounds, coordination compounds often lose their effectiveness before reaching the target site because of the rapid release of metal ions.^23^ This issue has been addressed by using metal complexes of *N*-heterocyclic carbenes,^24^ as NHCs release metal ions more slowly than other coordination compounds, thereby prolonging their biological effects.^23^

Metal-based compounds, particularly those containing selenium, have been explored for their antimicrobial properties, offering a potential solution to the growing issue of bacterial resistance. However, many coordination compounds lose their effectiveness due to the rapid release of metal ions before reaching the target site. This limitation can be overcome by utilizing metal complexes with *N*-heterocyclic carbenes (NHCs), which have been shown to release metal ions more gradually, prolonging their biological effects and enhancing their therapeutic potential. NHCs have become widely recognized as versatile ligands in organometallic chemistry^25–27^ and are increasingly applied in medicinal chemistry,^25,28,29^ particularly in the design of metal complexes with promising antibacterial^30–32^ and anticancer activities.^33–35^

While recent research has increasingly focused on the antibacterial and anticancer applications of silver­(I)- and Au­(I)-NHC complexes, various selenium-based compounds have also been evaluated and found to demonstrate promising antimicrobial activity.^36–39^ In this context, and as part of our ongoing research selenium-based NHC complexes have emerged as attractive candidates for drug development, as well as the challenges of improving the efficacy and targeted delivery of antimicrobial agents. We hypothesized that the incorporation of selenium into NHC-based frameworks could enhance their antimicrobial activity while maintaining biocompatibility. Our research builds on previous studies by synthesizing novel selenium-NHC adducts and their related benzimidazolium salts to exploring their antimicrobial potential and antifungal activities. In the present study, we designed and synthesized five new Se-NHC compounds and their corresponding benzimidazolium salts, aiming to evaluate their biological activities and investigate their mechanisms of action. We also performed molecular docking studies to further understand the interactions of these compounds with key bacterial enzymes, providing insights into their potential as therapeutic agents.

## 2 Results and Discussion

### 2.1 Chemistry

Azolium salts (**2a**–**e**) and their corresponding Se-NHC complexes (**3a**–**e**) were synthesized following previously reported methods.^40–42^ Studies indicate that the synthesis of Se-NHC complexes has been achieved through multiple approaches, some involving conventional steps.

#### 2.1.1 Synthesis of Benzimidazolium Salts **2a–e**

Benzimidazolium chloride salts **2a–e** were obtained through a sequential *N*-alkylation strategy (Scheme 1). In the first step, 1*H*-benzo­[*d*]­imidazole was alkylated with 1-(2-chloroethyl)­piperidine hydrochloride to furnish intermediate 1. Subsequent quaternization of intermediate 1 with the appropriate benzyl chloride derivatives in toluene at 80 °C afforded the targeted benzimidazolium salts **2a–e.**

**Scheme 1:**
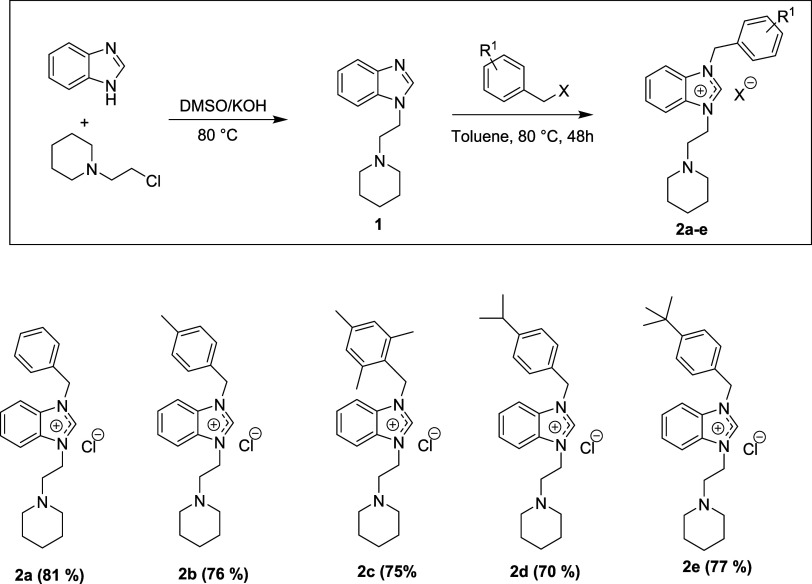
Synthetic Route for the Preparation of Benzimidazolium Salts **2a**–**e**

All derivatives were isolated in good to satisfactory yields. Their structures were confirmed by spectroscopic analysis, and the obtained data were consistent with those reported for structurally related benzimidazolium salts in the literature.^43,44^ The compounds **2a**–**e** exhibited good stability under ambient conditions and were stored without observable decomposition. The relevant physical characteristics and selected spectroscopic data are summarized in Table 1

**Table 1:** Physical and Spectroscopic Data for Benzimidazolium Salts **2a–e.**

code	chemical formula	molecular weight (g/mol)	melting point °C	^1^H NMR (C2–H) ppm	^13^C NMR (C2) ppm	IR ν(CN)cm^–1^
**2a**	C_21_H_26_ClN_3_	355.91	180–182	11.41	143.20	1554
**2b**	C_22_H_28_ClN_3_	369.94	172	11.41	144.09	1558
**2c**	C_24_H_32_ClN_3_	397.99	232	10.41	143.49	1561
**2d**	C_24_H_32_ClN_3_	397.99	156–158	11.28	144.01	1563
**2e**	C_25_H_34_ClN_3_	412.02	258–260	11.25	143.96	1561

#### 2.1.2 Synthesis of Selenium *N*-Heterocyclic Carbene Complexes **3a–e**

Selenium–NHC complexes **3a–e** were generated from the corresponding benzimidazolium chloride precursors by reaction with elemental selenium in the presence of potassium carbonate (Scheme 2). The base-promoted in situ formation of the carbene followed by trapping with selenium was carried out in methanol at 80 °C, affording the desired selenium adducts as black crystalline solids.

**Scheme 2:**
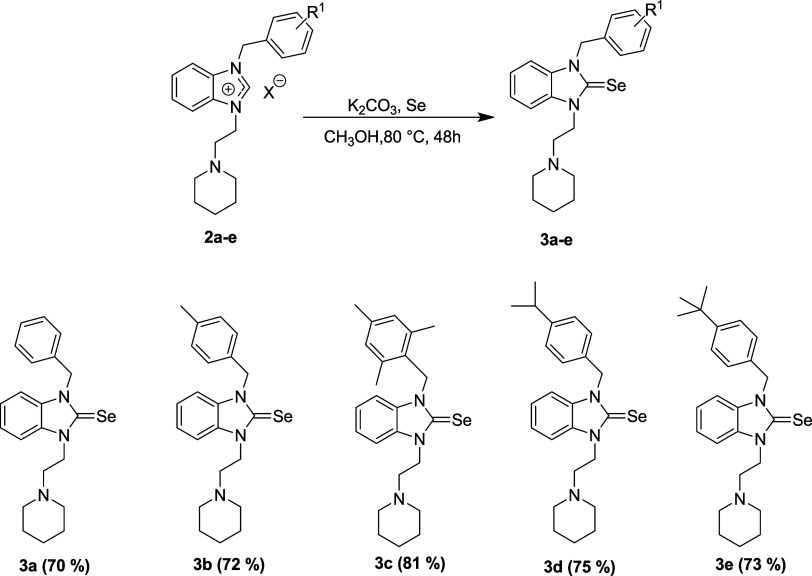
Synthetic Route for the Preparation of Selenium-NHC Compounds **3a–e.**

The isolated complexes were obtained in satisfactory yields illustrated in Scheme 2. Their spectroscopic characterization confirmed their structures, and the observed data are in agreement with values reported for related selenium–NHC systems in the literature^39,44–46^ These complexes showed stability toward air and moisture under normal laboratory conditions. Their main physical properties and selected spectroscopic parameters are compiled in Table 2. In the present study, this established synthetic approach was applied to prepare a new series of derivatives for further structural and physicochemical investigation.

**Table 2:** Physical and Spectroscopic Data for Se-NHC Compounds **3a**–**e**

code	chemical formula	molecular weight (g/mol)	melting point °C	^13^C NMR (C2) ppm	IR ν(CN) cm^–1^
**3a**	C_21_H_25_N_3_Se	398.41	126	167.2	1154
**3b**	C_22_H_27_N_3_Se	412.44	118	167.0	1192
**3c**	C_24_H_31_N_3_Se	440.49	164	166.4	1203
**3d**	C_24_H_31_N_3_Se	440.49	80	167.1	1198
**3e**	C_25_H_33_N_3_Se	454.52	130–132	166.4	1198

A successful synthesis of the benzimidazolium salts (**2a**–**e**) and their respective Se-NHC compounds was initially indicated by their solubility, physical states, and a noticeable difference in melting points between the salts and the Se- ^44–46^ NHC compounds. The successful synthesis and isolation of both benzimidazolium salts and selenium-NHC compounds, with good to excellent yields, is demonstrated by these results. The compounds’ melting points and physical appearances further confirm their purity and identity. The variations in melting points also suggest differences in the molecular packing and intermolecular interactions within the solid state of each compound. The observed melting points for the synthesized salts (**2a**–**e**) ranged from 156 to 258 °C, in contrast to the melting points of their respective Se-NHC adducts (**3a**–**e**), which ranged from 80 to 164 °C. This variation highlights the distinction between the inorganic and organic nature of the compounds. The compounds yielded percentages between 69% and 85% and showed stability when exposed to moisture and air.

At first, the selenium-NHC compounds appeared as a sticky brown substance. However, recrystallization produced a thick light yellow liquid that gradually turned colorless with additional recrystallization. The benzimidazolium salts, on the other hand, appeared as white solids in the reaction medium. Their appearance was influenced by the type of alkyl chain substituted on the nitrogen atoms of the benzimidazole group. Furthermore, both the benzimidazolium salts and their selenium-NHC complexes (**3a**–**e**) were found to be soluble in nonpolar solvents such as chloroform and dichloromethane.

Various techniques were employed to characterize all benzimidazolium salts and their respective selenium-N-heterocyclic carbene compounds. Changes before and after the incorporation of selenium into the carbene carbon were assessed using FT-IR spectra. Distinct changes observed in the spectral features of the compounds before and after selenium bonding offer preliminary evidence of successful metal incorporation into the organic framework. Comparing the spectral features revealed prominent changes in the 1100–1600 cm^–1^ region when analyzing the salts versus the Se-NHC adducts. FT-IR data indicated that all benzimidazolium salts (**2a**–**e**) exhibit a characteristic ν­(CN) band at 1554, 1558, 1561, 1563, and 1561 cm^–1^ respectively

^1^H and ^13^C NMR spectra for the salts (**2a**–**e**) and their corresponding selenium-NHC adducts (**3a**–**e**) were recorded in deuterated chloroform, based on their solubility. in the ^1^H NMR spectra, the salts (**2a**–**e**) exhibited a notable peak that appeared as a singlet at δ = 11.25–11.45 ppm for the acidic proton NC **H** N, which confirmed the salt formation. Upon coordination with selenium, the most notable change is the disappearance of the NC **H** N peak, indicating the replacement of the acidic proton by selenium. Aromatic protons resonate in the δ 7.2–8.0 ppm range, with slight upfield shifts in compounds containing electron-donating groups, such as methyl substituents. The N–CH_2_–Ph methylene protons consistently appear as singlets at δ 5.79–5.88 ppm, while the broad multiplets and triplets in the δ 4.7–1.4 ppm range correspond to methylene groups in the piperidine ring. Distinct alkyl substituents, such as CH_3_ or CH_3_ groups, show characteristic peaks in the aliphatic region, e.g., δ 2.31 ppm for CH_3_ in 2b or δ 1.27 ppm for 3CH_3_ in **2e**. In the selenium adducts, aromatic protons shift slightly upfield, resonating between δ 6.4–7.3 ppm, reflecting increased electron density in the aromatic system due to carbene coordination. The N–CH_2_–Ph protons remain nearly unchanged at δ 5.67–5.73 ppm, and the piperidine methylene signals show no significant shifts, suggesting that the piperidine group is not directly affected by selenium coordination. Overall, these spectral changes provide clear evidence for the formation of selenium-NHC adducts and highlight the influence of substituents on the electronic environment of the compounds.

In the ^13^C NMR data for benzimidazolium salts (**2a**–**e**) the carbon N **C** HN in the salts resonates at δ 143.2–144.1 ppm. The aromatic carbons appear in the δ 125.3–132.0 ppm range, with minor shifts depending on substituents. The aliphatic region reveals distinct signals: the Ph-CH_2_–Ph carbon appears at δ 55.7–56.8 ppm, CH_2_ groups of the piperidine ring resonate between δ 44.0–54.5 ppm, and alkyl substituents, such as CH_3_ in 2b and isopropyl or t-butyl groups in **2d** and **2e**, show characteristic peaks at δ 21.1–34.6 ppm. Upon selenium coordination, a key feature is the appearance of the carbene carbon bound to selenium (**C** =Se), which resonates prominently at δ 166.4–167.3 ppm, replacing the original NCHN signal. Aromatic carbons show slight downfield shifts, typically between δ 123.0–135.5 ppm, indicating electronic redistribution. The Ph–CH_2_–Ph and piperidine CH_2_ carbons remain nearly unchanged, resonating at δ 55.0–56.2 ppm and δ 44.6–50.2 ppm, respectively, suggesting these groups are not directly involved in selenium binding. Substituent-specific peaks, such as methyl, isopropyl, and t-butyl groups, persist in their respective aliphatic regions with minimal shifts, further supporting localized electronic effects. These changes in ^13^C NMR spectra provide strong evidence for successful selenium-NHC adduct formation and the preservation of the structural integrity of peripheral groups.

The mass spectroscopic results for benzimidazolium salts **2a**–**e** show that all calculated [M – Cl]^+^ values exactly match the found [M – Cl]^+^ values, indicating precise mass determination and confirming the molecular weights of the compounds. For example, for compound **2a**, the calculated [M – Cl]^+^ value is 320.2101, which matches exactly with the found value of 320.2101. Similarly, for compound **2e**, the calculated and found [M – Cl]^+^ values are both 376.2672.

However, for selenium NHC Complexes **3a**–**e**, the found [M + H]^+^ values are very close to the calculated [M + H]^+^ values, with slight deviations for some compounds. For instance, for compound **3b**, the calculated [M + H]^+^ value is 413.1370, and the found value is 413.3191. For compound **3c**, the calculated [M + H]^+^ value is 441.1683, and the found value is 442.1699. These minor discrepancies are within acceptable limits and confirm the molecular weights of the compounds, supporting the proposed structures. Overall, the HRMS (ESI) results provide strong evidence for the accurate determination of the molecular weights of the benzimidazolium salts and selenium NHC complexes, confirming their proposed structure.

### 2.2 Biological Evaluation

The primary aim of this study was to assess the antimicrobial and antifungal potential of the synthesized benzimidazolium salts and Se–NHC complexes, biological evaluation was focused on microbial inhibition rather than enzyme inhibition. Future studies will include in vitro enzymatic assays to experimentally confirm target engagement and validate the computational predictions, which will provide a more comprehensive mechanistic understanding of these compounds. Antimicrobial and antifungal activities of the synthesized benzimidazolium salts **2a**–**e** and their selenium-NHC compounds **3a**–**e** were tested against both bacterial and yeast strains. The control for yeast infections was caspofungin, and for bacterial infections, it was Ampicillin. The inhibition zone values for all compounds and reference antimicrobial agents are presented in Table 3, while the minimum inhibitory concentration (MIC) results (μg/mL) are shown in Table 4.

**Table 3:** Antifungal and Antibacterial Inhibition Zone Values^c^

	anti-fungal		anti-bacterial		
compounds	*Candida albicans* ^a^	*Candida glabrata* ^a^	*Escherichia coli* ^a^	*Pseudomonas aeruginosa* ^a^	*Staphylococcus aureus* ^a^
**2a**	8.20 ± 0.51	8.30 ± 0.39	12.13 ± 0.12	NA	21.27 ± 0.92
**2b**	8.55 ± 0.32	9.10 ± 0.14	14.53 ± 0.45	7.93 ± 0.34	23.33 ± 0.94
**2c**	10.20 ± 0.49	9.00 ± 0.24	16.80 ± 0.24	10.80 ± 0.22	23.67 ± 0.47
**2d**	16.73 ± 0.17	12.40 ± 0.57	19.00 ± 0.45	13.93 ± 0.19	26.73 ± 0.38
**2e**	18.10 ± 0.14	11.97 ± 0.21	18.10 ± 0.14	11.97 ± 0.05	26.33 ± 0.62
**3a**	8.85 ± 0.12	9.00 ± 0.49	9.07 ± 0.12	NA	11.90 ± 0.16
**3b**	8.35 ± 0.46	NA	11.93 ± 0.17	NA	10.50 ± 0.45
**3c**	8.40 ± 0.42	9.00 ± 0.00	NA	NA	NA
**3d**	9.70 ± 0.14	8.83 ± 0.31	9.80 ± 0.22	NA	9.03 ± 0.05
**3e**	9.10 ± 0.37	8.60 ± 0.28	8.93 ± 0.17	NA	8.83 ± 0.12
**Ampicillin** ^b^			14.27 ± 0.61	12.43 ± 0.42	15.33 ± 0.21
**Caspofungin** ^b^	14.30 ± 0.15	20.57 ± 0.61			

a Tested microorganism.

b Reference drugs.

c NA: Not Active (no inhibition zone).

**Table 4:** Antifungal and Antibakterial MIC Values (μg/mL)^c^

	anti-fungal		anti-bacterial		
Compounds	*C. albicans* ^a^	*C. glabrata* ^a^	*E. coli* ^a^	*P. aeruginosa* ^a^	*S. aureus* ^a^
**2a**	400	400	100	NA	6,25
**2b**	400	400	25	400	3,12
**2c**	200	400	25	100	3.12
**2d**	25	100	12.5	50	1.56
**2e**	12.5	100	12.5	100	1,56
**3a**	400	400	400	800	100
**3b**	400	800	100	NA	200
**3c**	400	400	NA	NA	NA
**3d**	200	400	200	NA	400
**3e**	400	400	400	NA	400
**Ampicillin** ^b^			25	50	25
**Caspofungin** ^b^	25	6.25			

a Tested microorganism.

b Reference drugs.

c NA: Not Active, MIC > 800 μg/mL.

When disk diffusion and MIC analysis results are compared, it is observed that both methods provide consistent results in terms of general antimicrobial activity profiles. The antibacterial and antifungal activities of complexes **2a**, **2b**, **2c**, **2d**, and **2e** along with **3a**, **3b**, **3c**, **3d**, and **3e** against *C. albicans*, *C. glabrata*, *E. coli*, *P. aeruginosa*, and *S. aureus* microorganisms were evaluated using two different methods. While inhibition zone diameters were measured in millimeters in the disk diffusion test, minimum inhibitory concentration values were determined as micrograms per milliliter in the MIC test. Ampicillin was used as the positive control group for bacteria, and caspofungin was used as the positive control group for yeasts.

When the activities of **2a**, **2b**, **2c**, **2d**, and **2e** complexes against *C. albicans* were evaluated, complex **2e** showed the highest inhibition area with a zone diameter of 18.10 ± 0.14 mm in the disk diffusion test, while also demonstrating the lowest MIC value of 12.5 μg/mL in the MIC test. Complex **2d** exhibited the second highest activity with a zone diameter of 16.73 ± 0.17 mm in the disk diffusion test, while following **2e** with a value of 25 μg/mL in the MIC test. In both methods, complexes **2e** and **2d** showed higher antifungal activity than the caspofungin control group (14.30 ± 0.15 mm and 25 μg/mL). Complex **2c** showed moderate activity with 10.20 ± 0.49 mm in the disk diffusion test, while the MIC test with 200 μg/mL supports this finding. Complexes **2a** and **2b** showed the lowest antifungal activity in both tests, having values of 8.20 ± 0.51 mm–400 μg/mL and 8.55 ± 0.32 mm–400 μg/mL, respectively. When the activities of **3a**, **3b**, **3c**, **3d**, and **3e** complexes against *C. albicans* were examined, complex **3d** showed the highest zone diameter with 9.70 ± 0.14 mm in the disk diffusion test, while exhibiting moderate activity with a value of 200 μg/mL in the MIC test. Complex **3e** showed a similar profile with a zone diameter of 9.10 ± 0.37 mm and MIC value of 400 μg/mL. Complexes **3a**, **3b**, and **3c** showed low antifungal activity in both tests, and all these complexes had significantly lower activity than the caspofungin control group.

When the activities of **2a**, **2b**, **2c**, **2d**, and **2e** complexes against *C. glabrata* were evaluated, complex **2d** showed the highest inhibition with a zone diameter of 12.40 ± 0.57 mm in the disk diffusion test, while supporting this result with a value of 100 μg/mL in the MIC test. Complex **2e** similarly exhibited high activity with a zone diameter of 11.97 ± 0.21 mm and MIC value of 100 μg/mL. Both complexes showed lower activity than the caspofungin control group (20.57 ± 0.61 mm and 6.25 μg/mL). Complex **2b** exhibited moderate activity with a zone diameter of 9.10 ± 0.14 mm and MIC value of 400 μg/mL, while complexes **2c** and **2a** showed low activity with values of 9.00 ± 0.24 mm–400 μg/mL and 8.30 ± 0.39 mm–400 μg/mL, respectively. When the activities of **3a**, **3b**, **3c**, **3d**, and **3e** complexes against *C. glabrata* were examined, complexes **3a**, **3c**, **3d**, and **3e** showed zone diameters ranging from 8.83 ± 0.31 mm to 9.00 ± 0.49 mm in the disk diffusion test, while exhibiting values in the range of 200–400 μg/mL in the MIC test. Complex **3b** showed no inhibition in the disk diffusion test, while demonstrating very low activity with 800 μg/mL in the MIC test. All these complexes have significantly lower antifungal activity than the caspofungin control group.

When the activities of **2a**, **2b**, **2c**, **2d**, and **2e** complexes against *E. coli* were evaluated, complex **2d** showed the highest inhibition area with a zone diameter of 19.00 ± 0.45 mm in the disk diffusion test, while demonstrating the lowest MIC value of 12.5 μg/mL in the MIC test. Complex **2e** similarly exhibited high antibacterial activity with a zone diameter of 18.10 ± 0.14 mm and MIC value of 12.5 μg/mL. Both complexes showed higher activity than the ampicillin control group (14.27 ± 0.61 mm and 25 μg/mL). Complex **2c** exhibited similar level of activity to ampicillin with a zone diameter of 16.80 ± 0.24 mm and MIC value of 25 μg/mL, while complex **2b** showed moderate activity with a zone diameter of 14.53 ± 0.45 mm and MIC value of 25 μg/mL. Complex **2a** has the lowest antibacterial activity within this group with a zone diameter of 12.13 ± 0.12 mm and MIC value of 100 μg/mL. When the activities of **3a**, **3b**, **3c**, **3d**, and **3e** complexes against *E. coli* were examined, complex **3b** showed a zone diameter of 11.93 ± 0.17 mm in the disk diffusion test, while exhibiting low-moderate activity with a value of 100 μg/mL in the MIC test. Complexes **3d** and **3a** showed weak activity with values of 9.80 ± 0.22 mm–200 μg/mL and 9.07 ± 0.12 mm–400 μg/mL, respectively. Complex **3e** exhibited low activity with a zone diameter of 8.93 ± 0.17 mm and MIC value of 400 μg/mL, while complex **3c** showed no inhibition in both tests. All these complexes have significantly lower antibacterial activity than the ampicillin control group.

When the activities of **2a**, **2b**, **2c**, **2d**, and **2e** complexes against *P. aeruginosa* were evaluated, complex **2d** showed the highest inhibition with a zone diameter of 13.93 ± 0.19 mm in the disk diffusion test, while demonstrating the lowest MIC value of 50 μg/mL in the MIC test. Complex **2e** showed moderate activity with a zone diameter of 11.97 ± 0.05 mm and MIC value of 100 μg/mL, while complex **2c** exhibited a similar profile with a zone diameter of 10.80 ± 0.22 mm and MIC value of 100 μg/mL. All three complexes showed lower or similar activity compared to the ampicillin control group (12.43 ± 0.42 mm and 50 μg/mL). Complex **2b** exhibited low activity with a zone diameter of 7.93 ± 0.34 mm and MIC value of 400 μg/mL, while complex **2a** showed no inhibition in the disk diffusion test but showed values above 800 μg/mL in the MIC test. All **3a**, **3b**, **3c**, **3d**, and **3e** complexes showed no inhibition against *P. aeruginosa* in both tests. While no zone was formed in the disk diffusion test, values above 800 μg/mL were obtained in the MIC test.

When the activities of **2a**, **2b**, **2c**, **2d**, and **2e** complexes against *S. aureus* were evaluated, complex **2d** showed the highest inhibition area with a zone diameter of 26.73 ± 0.38 mm in the disk diffusion test, while demonstrating the lowest MIC value of 1.56 μg/mL in the MIC test. Complex **2e** similarly exhibited very high antibacterial activity with a zone diameter of 26.33 ± 0.62 mm and MIC value of 1.56 μg/mL. Complex **2c** showed high activity with a zone diameter of 23.67 ± 0.47 mm and MIC value of 3.12 μg/mL, while complex **2b** exhibited a similar profile with a zone diameter of 23.33 ± 0.94 mm and MIC value of 3.12 μg/mL. Although complex **2a** showed the lowest activity within this group with a zone diameter of 21.27 ± 0.92 mm and MIC value of 6.25 μg/mL, all these complexes have significantly higher antibacterial activity than the ampicillin control group (15.33 ± 0.21 mm and 25 μg/mL). When the activities of **3a**, **3b**, **3c**, **3d**, and **3e** complexes against *S. aureus* were examined, complex **3a** showed a zone diameter of 11.90 ± 0.16 mm in the disk diffusion test, while exhibiting low activity with a value of 100 μg/mL in the MIC test. Complex **3b** showed weak activity with a zone diameter of 10.50 ± 0.45 mm and MIC value of 200 μg/mL. Complexes **3d** and **3e** showed zone diameters of 9.03 ± 0.05 mm and 8.83 ± 0.12 mm respectively in the disk diffusion test, while both exhibited very low activity with a value of 400 μg/mL in the MIC test. Complex **3c** showed no inhibition in both tests. All these complexes have significantly lower antibacterial activity than the ampicillin control group.

When the spectrum activities of **2a**, **2b**, **2c**, **2d**, and **2e** complexes were evaluated, it was confirmed by both test methods that complexes **2d** and **2e** showed broad-spectrum activity against both bacteria and yeasts. These complexes exhibited very high selective activity especially against *S. aureus* Gram-positive bacterium, while also showing effective antimicrobial activity against *E. coli* and *P. aeruginosa* Gram-negative bacteria. Both complexes also exhibited antifungal activity against *C. albicans* yeast exceeding the caspofungin control group. Complex **2c** showed high activity against Gram-positive and Gram-negative bacteria, while exhibiting moderate activity against yeasts. Complex **2b** similarly showed antibacterial activity but its antifungal activity remained low. Complex **2a** showed the lowest activity against all microorganisms. When the spectrum activities of **3a**, **3b**, **3c**, **3d**, and **3e** complexes were examined, it was determined by both test methods that these complexes generally have narrow-spectrum and low antimicrobial activity. Complexes **3a** and **3b** showed low-level selective activity against *S. aureus*, while showing very weak or no activity against other microorganisms. Complex **3c** showed only very low activity against yeasts, while being completely inactive against bacteria. Complexes **3d** and **3e** showed very low activity against all tested microorganisms.

When the general evaluation of **2a**, **2b**, **2c**, **2d**, and **2e** complexes was conducted, it was proven by both disk diffusion and MIC tests that complexes **2d** and **2e** demonstrated superior performance against all tested microorganisms. Complex **2d** exhibited the highest inhibition activity in each microorganism with values of 16.73 ± 0.17 mm–25 μg/mL against *C. albicans*, 12.40 ± 0.57 mm–100 μg/mL against *C. glabrata*, 19.00 ± 0.45 mm–12.5 μg/mL against *E. coli*, 13.93 ± 0.19 mm–50 μg/mL against *P. aeruginosa*, and 26.73 ± 0.38 mm–1.56 μg/mL against *S. aureus*. Complex **2e** similarly showed very high antimicrobial activity with values of 18.10 ± 0.14 mm–12.5 μg/mL against *C. albicans*, 11.97 ± 0.21 mm–100 μg/mL against *C. glabrata*, 18.10 ± 0.14 mm–12.5 μg/mL against *E. coli*, 11.97 ± 0.05 mm–100 μg/mL against *P. aeruginosa*, and 26.33 ± 0.62 mm–1.56 μg/mL against *S. aureus*. The broad-spectrum antimicrobial activities of these complexes were supported by both test methods, indicating significant clinical potential due to their effectiveness against pathogens such as *S. aureus*, *E. coli*, *P. aeruginosa*, and *Candida* species frequently encountered in hospital infections. These complexes, which can be considered as alternative treatment options against microorganisms showing multidrug resistance, also draw attention due to their higher activity than current standard antibiotics. When the general evaluation of **3a**, **3b**, **3c**, **3d**, and **3e** complexes was conducted, it was confirmed by both test methods that these complexes showed low antimicrobial activity against all tested microorganisms. These complexes have significantly lower activity than standard control groups, and their clinical use potentials are limited.

When the drug development potentials of **2d** and **2e** complexes are evaluated, it is seen that these compounds are promising candidate molecules due to their broad-spectrum antimicrobial activities and effectiveness exceeding standard antibiotics. In the drug development process, it is necessary to determine minimum inhibition and bactericidal concentrations through microdilution tests, elucidate their mechanisms of action through time-kill kinetics studies, establish biosafety profiles through cytotoxicity tests in cell lines, hemolytic activity analyses, and acute-chronic toxicity studies in animal models, determine absorption-distribution-metabolism-elimination characteristics through pharmacokinetic studies, and conduct target validation studies and resistance development risk analyses. The results obtained are consistent with our other studies^44^

The consistently lower antimicrobial activity observed for the selenium–NHC complexes compared to their benzimidazolium precursors can be attributed to several structure–activity factors. Coordination of selenium to the carbene center alters both the electronic density and steric environment of the ligand, which may reduce the ability of the molecule to interact efficiently with microbial membranes or key enzyme targets. In the parent salts, the positively charged benzimidazolium core is known to enhance membrane association and disruption; however, this cationic character is partially neutralized or redistributed upon formation of the Se–NHC adduct. This reduction in overall charge density and change in molecular geometry likely contributes to the weaker inhibitory profiles observed for all Se–NHC complexes. These findings indicate that selenium incorporation, while beneficial in some NHC frameworks reported in the literature, does not universally enhance potency and may be detrimental for this specific scaffold

A focused structure–activity analysis of the Se–NHC series (**3a–e**) shows that all substituents on the benzene ring are alkyl groups. Within this series, the unsubstituted benzyl derivatives (**3a**) showed the lowest antimicrobial activity, while increasing the size and branching of the alkyl groups led to modest improvements in activity. For example, isopropyl (**3d**) and *tert*-butyl (**3e**) derivatives displayed relatively higher antibacterial and antifungal potency compared to methyl-substituted (**3b, 3c**) or unsubstituted analogues. This trend indicates that steric bulk and hydrophobicity of the alkyl substituents contribute positively to microbial inhibition, likely by facilitating better interaction with microbial membranes. Despite this trend, all Se–NHC complexes exhibited lower activity than their parent benzimidazolium salts, suggesting that selenium coordination modifies electronic density and steric environment in a way that reduces overall antimicrobial efficacy.

### 2.3 Molecular Docking Study

The antimicrobial action of a molecule can manifest through various mechanisms.^47,48^ Among them, the inhibition of essential enzymes for bacterial growth is the main mode of action for many drugs.^48^ To examine the mechanism of action of the molecules prepared against the studied bacterial strains, a molecular docking study was conducted for the molecule **2d** and its derivative **3d**, targeting four key enzymes: DNA gyrase, dihydrofolate reductase (DHFR), tyrosyl-tRNA synthetase (TyrRS), and the fungal sterol 14-α-demethylse (CYP51). DNA gyrase is an essential bacterial type II topoisomerase responsible for introducing ATP-dependent negative supercoils into DNA, a process required for replication, transcription, and maintenance of chromosomal organization. Owing to its indispensable role in bacterial physiology and its absence in higher eukaryotes, DNA gyrase remains an established target for antibacterial agents.^48,49^ (DHFR) is a key enzyme in the folate pathway that catalyzes the reduction of dihydrofolate to tetrahydrofolate, a necessary cofactor for nucleotide biosynthesis and DNA replication. Inhibition of bacterial DHFR disrupts cell proliferation; trimethoprim is a well-known inhibitor acting through this mechanism.^50^ Tyrosyl-tRNA synthetase (TyrRS) is involved in protein biosynthesis by catalyzing the ATP-dependent attachment of tyrosine to its corresponding tRNA. Blocking this aminoacylation step interferes with bacterial translation, making TyrRS an attractive target for the development of new antimicrobial agents.^51^

In addition to these bacterial targets, the fungal sterol 14-α-demethylase (CYP51) was included to evaluate potential interactions relevant to antifungal activity. CYP51 plays a central role in ergosterol biosynthesis, a pathway essential for fungal cell membrane integrity, and is therefore a major target of several clinical antifungal agents. These enzymes participate in essential biological processes, and their inhibition can stop microbial growth and reproduction, making them important targets for the development of new antibiotic treatments.^48,49,52,53^

The docking results are summarized in Table 4 and illustrated in Figures 1–4. For all studied enzymes, compounds **2d** and **3d** showed negative binding energies ranging from −4.60 to −7.39 kcal/mol, indicating a favorable affinity toward the target proteins and suggesting their potential as inhibitors. When compared with their respective native ligands, the binding energies of compounds **2d** and **3d** were comparable to that of gepotidacin for DNA gyrase, but slightly lower than those of methotrexate for DHFR and SB-239629 for TyrRS, and markedly lower than that of posaconazole for CYP51. These results suggest that the inhibitory potential of the tested compounds is weaker than that of the native ligands across all targets.

**Figure 1:**
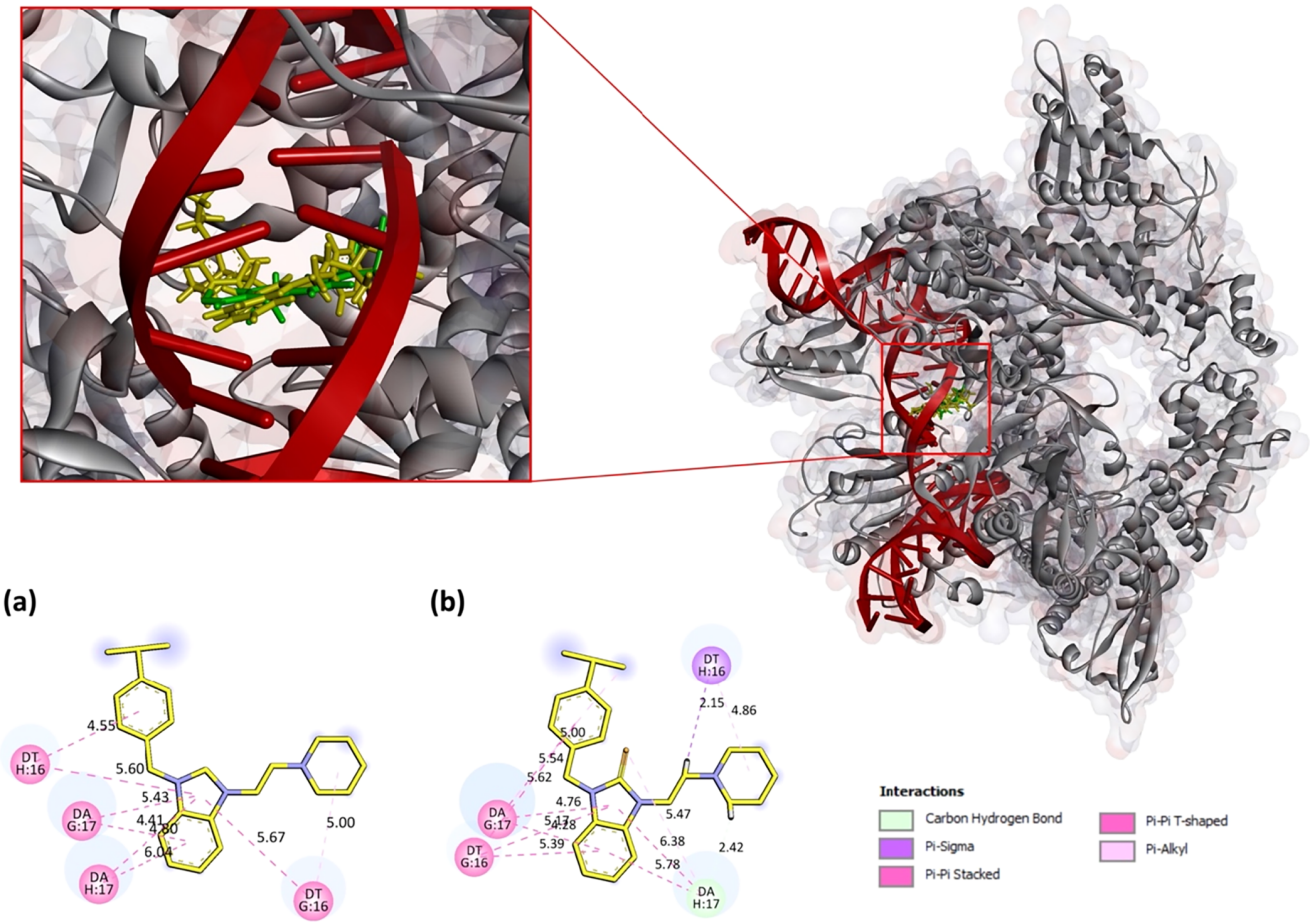
Interaction modes of **2d** (a) and **3d** (b) into the active site of DNA Gyrase enzyme.

The results obtained are presented in Table 5 and Figures 1–3. For the three enzymes studied, the molecules **2d** and **3d** exhibit negative binding energy values, ranging from −4.60 to −5.60 kcal/mol. This indicates a good affinity of the two compounds for the three enzymes, making them potential inhibitors. Compared to native ligands, the binding energy of **2d** and **3d** is comparable to that of gepotidacin for DNA gyrase but slightly lower than that of the native ligands of DHFR and TyrRS.

**Table 5:** Calculated Binding Affinities Obtained from Molecular Docking of Benzimidazolium Salt **2d** and Its Corresponding Selenium–NHC Complex **3d** against DNA Gyrase, DHFR, TyrRS, and CYP51

	docking binding energy in kcal/mol			
compound	DNA gyrase	DHFR	TyrRS	CYP51
**2d**	–5.60	–5.08	–5.53	–6.77
**3d**	–4.60	–5.71	–5.99	–7.39
Native ligand^a^	–5.69	–7.81	–6.16	–9.94

a Gepotidacin for DNA Gyrase, methotrexate for DHFR, SB-239629 (a specific inhibitor) for TyrRS, and posaconazole for CYP51.

Figure 1 Predicted binding conformations of compound **2d** (a) and its Se–NHC derivative **3d** (b) within the catalytic pocket of DNA gyrase.

For the enzyme DHFR, the binding modes of **2d** and **3d** are shown in Figure 2. Similar to DNA gyrase, the most stable orientations of the two molecules overlap with the native ligand, methotrexate, in the enzyme’s active site. Methotrexate forms several hydrogen bonds with the residues ARG57, LYS32, ILE94, and ARG52, as well as hydrophobic interactions with PHE31, ILE5, ALA7, LEU28, and ILE50. Most of these amino acids are also involved in interactions with the compounds **2d** and **3d**. Selenium–NHC compound **3d** forms hydrophobic bonds and carbon–hydrogen bond interactions with the residues ALA7 and PHE31, while benzimidazolium salt **2d** can interact with ARG52, LEU28, ILE50, ILE94, PHE31, and LYS32.

**Figure 2:**
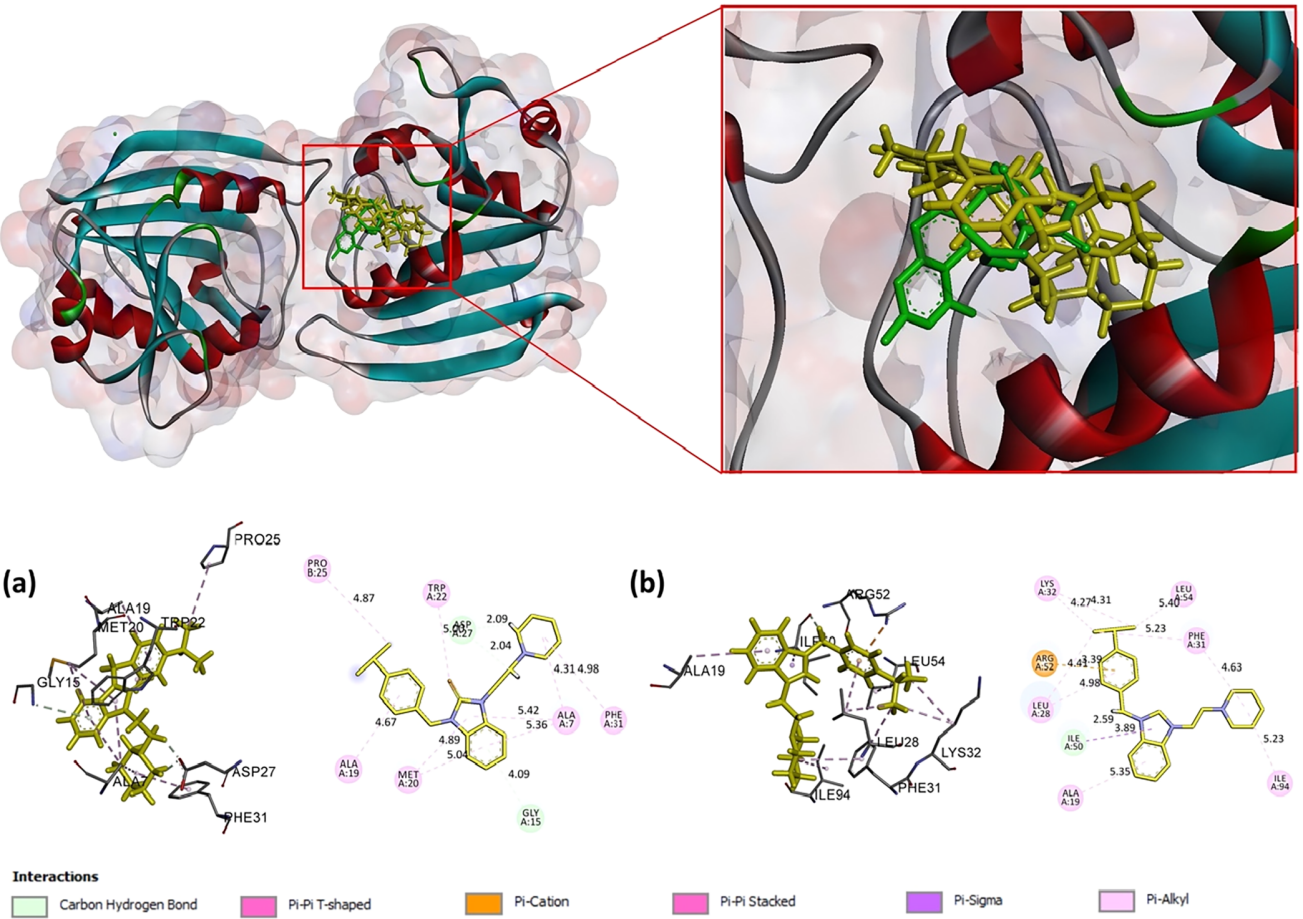
Predicted binding conformations of compound **2d** (a) and its Se–NHC derivative **3d** (b) within the active site of DHFR enzyme.

Figure 3 illustrates the interaction modes of **2d** and **3d** with the enzyme TyrRS. Similar to interactions observed with other enzymes, the most stable orientations of **2d** and **3d** are located in the same region as the native ligand. The native ligand can establish hydrogen or hydrophobic interactions with several amino acids, including CYS37, GLN196, GLY193, ASP195, TYR170, ASP80, TYR36, LEU70, GLY38, PHE54, HSD50, and ALA39. Compounds **2d** and **3d** are also capable of interacting with several of these amino acids. In particular, the salt **2d** can bind with HSD50, ALA39, ASP195, and GLY38, while the complex **3d** interacts with ASP195, LEU70, TYR36, and GLY38.

**Figure 3:**
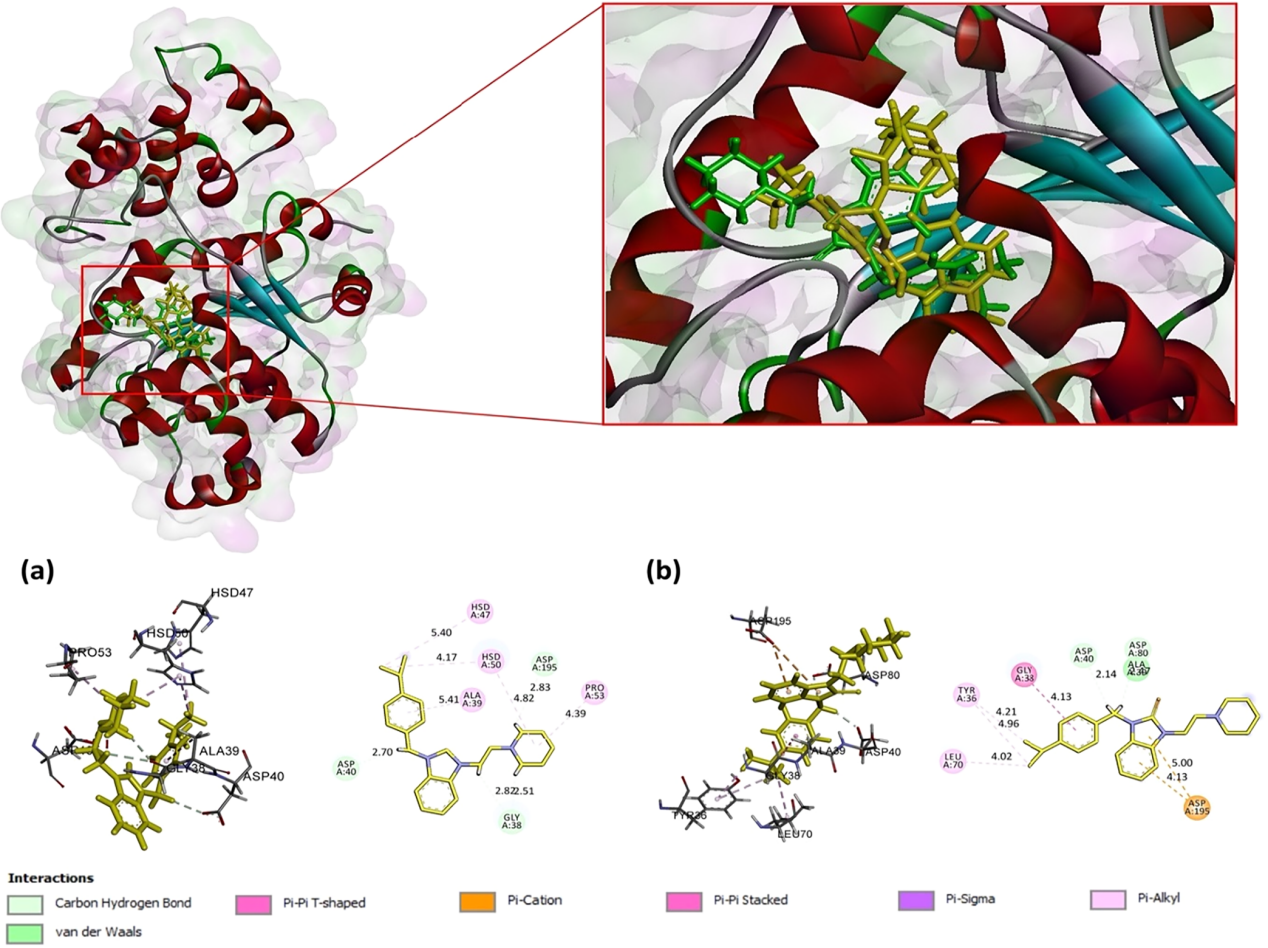
Predicted binding conformations of compound **2d** (a) and its Se–NHC derivative **3d** (b) within the active site of TyrRS enzyme.

Analysis of the interaction modes with the three bacterial enzymes shows that the full molecular frameworks of both compounds participate in binding, including the benzimidazole core and the aromatic rings. In addition, the selenium atom of compound **3d** contributes to favorable interactions in the cases of DNA gyrase and DHFR. However, despite the presence of Se, complex **3d** does not display an enhanced binding affinity compared with the benzimidazolium salt **2d**, nor does it establish stronger or more significant interactions within the active sites of the three enzymes. This indicates that selenium complexation does not further improve the inhibitory potential of these molecules, which is consistent with the antimicrobial activity results.

The interaction modes of the fungal enzyme CYP51, illustrated in Figure 4, show that compound **2d** forms several hydrophobic interactions within the active site, involving its aromatic rings and the tetrahydropyridine moiety. The compound also forms a favorable interaction with the catalytic heme group. However, a nonfavorable interaction with the heme was also detected, which may explain its lower binding energy compared with compound **3d**. In contrast, compound **3d** displays a more extensive network of interactions, including both hydrophobic contacts and hydrogen bonds. The selenium atom participates in multiple interactions, notably including a key contact with the heme group. This additional stabilization likely accounts for the higher binding energy of **3d** relative to **2d**, suggesting that the Se complex may act as a more potent CYP51 inhibitor.

**Figure 4:**
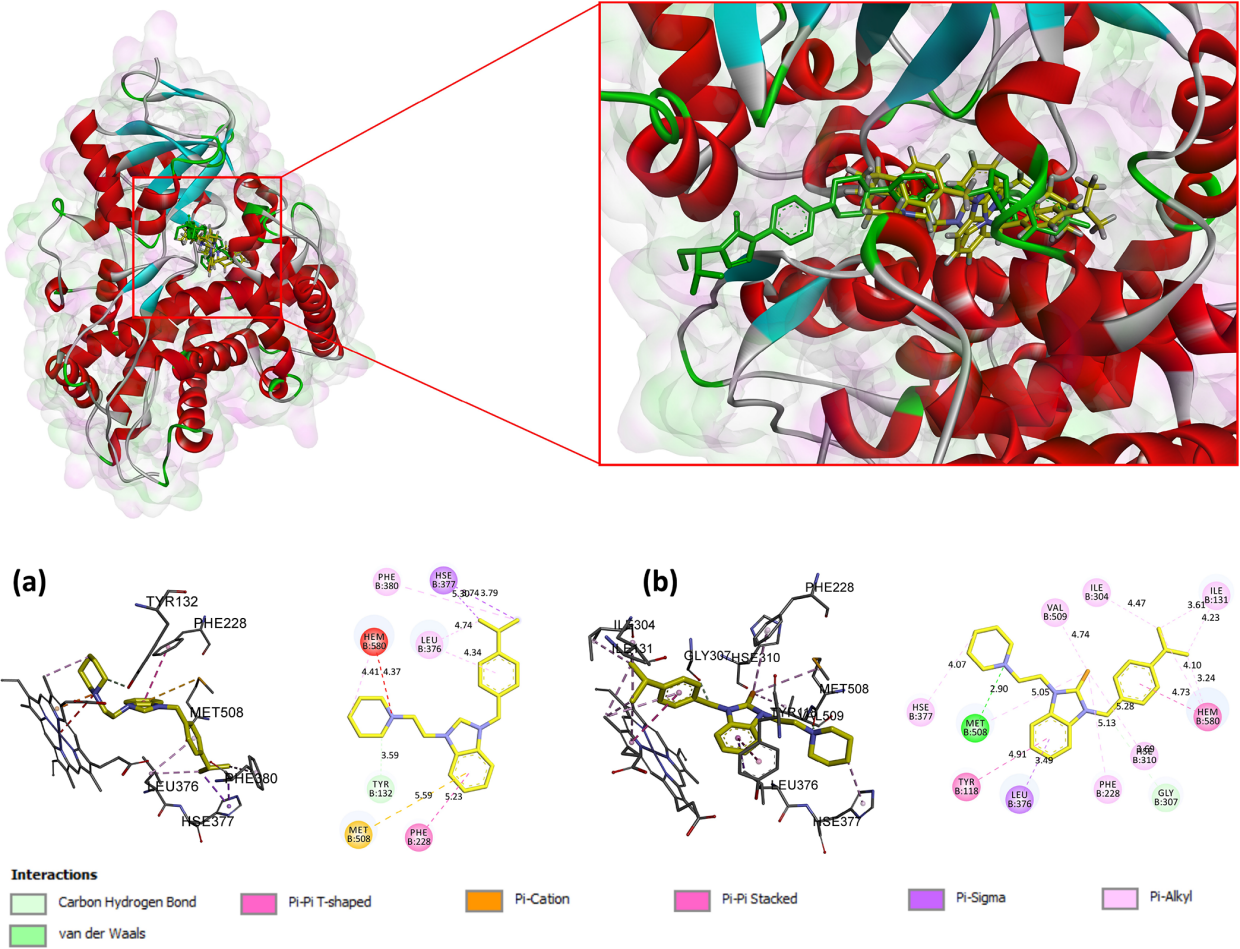
Predicted binding conformations of compound **2d** (a) and its Se–NHC derivative **3d** (b) within the active site of CYP51 enzyme.

Overall, the docking results demonstrate that both **2d** and 3d exhibit appreciable affinity toward the three bacterial enzymes as well as the fungal CYP51 enzyme, forming stabilizing interactions with essential amino acids in their active sites. These findings support the potential of the two compounds as inhibitors of DNA gyrase, DHFR, TyrRS, and CYP51.

## 3 Conclusions

In this study, we synthesized and characterized a series of benzimidazolium salts (**2a**–**e**) and their selenium-NHC adducts **(3a-e)**, evaluating their antimicrobial and antifungal activities. The benzimidazolium salts, especially compounds **2d** and **2e**, exhibited superior antimicrobial efficacy against *C. albicans*, *E. coli*, and *S. aureus*, outperforming their selenium-NHC adducts. Molecular docking studies revealed that compounds **2d** and **3d** had strong affinities for bacterial enzymes (DNA gyrase, dihydrofolate reductase, and tyrosyl-tRNA synthetase) and the fungal enzyme CYP51, suggesting that their antimicrobial effects may stem from interfering with essential microbial processes.

Notably, the selenium-NHC complexes did not show enhanced activity against bacterial enzymes compared to the benzimidazolium salts. However, the Se–NHC complexes exhibited superior binding affinity to the fungal enzyme CYP51, highlighting the potential for these compounds to selectively target fungal pathogens.

Antimicrobial activities were evaluated using both disk diffusion and MIC methods, with consistent results across both. Complexes **2d** and **2e** showed the highest activity against all microorganisms tested, outperforming controls like ampicillin and caspofungin, particularly in their antibacterial effect against S. aureus and antifungal effect against C. albicans. Complexes **2c** and 2b showed moderate-to-high activity, while **2a** exhibited low activity. In contrast, Se–NHC complexes (**3a**–**e**) generally displayed lower antimicrobial activity and were ineffective against P. aeruginosa. While the methods (disk diffusion and MIC) are based on different principles, the results were scientifically compatible, confirming the general antimicrobial profiles. The broad-spectrum efficacy of complexes **2d** and **2e**, particularly against multidrug-resistant pathogens, suggests their potential as alternative therapeutic agents for hospital infections.

Further studies, including toxicological assessments, pharmacokinetics, and mechanism of action research, are needed to assess their drug development potential. In conclusion, benzimidazolium salts, especially **2d** and **2e**, are promising candidates for new antimicrobial agents. Future work should focus on optimizing their efficacy for clinical applications and exploring the structure–activity relationship (SAR) differences between the salts and Se–NHC complexes.

## 4 Experimental Section

### 4.1 Materials and Methods

The synthesis of selenium-*N*-Heterocyclic carbene compounds and their corresponding benzimidazolium salts was performed under an argon atmosphere in flame-dried glassware using Schlenk techniques. The reagents utilized were obtained from Merck, Sigma-Aldrich, and Fluka.

Using a PerkinElmer Spectrum 100, FT-IR spectra were obtained with an ATR unit, covering the range of 400–4000 cm^–1^. Mass spectrometry analysis, conducted at the Inönü University research center, provided molecular weights and fragmentation patterns of the synthesized compounds. NMR analyses for ^1^H and ^13^C were recorded on a Varian As 400 Merkur spectrometer, with ^1^H at 400 MHz and ^13^C at 101 MHz, in CDCl_3_ and tetramethylsilane as the internal standard. Coupling constants (*J* values) are reported in Hertz. Signal multiplicities in the NMR analyses are indicated as follows: s = singlet, d = doublet, t = triplet, sept = septet, q = quartet, and m = multiplet. Melting points were determined using a Stuart SMP 40 apparatus with open capillary tubes, and the values were uncorrected.

### 4.2 General Synthetic Procedure for Benzimidazolium Chloride Salts **2a–e**

Benzimidazolium chloride salts **2a–e** were prepared using a modified literature method.^54,55^ In a typical experiment, 1-(2-(piperidin-1-yl)­ethyl)-1*H*-benzo­[*d*]­imidazole (1 equiv) and the appropriate alkyl Chloride (1 equiv) were dissolved in degassed toluene (4–5 mL). The reaction mixture was heated at 80 °C under an argon atmosphere and stirred for 48 h.

After the reaction period, the solvent was removed under reduced pressure. Addition of diethyl ether (50 mL) induced formation of a precipitate, which was isolated by filtration. The solid product was washed successively with diethyl ether (3 × 20 mL) and dried under vacuum. Final purification was achieved by recrystallization from a dichloromethane/diethyl ether mixture (1:3, v/v) at ambient temperature to afford pure benzimidazolium chloride salts.

#### 4.2.1 1-(2-(Piperidin-1-yl)­ethyl)-3-benzylbenzimidazolium Chloride (**2a**)

Yield 81% (1265 mg, beige solid); mp = 180–182 °C; FT-IR ν­(C–N) = 1554 cm^–1^. ^1^H NMR (400 MHz, CDCl_3_, TMS, 25 °C): δ (ppm) = δ 11.41 (s, 1H, NCHN); 7.77 (d, *J* = 8.1 Hz, 1H, CH-Ar); 7.57 (d, *J* = 8.4 Hz, 2H, CH-Ar); 7.52 (d, *J* = 7.8 Hz, 1H, CH-Ar); 7.50–7.28 (m, 5H, CH-Ar); 5.86 (s, 2H, N–CH_2_-Ph); 4.73 (t, *J* = 5.6 Hz, 2H, N–CH_2_-CH_2_); 2.87 (t, *J* = 5.5 Hz, 2H, N–CH_2_–CH_2_); 1.51–1.30 (m, 6H). ^13^C NMR (100 MHz, CDCl_3_, TMS, 25 °C): δ (ppm) = 143.2 (NCHN); 132.0 (C_q_, C-CH_2_–N); 130.6 (C_,_ CH); 130.0 (C_,_ CH); 128.3 (2C_,_ CH); 128.1 (C_,_ CH); 127.2 (2C_,_ CH); 125.8 (2C_,_ CH); 112.5 (C, CH); 112.2 (C, CH); 55.6 (C, Ph-N-CH_2_); 53.5 (2C, CH_2_–N-CH_2_); 50.1 (C, N–CH_2_–CH_2_); 43.9 (C, N-CH_2_); 24.7 (2C, CH_2_); 22.9 (C, CH_2_). C_21_H_26_ClN_3_ (Mw = 355.91 g/mol) HRMS (ESI**)** *m*/*z* calcd [M – Cl]^+^ 320.2127, found 320.2101.

#### 4.2.2 1-(2-(Piperidin-1-yl)­ethyl)-3-(4-methylbenzyl)­benzimidazolium Chloride (**2b**)

Yield 76% (1220 mg, white solid); mp = 172–173 °C; FT-IR ν­(C–N) = 1558 cm^–1^. ^1^H NMR (400 MHz, CDCl_3_, TMS, 25 °C): δ (ppm) = δ 11.41 (s, 1H, NCHN); 7.75 (d, *J* = 8.3 Hz, 1H, CH-Ar); 7.62–7.54 (m, 2H, CH-Ar); 7.50 (dd, *J* = 14.3, 6.5 Hz, 1H, CH-Ar); 7.35 (d, *J* = 8.1 Hz, 2H- CH-Ar); 7.12 (d, *J* = 7.9 Hz, 2H, CH-Ar); 5.78 (s, 2H, CH_2_, N–CH_2_-Ph); 4.70 (t, *J* = 5.6 Hz, 2H, CH_2_, N–CH_2_-CH_2_); 2.84 (t, *J* = 5.6 Hz, 2H, CH_2_, N–CH_2_–CH_2_); 2.27 (s, 3H, CH_3_); 2.44 (s, 4H, 2CH_2_, CH_2_–N-CH_2_); 1.49–1.31 (m, 6H, 3CH_2_, NCH_2_–CH_2_-CH_2_-CH_2_-CH_2_N). ^13^C NMR (100 MHz, CDCl_3_, TMS, 25 °C): δ (ppm) = 144.0 (NCHN); 139.1 (C_q_, CH_2_–C-CH_2_); 131.6 (C_q_, C-CH_2_–N); 131.0 (C_,_ CH); 130.0 (C_,_ CH); 129.9 (2C_,_ CH); 128.2 (C_,_ CH); 126.8 (2C_,_ CH); 125.8 (2C_,_ CH); 113.5 (C, CH); 113.2 (C, CH); 55.7 (C, Ph-N-CH_2_); 54.5 (2C, CH_2_–N-CH_2_); 51.0 (C, N–CH_2_–CH_2_); 45.1 (C, N-CH_2_); 25.8 (2C, CH_2_); 23.9 (C, CH_2_); 21.1 (C, CH_3_). C_22_H_28_ClN_3_ (Mw = 369.93 g/mol) HRMS (ESI**)** *m*/*z* calcd [M – Cl]^+^ 334.2283, found 334.2256.

#### 4.2.3 1-(2-(Piperidin-1-yl)­ethyl)-3-(2,4,6-trimethylbenzyl)­benzimidazolium Chloride (**2c**)

Yield 75% (1308 mg, white solid); mp = 232–233 °C; FT-IR ν­(C–N) = 1561 cm^–1^. ^1^H NMR (400 MHz, CDCl_3_, TMS, 25 °C): δ (ppm) = δ 10.41 (s, 1H, NCHN); 7.80 (d, *J* = 8.3 Hz, 1H, CH–Ar); 7.58–7.52 (m, 1H, CH–Ar); 7.50–7.44 (m, 1H, CH–Ar); 7.41 (d, *J* = 8.3 Hz, 1H, CH–Ar); 6.91 (s, 2H, 2CH–Ar); 5.74 (s, 2H, CH_2_, N–CH_2_-Ph); 4.73 (t, *J* = 5.1 Hz, 2H, CH_2_, N–CH_2_-CH_2_); 2.75 (t, *J* = 5.6 Hz, 2H, CH_2_, N–CH_2_–CH_2_); 2.36 (s, 4H, 2CH_2_, CH_2_–N-CH_2_); 2.30 (s, 6H, 2CH_3_); 2.26 (s, 3H, CH_3_); 1.39–1.21 (m, 6H, 3CH_2_, NCH_2_–CH_2_-CH_2_-CH_2_-CH_2_N). ^13^C NMR (100 MHz, CDCl_3_, TMS, 25 °C): δ (ppm) = 143.9 (NCHN); 139.7 (C_q_); 138.0 (2C_q_); 131.6 (C_q_, C-CH_2_–N); 131.1 (C_,_ CH); 130.1 (4C_,_ 4CH); 126.9 (2C_,_ CH); 125.3 (2C_,_ CH); 113.3 (C, CH); 113.3 (C, CH); 56.6 (C, Ph-N-CH_2_); 54.3 (2C, CH_2_–N-CH_2_); 46.6 (C, N–CH_2_–CH_2_); 44.6 (C, N-CH_2_); 25.6 (2C, CH_2_); 24.0 (C, CH_2_); 21.0 (C, CH_3_); 20.2 (2C, 2CH_3_). C_24_H_32_ClN_3_ (Mw = 397.98 g/mol) HRMS (ESI**)** *m*/*z* calcd [M – Cl]^+^ 362.2596, found 362.2566.

#### 4.2.4 1-(2-(Piperidin-1-yl)­ethyl)-3-(4-*iso*propylbenzyl)­benzimidazolium Chloride (**2d**)

Yield 70% (980 mg, beige solid); mp = 156–158 °C; FT-IR ν­(C–N) = 1563 cm^–1^. ^1^H NMR (400 MHz, CDCl_3_, TMS, 25 °C): δ (ppm) = δ 11.28 (s, 1H, NCHN); 7.77 (d, *J* = 7.9 Hz, 1H, CH–Ar); 7.62 (d, *J* = 7.5 Hz, 1H, CH–Ar); 7.59–7.47 (m, 2H, CH–Ar); 7.39 (d, *J* = 8.2 Hz, 2H, CH–Ar); 7.18 (d, *J* = 8.2 Hz, 2H, CH–Ar); 5.78 (s, 2H, CH_2_, N–CH_2_-Ph); 4.73 (t, *J* = 5.1 Hz, 2H, CH_2_, N–CH_2_-CH_2_); 2.84 (t, *J* = 5.6 Hz, 2H, CH_2_, N–CH_2_–CH_2_); 2.45 (s, 4H, 2CH_2_, CH_2_–N-CH_2_); 1.52–1.30 (m, 6H, 3CH_2_, NCH_2_–CH_2_-CH_2_-CH_2_-CH_2_N); 1.18 (s, 6H, 2CH_3_). ^13^C NMR (100 MHz, CDCl_3_, TMS, 25 °C): δ (ppm) = 150.0 (C_q_); 144.0 (NCHN); 131.6 (C_q_, C-CH_2_–N); 131.0 (C_,_ CH); 130.3 (C_,_ CH); 128.3 (4C_,_ 4CH); 127.3 (4C_,_ 4CH); 126.8 (2C_,_ CH); 126.8 (2C_,_ CH); 113.5 (C, CH); 113.2 (C, CH); 56.6 (C, Ph-N-CH_2_); 54.4 (2C, CH_2_–N-CH_2_); 50.9 (C, N–CH_2_–CH_2_); 44.9 (C, N-CH_2_); 33.8 (C, CH); 25.7 (2C, CH_2_); 23.9 (C, CH_2_); 23.8 (2C, 2CH_3_). C_24_H_32_ClN_3_ (Mw = 397.98 g/mol) HRMS (ESI**)** *m*/*z* calcd [M – Cl]^+^ 362.2596, found 362.2567.

#### 4.2.5 1-(2-(Piperidin-1-yl)­ethyl)-3-(4-*tert*-butylbenzyl)­benzimidazolium Chloride (**2e**)

Yield 77% (1120 mg, beige solid); mp = 258–260 °C; FT-IR ν­(C–N) = 1561 cm^–1^. ^1^H NMR (400 MHz, CDCl_3_, TMS, 25 °C): δ (ppm) = 11.22 (s, 1H, NCHN); 7.76 (d, *J* = 7.8 Hz, 1H, CH–Ar); 7.62 (d, *J* = 7.8 Hz, 1H, CH–Ar); 7.59–7.46 (m, 2H, CH–Ar); 7.40 (d, *J* = 8.1 Hz, 2H, CH–Ar); 7.34 (d, *J* = 8.1 Hz, 2H, CH–Ar); 5.77 (s, 2H, CH_2_, N–CH_2_-Ph); 4.73 (t, *J* = 5.3 Hz, 2H, CH_2_, N–CH_2_-CH_2_); 2.84 (t, *J* = 5.6 Hz, 2H, CH_2_, N–CH_2_–CH_2_); 2.45 (s, 4H, 2CH_2_, CH_2_–N-CH_2_); 1.50–1.30 (m, 6H, 3CH_2_, NCH_2_–CH_2_-CH_2_-CH_2_-CH_2_N); 1.24 (s, 9H, 3CH_3_). ^13^C NMR (100 MHz, CDCl_3_, TMS, 25 °C): δ (ppm) = 152.3 (C_q_); 143.9 (NCHN); 131.5 (C_q_, C-CH_2_–N); 131.0 (C_,_ CH); 130.0 (C_,_ CH); 128.0 (4C_,_ 4CH); 126.8 (2C_,_ CH); 126.2 (4C_,_ 4CH); 113.5 (C, CH); 113.2 (C, CH); 56.6 (C, Ph-N-CH_2_); 54.4 (2C, CH_2_–N-CH_2_); 50.8 (C, N–CH_2_–CH_2_); 44.9 (C, N-CH_2_); 34.6 (C, CH); 31.1 (3C, 3CH_3_); 25.7 (2C, CH_2_); 23.9 (C, CH_2_). C_25_H_34_ClN_3_ (Mw = 412.01 g/mol) HRMS (ESI**)** *m*/*z* calcd [M – Cl]^+^ 376.5570, found 376.2672.

### 4.3 General Synthetic Procedure for Selenium–NHC Complexes **3a–e**

Selenium–NHC complexes **3a–e** were prepared via in situ generation of the corresponding *N*-heterocyclic carbenes from benzimidazolium chloride salts using a procedure adapted from our previously reported method.^39,44^ In a typical experiment, the appropriate benzimidazolium chloride salt (1 equiv), selenium powder (1 equiv), and potassium carbonate (1.5 equiv) were suspended in methanol and heated at 80 °C under stirring for 48 h, leading to the formation of a black solid corresponding to the selenium–carbene adduct. After cooling to room temperature, the crude product was dissolved in dichloromethane and purified by flash column chromatography on silica gel. Removal of the solvent under reduced pressure followed by recrystallization from a chloroform/diethyl ether mixture afforded the selenium–NHC complexes as pure black solids.

#### 4.3.1 1-(2-(Piperidin-1-yl)­ethyl)-3-benzylbenzimidazole-2-selenone (**3a**)

Yield 70% (140 mg, Yellow solid); mp = 126 °C; FT-IR ν­(CN) = 1154 cm^–1^. ^1^H NMR (400 MHz, CDCl_3_, TMS, 25 °C): δ (ppm) = 7.45–7.29 (m, 7H, CH–Ar); 7.09 (s, 2H, CH-Ar); 5.71 (s, 2H, N–CH_2_-Ph); 4.62 (t, *J* = 5.6 Hz, 2H, N–CH_2_-CH_2_); 2.78 (t, *J* = 5.5 Hz, 2H, N–CH_2_–CH_2_); 2.45 (s, 4H, 2CH_2_, CH_2_–N-CH_2_); 1.65–1.42 (m, 6H, 3CH_2_, NCH_2_–CH_2_-CH_2_-CH_2_-CH_2_N). ^13^C NMR (100 MHz, CDCl_3_, TMS, 25 °C): δ (ppm) = 167.2 (C=Se); 135.4 (Cq,); 133.4 (C, CH); 132.9 (C, CH); 128.7 (4C, CH); 127.8 (2C, CH); 127.5 (4C, CH); 123.8 (2C, CH); 110.2­(C, CH); 109.9 (C, CH); 56.1 (C, Ph-N–CH_2_); 54.9 (2C, CH_2_–N-CH_2_); 50.2 (C, N–CH_2_–CH_2_); 44.7 (C, N–CH_2_); 26.1 (2C, CH_2_); 24.4 (C, CH_2_). C_21_H_25_N_3_Se (Mw = 398.40 g/mol) HRMS (ESI) *m*/*z* calcd [M] 399.1214, found 399.1218.

#### 4.3.2 1-(2-(Piperidin-1-yl)­ethyl)-3-(4-methylbenzyl)­benzimidazole-2-selenone (**3b**)

Yield 72% (150 mg, beige solid); mp = 118 °C; FT-IR ν­(CN) = 1192 cm^–1^. ^1^H NMR (400 MHz, CDCl_3_, TMS, 25 °C): δ (ppm) = 7.33 (d, *J* = 7.9 Hz, 1H, CH-Ar); 7.26 (s, 4H, CH-Ar); 7.24–7.19 (m, 1H, CH-Ar); 7.18–7.12 (m, 1H, CH-Ar); 7.10 (d, *J* = 8.1 Hz, 1H, CH-Ar); 5.67 (s, 2H, N–CH_2_-Ph); 4.61 (t, *J* = 7.3 Hz, 2H, N–CH_2_-CH_2_); 2.77 (t, *J* = 7.3 Hz, 2H, N–CH_2_–CH_2_); 2.57 (s, 4H, 2CH_2_, CH_2_–N-CH_2_); 2.29 (s, 3H, CH_3_); 1.63–1.40 (m, 6H, 3CH_2_, NCH_2_–CH_2_-CH_2_-CH_2_-CH_2_N). ^13^C NMR (100 MHz, CDCl_3_, TMS, 25 °C): δ (ppm) = 167.0 (C = Se); 135.5 (Cq,); 133.4 (C, CH); 132.9 (C, CH); 132.8 (C, CH); 129.4 (2C, CH); 127.5 (2C, CH); 123.2 (C, CH); 110.2­(C, CH); 109.8 (C, CH); 56.1 (C, Ph-N–CH_2_); 54.9 (2C, CH_2_–N-CH_2_); 49.9 (C, N–CH_2_–CH_2_); 44.6 (C, N–CH_2_); 25.9 (2C, CH_2_); 24.2 (C, CH_2_); 21.1 (C, CH_3_). C_22_H_27_N_3_Se (Mw = 412.43 g/mol) HRMS (ESI) *m*/*z* calcd [M] 413.1370, found 413.3191.

#### 4.3.3 1-(2-(Piperidin-1-yl)­ethyl)-3-(2,4,6-trimethylbenzyl)­benzimidazole-2-selenone (**3c**)

Yield 81% (178 mg, beige solid); mp = 164–165 °C; FT-IR ν­(CN) = 1203 cm^–1^. ^1^H NMR (400 MHz, CDCl_3_, TMS, 25 °C): δ (ppm) = 7.30 (d, *J* = 8.3 Hz, 1H, CH-Ar); 7.19–7.08 (m, 1H, CH-Ar); 6.97–6.89 (m, 1H, CH-Ar), 6.87 (s, 2H, CH-Ar), 6.45 (d, *J* = 8.3 Hz, 1H, CH-Ar); 5.73 (s, 2H); 4.61 (s, 2H); 2.76 (s, 2H); 2.55 (s, 4H), 2.28 (s, 3H), 2.21 (s, 6H), 1.56 (s, 4H), 1.43 (s, 2H). ^13^C NMR (100 MHz, CDCl_3_, TMS, 25 °C): δ (ppm) = 166.4 (C = Se); 137.9 (Cq,); 137.6 (2Cq,); 133.3 (C, Cq); 132.7 (C, Cq); 128.7 (2C, CH); 128.3 (1C, CH); 122.9 (1C, CH); 122.8 (1C, CH); 110.5­(C, CH); 109.6 (C, CH); 56.1 (C, Ph-N-CH_2_); 54.9 (2C, CH_2_–N-CH_2_); 48.1 (C, N–CH_2_–CH_2_); 44.6 (C, N-CH_2_); 25.9 (2C, CH_2_); 24.2 (C, CH_2_); 20.9 (C, CH_3_): 20.4 (2C, CH_3_). C_24_H_31_N_3_Se (Mw = 440.48 g/mol) HRMS (ESI) *m*/*z* calcd [M + H]^+^ 442.1756, found 442.1699.

#### 4.3.4 1-(2-(Piperidin-1-yl)­ethyl)-3-(4-*iso*propylbenzyl)­benzimidazole-2-selenone (**3d**)

Yield 75% (165 mg, Yellow solid); mp = 80–81 °C; FT-IR ν­(CN) = 1198 cm^–1^. ^1^H NMR (400 MHz, CDCl_3_, TMS, 25 °C): δ (ppm) = 7.34 (d, *J* = 7.9 Hz, 1H, CH-Ar); 7.26 (dd, *J* = 26.7 Hz, 1H, CH-Ar); 7.16 (s, 4H, CH–Ar); 7.06- 6.98 (m, 1H, CH-Ar); 6.90 (d, *J* = 7.7 Hz 1H, CH-Ar); 5.67 (s, 2H, CH_2_); 4.62 (s, 2H, CH_2_); 2.78 (s, 2H, CH_2_); 2.57 (s, 4H, 2CH_2_); 1.59 (s, 4H, 2CH_2_); 1.45 (s, 2H, CH_2_); 1.21 (s, 6H, 2CH_3_). ^13^C NMR (100 MHz, CDCl_3_, TMS, 25 °C): δ (ppm) = 167.1 (C = Se); 148.5 (Cq,); 133.4 (C, CH); 133.0 (C, CH); 132.7 (C, CH); 127.5 (2C, CH); 126.8 (2C, CH); 1236 (C, CH); 110.3­(C, CH); 109.8 (C, CH); 56.1 (C, Ph-N–CH_2_); 54.9 (2C, CH_2_–N-CH_2_); 49.9 (C, N–CH_2_–CH_2_); 44.7 (C, N–CH_2_); 33.7 (Cq, isopropyl); 26.0 (2C, CH_2_); 24.5 (C, CH_2_); 23.9 (2C, CH_3_). C_24_H_31_N_3_Se (Mw = 440.48 g/mol) HRMS (ESI) *m*/*z* calcd [M – H]^+^ 440.1605, found 440.1711.

#### 4.3.5 1-(2-(Piperidin-1-yl)­ethyl)-3-(4-(*tert*-butyl)­benzyl)­benzimidazole-2-selenone (**3e**)

Yield 73% (167 mg, Yellow solid); mp = 130–132 °C; FT-IR ν­(CN) = 1198 cm^–1^. ^1^H NMR (400 MHz, CDCl_3_, TMS, 25 °C): δ (ppm) = 7.34 (d, *J* = 7.6 Hz, 1H, CH-Ar); 7.31 (s, 4H, CH-Ar); 7.25 −7.10 (m, 3H, CH-Ar); 6.17 (s, 2H, CH-Ar), 5.67 (s, 2H, N–CH_2_-Ph); 4.62 (t, *J* = 7.3 Hz, 2H, N–CH_2_-CH_2_); 2.76 (t, *J* = 13.8 Hz, 2H, N–CH_2_–CH_2_); 2.57 (s, 4H, 2CH_2_, CH_2_–N-CH_2_); 1.65–1.40 (m, 6H, 3CH_2_, NCH_2_–CH_2_-CH_2_-CH_2_-CH_2_N); 1.24 (s, 9H, 3CH_3_). ^13^C NMR (100 MHz, CDCl_3_, TMS, 25 °C): δ (ppm) = 166.4 (C = Se); 137.9 (Cq,); 137.6 (2Cq,); 133.3 (C, Cq); 132.7 (C, Cq); 128.7 (2C, CH); 128.3 (1C, CH); 122.9 (1C, CH); 122.8 (1C, CH); 110.5­(C, CH); 109.6 (C, CH); 56.1 (C, Ph-N-CH_2_); 54.9 (2C, CH_2_–N-CH_2_); 49.8 (C, N–CH_2_–CH_2_); 44.6 (C, N-CH_2_); 34.5 (Cq, ter-but); 31.2 (3C, CH_3_); 26.0 (2C, CH_2_); 24.2 (C, CH_2_). C_25_H_33_N_3_Se (Mw = 454.50 g/mol) HRMS (ESI) *m*/*z* calcd calcd [M + H]^+^ 456.1913, found 456.1850.

### 4.4 Biological Capacity Study

#### 4.4.1 Material

Chemical materials used include Peptone (PanReac AppliChem), Glucose (Difko), pure water, Tryptone (PanReac AppliChem), NaCl (PanReac AppliChem), Dimethyl sulfoxide (DMSO) (Fisher Scientific), Yeast extract (PanReac AppliChem), and Agar (PanReac AppliChem). For antimicrobial activity experiments conducted in the Laboratory of the Department of Medical Genetics at the School of Medicine of Inonu University, equipment utilized comprised the Denovix DS-11 FX+ (UV, Blue, Red, Green) Spectrophotometer/Fluorometer, Allsheng AMR-100 Microplate Reader, Daihan WIS 20 Shaking Incubator, Nüve EN 120 Incubator, Nüve NF 800R Cooled Centrifuge, and Sigma 1–14 Microcentrifuge.

#### 4.4.2 Methods

To determine the effectiveness of the compounds in inhibiting bacterial and fungal growth, disc diffusion and broth microdilution (BMD) methods were employed. The bacterial strains tested included *S. aureus* (ATCC 29213), *E. coli* (ATCC 25922), and *P. aeruginosa* (ATCC 27853), while the fungal strains included *C. albicans* (SC5314/ATCC MYA-2876) and *C. glabrata* (ATCC 2001). All microorganisms were obtained from the Laboratory of the Medical Biology and Genetics Department of Inonu University Faculty of Medicine (Battalgazi, Malatya, Turkey). For the disc diffusion assay, each compound was dissolved in 100% DMSO to achieve a concentration of 80 μg/μL, and 800 μg of compound per disc was used. Bacteria (approximately 1 × 10^8^ cells) were inoculated in sterile LB broth medium, and yeasts (approximately 1 × 10^7^ cells) were inoculated in YPD broth medium, then spread on Petri dishes under aseptic conditions. Compound-loaded discs were placed in 90 mm diameter Petri dishes and incubated at 37 °C for 24 h. A disc containing only DMSO served as the negative control, while Ampicillin (800 μg/disc) and Caspofungin (800 μg/disc) were used as standard antibiotic and antifungal agents, respectively. Antimicrobial activity was evaluated by measuring the diameter of the inhibition zone (mm). MIC (Minimum Inhibitory Concentration) analyses were performed using the BMD test according to EUCAST EDef 7.3.2 for yeasts and CLSI M07-A10 for bacteria. Stock solutions of the compounds were prepared in 100% DMSO and serial dilutions were made in flat-bottom 96-well plates using SDB (Sabouraud Dextrose Broth) medium (pH 5.6) for yeasts and LB (Luria–Bertani) broth medium (pH 7.0) for bacteria. Yeast (1–5 × 10^5^ CFU/mL) and bacterial (1 × 10^6^ CFU/mL) cell suspensions prepared in sterile water were added in equal volumes to wells containing different concentrations of the compounds. Final compound concentrations ranged from 0.8 to 800 μg/μL, while cell concentrations were adjusted to 0.5–2.5 × 10^5^ CFU/mL for yeasts and 5 × 10^5^ CFU/mL for bacteria. Plates were incubated at 37 °C for 24 h for yeasts and 16–18 h for bacteria. For yeasts, the MIC value was determined spectrophotometrically at 530 nm after incubation and defined as the lowest compound concentration causing at least 50% growth inhibition compared to the control; for bacteria, it was determined by visual analysis as the lowest concentration showing no visible growth

### 4.5 Molecular Docking

Molecular docking investigations were carried out using LeDock software to evaluate the binding affinity of the optimized compounds toward selected biological targets. The three-dimensional structures of the enzymes—*E. coli* DNA gyrase (PDB ID: 6RKU), *E. coli* dihydrofolate reductase (DHFR, PDB ID: 4DFR), *S. aureus* tyrosyl-tRNA synthetase (TyrRS, PDB ID: 1JIJ), and sterol 14-α-demethylase (CYP51, PDB ID: 5FSA)—were obtained from the Protein Data Bank. Prior to docking, protein structures were preprocessed by eliminating crystallographic water molecules, cocrystallized ligands, heteroatoms, and solvent residues. Hydrogen atoms and partial atomic charges were subsequently assigned using the LePro utility available at http://www.lephar.com The docking region was defined as a cubic grid box of 25 Å per side with a grid spacing of 1 Å, positioned at the catalytic sites of each enzyme. The grid center coordinates were set as follows: DNA gyrase (158.1506, 158.7073, 146.7502), DHFR (18.4407, 68.7857, 42.6854), TyrRS (−11.4226, 14.9726, 85.9476), and CYP51 (187.7610, 19.8410, 73.921). To ensure the reliability of the docking protocol, validation was performed through redocking of the cocrystallized ligands into their respective binding pockets. The calculated root-mean-square deviation (RMSD) values for 1JIJ, 6RKU, 4DFR, and 5FSA were 0.914 Å, 1.690 Å, 2.040 Å, and 1.470 Å respectively, all of which fall within the acceptable range for reliable docking (≤2 Å), confirming the validity of the applied docking procedure (Figure 5). Molecular interaction diagrams were generated using BIOVIA Discovery Studio. Prior to docking simulations, the molecular geometries of all investigated compounds were optimized using density functional theory (DFT) at the B3LYP/6–31 level of theory using the Gaussian 09 package.^56^

**Figure 5:**
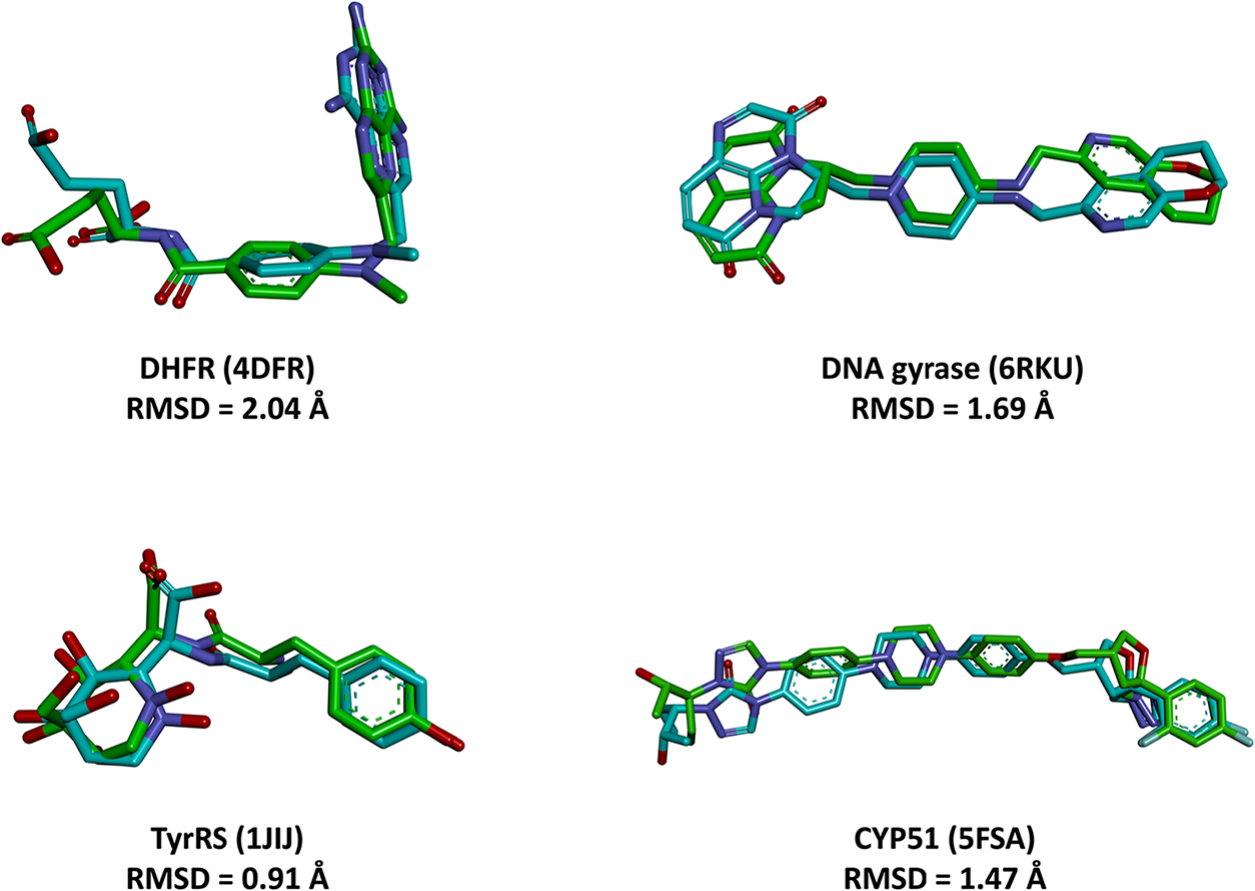
Redocking validation of the docking protocol showing the superimposition of crystallographic (cyan) and predicted poses (green) of the native ligands for the target enzymes (1JIJ, 6RKU, 4DFR, and 5FSA).

## Supplementary Material



## Notes

Cartesian coordinates of compounds **2d** and **3d** computed in the gas-phase at B3LYP/6–31G level (Table S1); ^1^H and ^13^C NMR spectrum of compounds (Figures S1–S20); mass spectrum of compounds (Figures S20–S29); IR spectrum of compounds (Figures S30–S39); disk diffusion test pictures of the molecules against against *C. albicans* and *C. glabrata* yeasts, *E. coli*, *S. aureus* and *P. aeruginosa* bacteria (Csp: caspofungin, Amp: Ampicillin) (Figure S40) (PDF)
