# Supplementary material for: Synthesis, Docking Profiles, and Biological Insights into Selenium-NHC Adducts and Benzimidazolium Salts with Antimicrobial Potential
Source: ACS Omega. 2026 Jun 1;11(23):33380–94. doi: 10.1021/acsomega.5c10880 (PMC13280680; doi:10.1021/acsomega.5c10880)
Supplement: Supplementary file 1 [file ao5c10880_si_001.pdf]

Supporting information

Synthesis, Docking Profiles, and Biological Insights into Selenium-NHC Adducts and Benzimidazolium Salts with Antimicrobial Potential

Boutheina Boualia,[1,3] Abd el-Krim Sandeli,[2] Houssem Boulebd,[3] Hüseyin Karci,[4] Muhammed Dündar,[5] İlknur Özdemir,[4,6] Nevin Gürbüz,[4,6] Ahmet Koç,[7] İsmail Özdemir*[4,6]

[1]The molecular and structural environmental chemistry research unit, Faculty of Exact Sciences, Brother's Mentouri Constantine 1 University, Constantine, ALGERIA. [2] Pharmaceutical Science Research Center, Constantine, ALGERIA. [3]Laboratory of Synthesis of Molecules with Biological Interest, Frères Mentouri Constantine 1 University, Constantine 25017, Algeria. [4]İnönü University Drug Administration and Research Center, 44280-Malatya, TÜRKİYE. [5]Inönü University, Faculty of Arts and Sciences, Department of Molecular Biology and Genetics, 44280-Malatya, TÜRKİYE. [6]Inönü University, Faculty of Arts and Sciences, Department of Chemistry, 44280-Malatya, TÜRKİYE. [7]Inönü University Faculty of Medicine, Department of Medical Genetics, 44280-Malatya, TÜRKİYE.

Correspondence: Prof. Ismail Özdemir (ismail.ozdemir@inonu.edu.tr)

Table S1. Cartesian coordinates of compounds **2d** and **3d** computed in the gas-phase at B3LYP/6-31G level.

2d

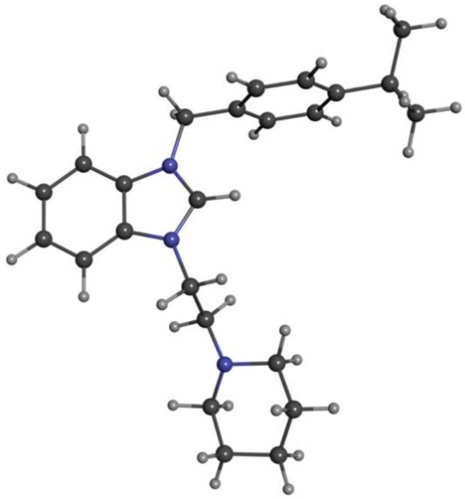

C	-1.38917700	5.24335500	-0.11647000
C	-2.60639000	4.59285400	0.19176600
C	-2.66508700	3.21921400	0.42437800
C	-1.45943300	2.51597500	0.33782400
C	-0.24462900	3.16555100	0.03082800
C	-0.18615300	4.54358900	-0.20175800
N	-1.14935100	1.15407300	0.51846000
C	0.17522600	0.99145600	0.32447100
N	0.75191500	2.17226500	0.03155800
C	-2.11815900	0.07101500	0.80127700
C	-2.77983800	-0.48719700	-0.47896600
N	-3.69365500	-1.56544400	-0.11424000
C	-4.96527100	-1.60509200	-0.87564200
C	-5.95675700	-2.57745600	-0.22148400
C	-5.34050600	-3.98176600	-0.07744800
C	-3.99798200	-3.90336100	0.67274000
C	-3.05242500	-2.89523800	0.00378800
C	2.20735200	2.41139000	-0.23439700
C	3.01697300	1.13904500	-0.24306100
C	3.61995800	0.66549600	0.93623800
C	4.36984500	-0.51233000	0.92551700
C	4.54434500	-1.25204000	-0.26019900
C	3.93916900	-0.76938300	-1.43420500
C	3.18697200	0.40844000	-1.43073600
C	5.37406300	-2.53142200	-0.28219200
C	6.84993600	-2.26181100	0.09421400
C	4.76255600	-3.62624000	0.62326800
H	-1.39280600	6.31321800	-0.28890300

H	-3.51637400	5.17854500	0.24914500
H	-3.60147700	2.72901400	0.66101200
H	0.74278900	5.04936600	-0.43485700
H	0.70402000	0.05717700	0.39709900
H	-1.58558500	-0.71862000	1.33651000
H	-2.88371000	0.46573900	1.47157200
H	-3.34487200	0.31392000	-0.96781800
H	-1.99454300	-0.80547900	-1.19144900
H	-5.38435200	-0.59279100	-0.89359300
H	-4.78925000	-1.91030000	-1.92644300
H	-6.22899700	-2.19034400	0.76906200
H	-6.87328100	-2.61704800	-0.82230000
H	-6.03195500	-4.64990300	0.44855600
H	-5.17797600	-4.41486600	-1.07561400
H	-4.17281900	-3.58878400	1.70992600
H	-3.51403800	-4.88700000	0.70500800
H	-2.13606900	-2.79486800	0.59807600
H	-2.75230900	-3.27094500	-0.99578600
H	2.26486600	2.92225500	-1.20046700
H	2.55915500	3.10008000	0.54035000
H	3.51678300	1.22829500	1.86048200
H	4.83461400	-0.85102000	1.84555500
H	4.07072200	-1.31632100	-2.36275500
H	2.74617700	0.76732400	-2.35719800
H	5.35890300	-2.90808200	-1.31424300
H	7.43521000	-3.18394800	0.01071000
H	7.29887900	-1.51151200	-0.56511800
H	6.93832900	-1.90299000	1.12656100
H	5.34394000	-4.55107500	0.54300800
H	3.72816500	-3.84886200	0.33846800
H	4.76797000	-3.32067900	1.67650000
**3d**			

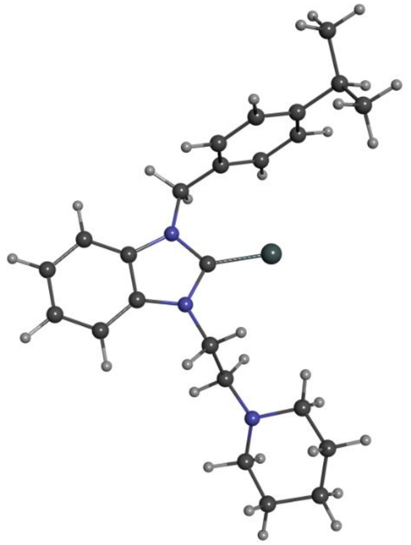

C	0.72708200	5.24014300	-0.48102400
C	1.95247500	4.73153300	-0.92873000
C	2.22556200	3.35142800	-0.89050700
C	1.23112700	2.50935200	-0.39883000
C	-0.00998700	3.02253000	0.04864800
C	-0.27715400	4.38925100	0.01770600
N	1.18030300	1.11777000	-0.24020800
C	-0.04923600	0.72433900	0.31688500
N	-0.77266900	1.92202800	0.46099200
C	2.21129000	0.17514000	-0.65565000
C	3.17927700	-0.20373900	0.48346600
N	4.15794500	-1.20310500	0.03018300
C	5.49141200	-1.08683300	0.65394100
C	6.50775800	-1.98348700	-0.06901200
C	6.02957900	-3.44779400	-0.09734700
C	4.60938100	-3.53649100	-0.68739100
C	3.64967000	-2.59132100	0.05050400
C	-2.04057300	1.95839900	1.18239800
C	-2.94394200	0.80348000	0.78882900
C	-3.51124700	0.73560500	-0.49955000
C	-4.37406900	-0.30111900	-0.84336500
C	-4.69414100	-1.32031000	0.07952900
C	-4.12630100	-1.25489200	1.35654300
C	-3.25933300	-0.20790900	1.71505700
C	-5.62217700	-2.46881700	-0.30255100
C	-7.03582200	-1.96842100	-0.67895100
C	-5.01874700	-3.33131100	-1.43577600
H	0.54560900	6.30933800	-0.51302100
H	2.70981900	5.41042600	-1.30631500
H	3.18039300	2.96442200	-1.22814200
H	-1.22096800	4.79370500	0.36617600

H	1.70125400	-0.71730500	-1.04611600
H	2.78223300	0.61047000	-1.48140200
H	3.71977100	0.69783300	0.79321300
H	2.60086400	-0.54705700	1.36221600
H	5.80548800	-0.03854800	0.59701500
H	5.46136800	-1.36292200	1.72954800
H	6.62869800	-1.61668700	-1.09685800
H	7.48368800	-1.90485000	0.42733900
H	6.72598100	-4.06816300	-0.67545600
H	6.02219200	-3.84777500	0.92809200
H	4.63282800	-3.25557200	-1.74859900
H	4.22863100	-4.56402600	-0.62679200
H	2.66424900	-2.61454300	-0.42576900
H	3.51314500	-2.94328400	1.09506400
H	-1.84952800	1.91889600	2.26541200
H	-2.52567900	2.91419600	0.96047200
H	-3.26578400	1.50208000	-1.22875200
H	-4.79472000	-0.33053200	-1.84364500
H	-4.35748000	-2.02591200	2.08548300
H	-2.86166400	-0.15859100	2.72347000
H	-5.72259300	-3.11067300	0.58414400
H	-7.69568300	-2.81599700	-0.89864500
H	-7.48176000	-1.38890700	0.13696400
H	-7.00574100	-1.32913000	-1.56960200
H	-5.66761200	-4.18853900	-1.65131900
H	-4.02782200	-3.70724200	-1.16000600
H	-4.91190400	-2.75022000	-2.35976000
Si	-0.57768800	-1.02316300	0.76427700

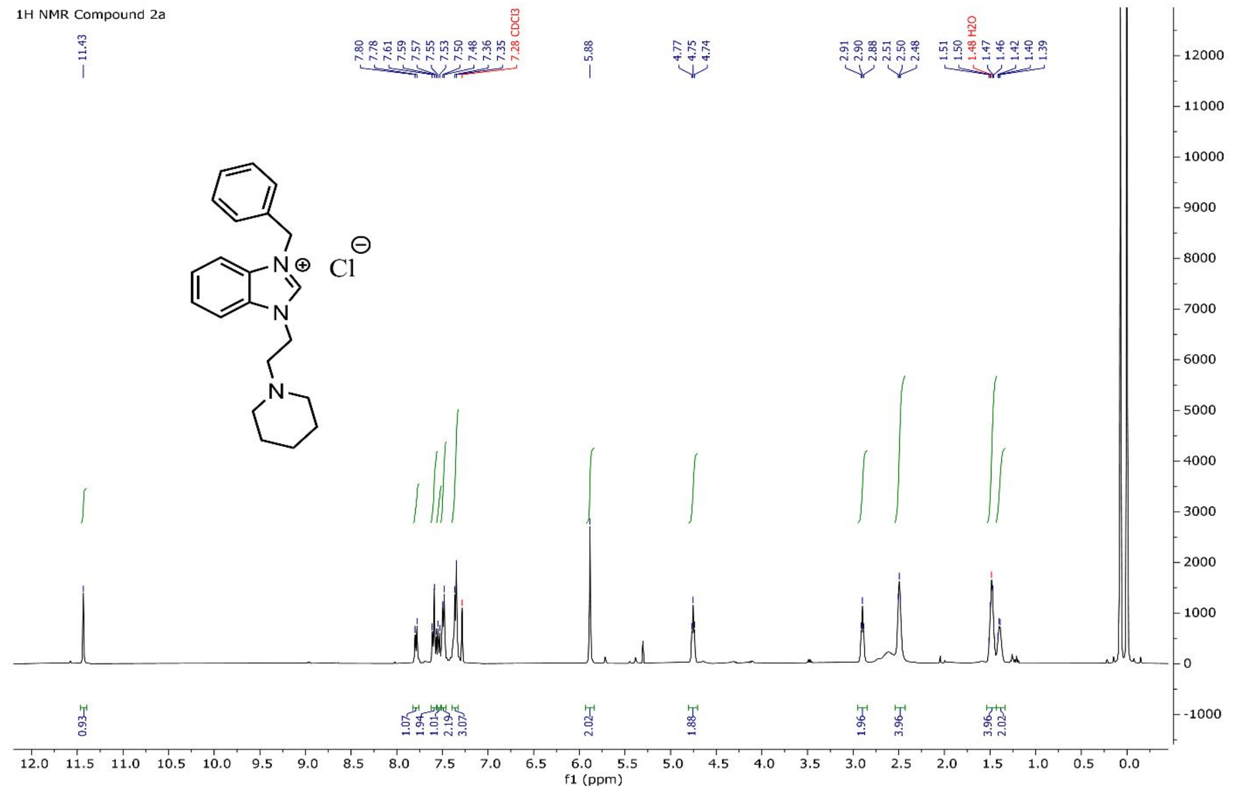

Figure S1. ^{1}H NMR spectrum of compound **2a** ($CDCl_3$)

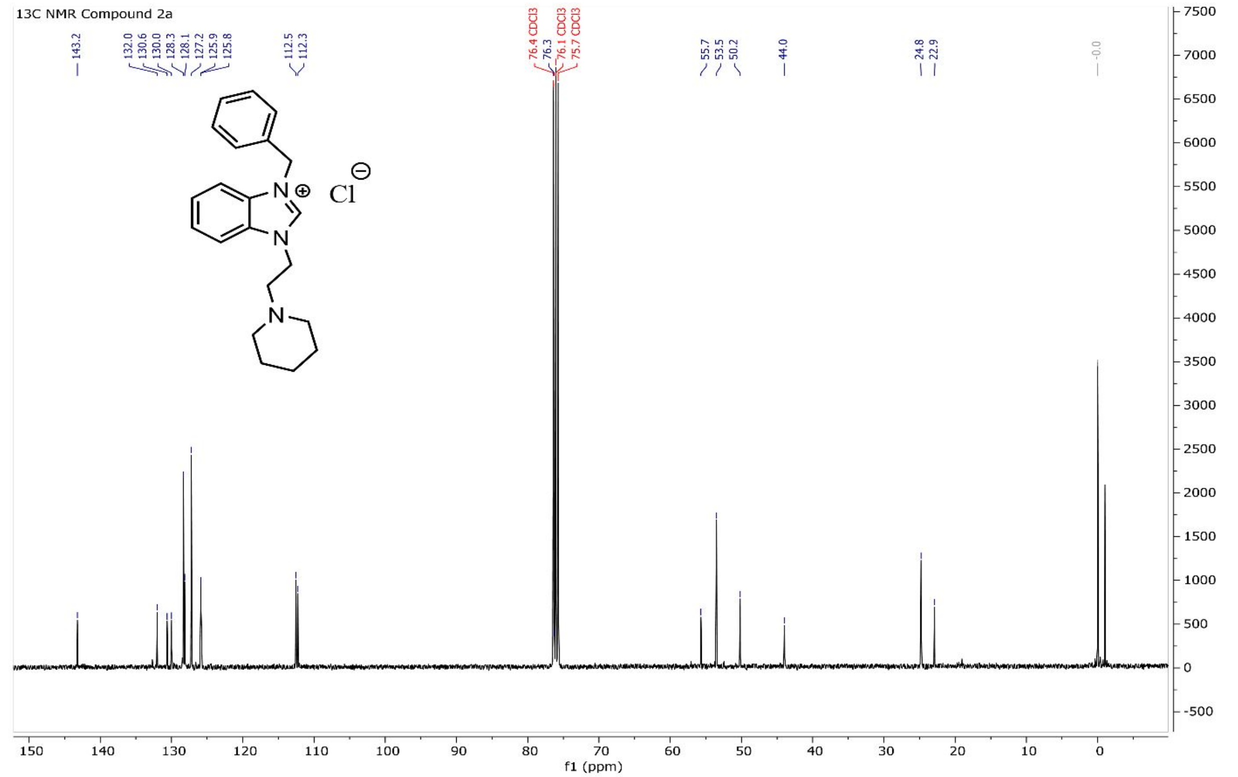

Figure S2. ^{13}C NMR spectrum of compound **2a** ($CDCl_3$)

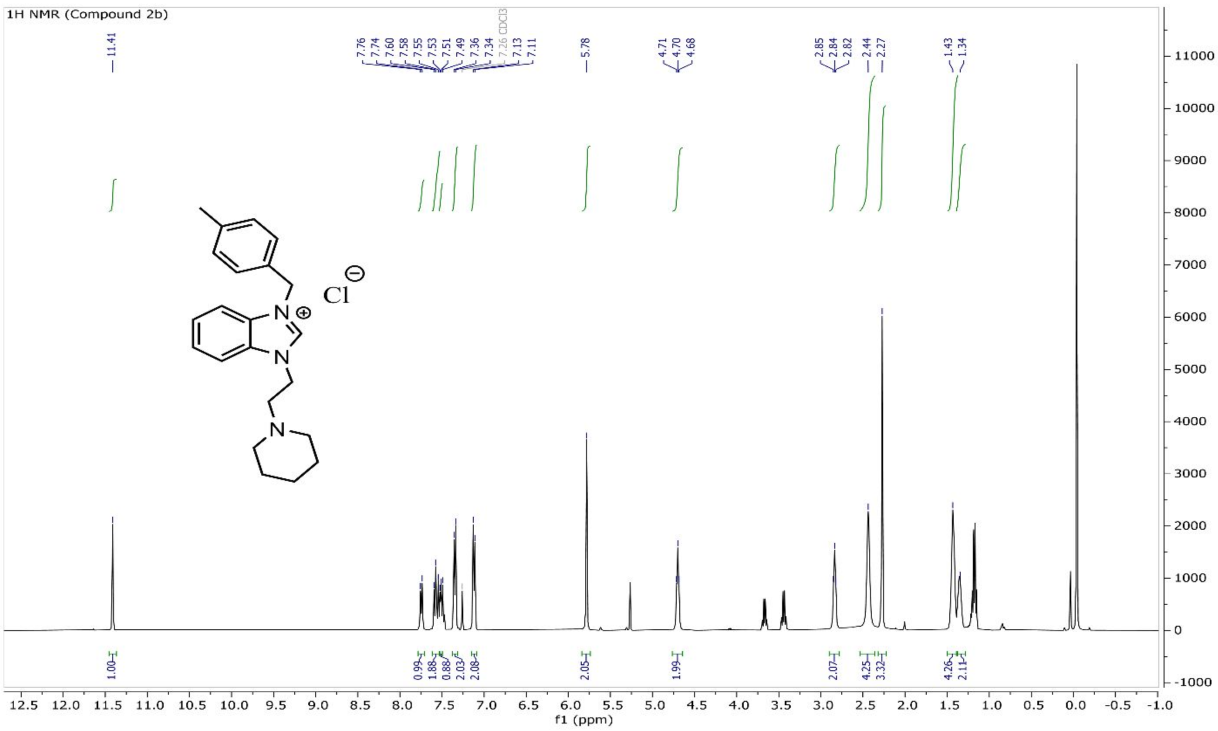

Figure S3. ^{1}H NMR spectrum of compound **2b** ($CDCl_3$)

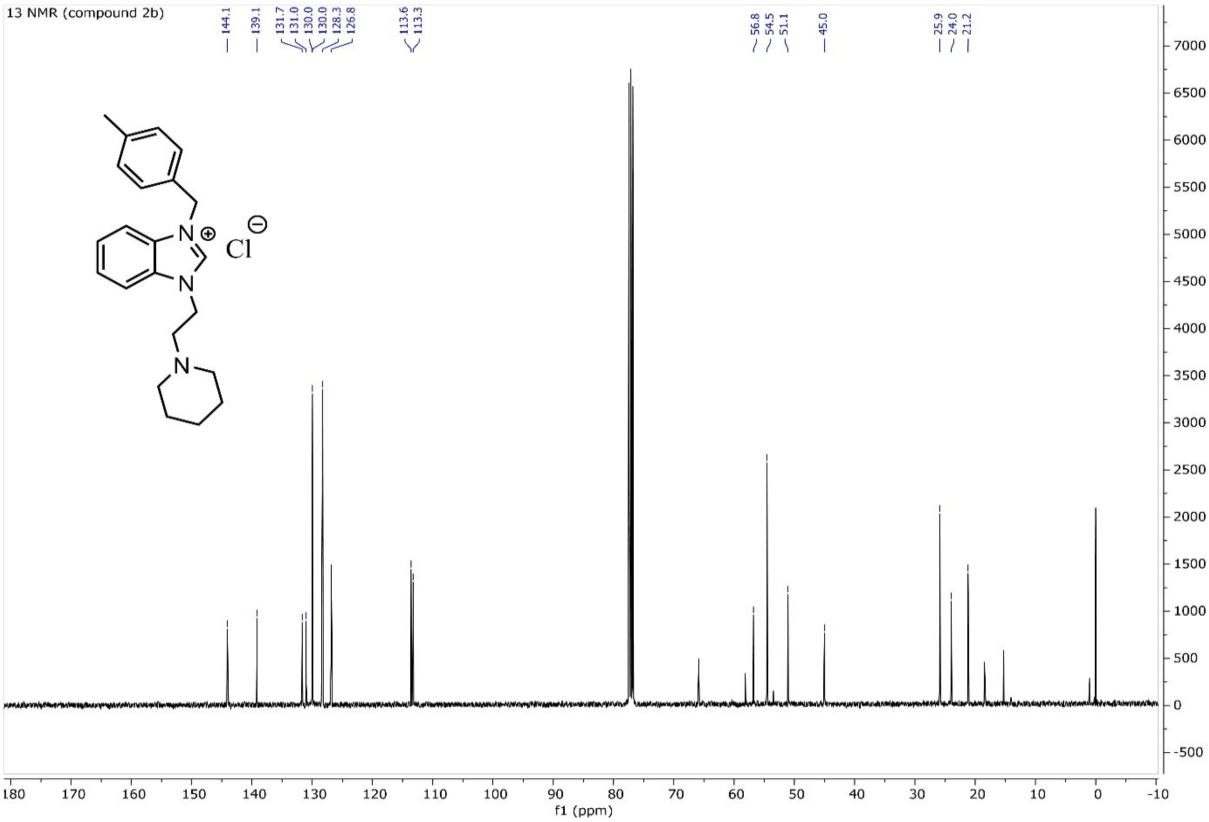

Figure S4. ^{13}C NMR spectrum of compound **2b** ($CDCl_3$)

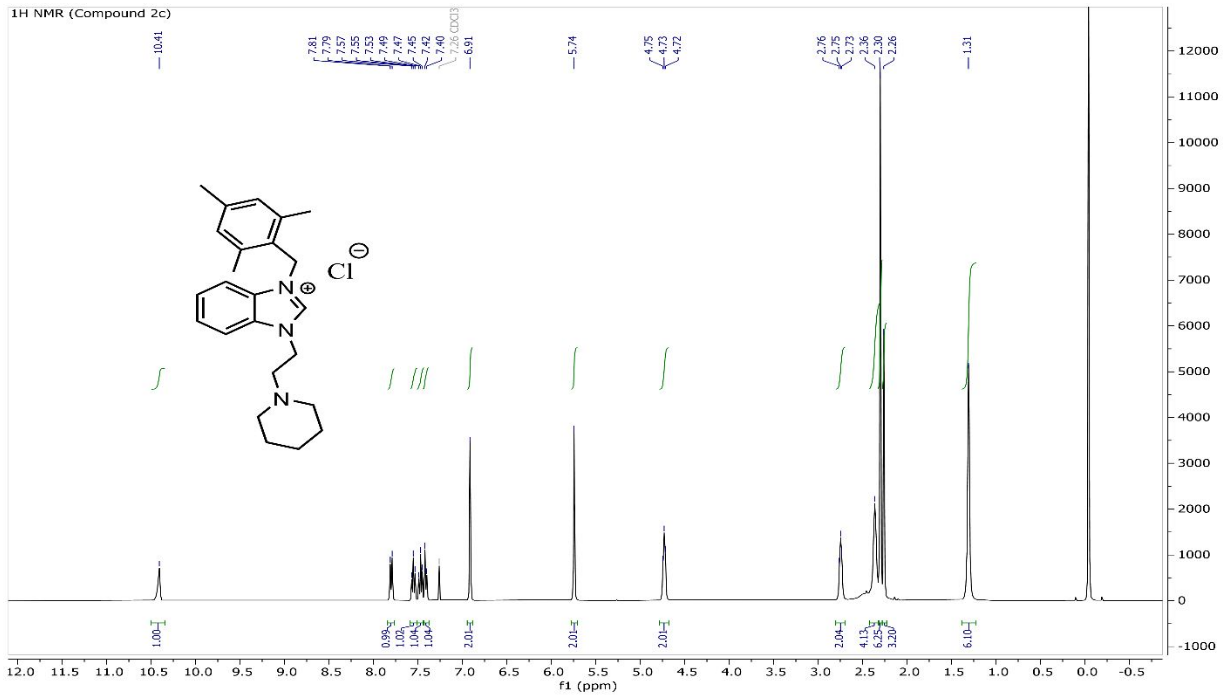

Figure S5. ^{1}H NMR spectrum of compound **2c** ($CDCl_3$)

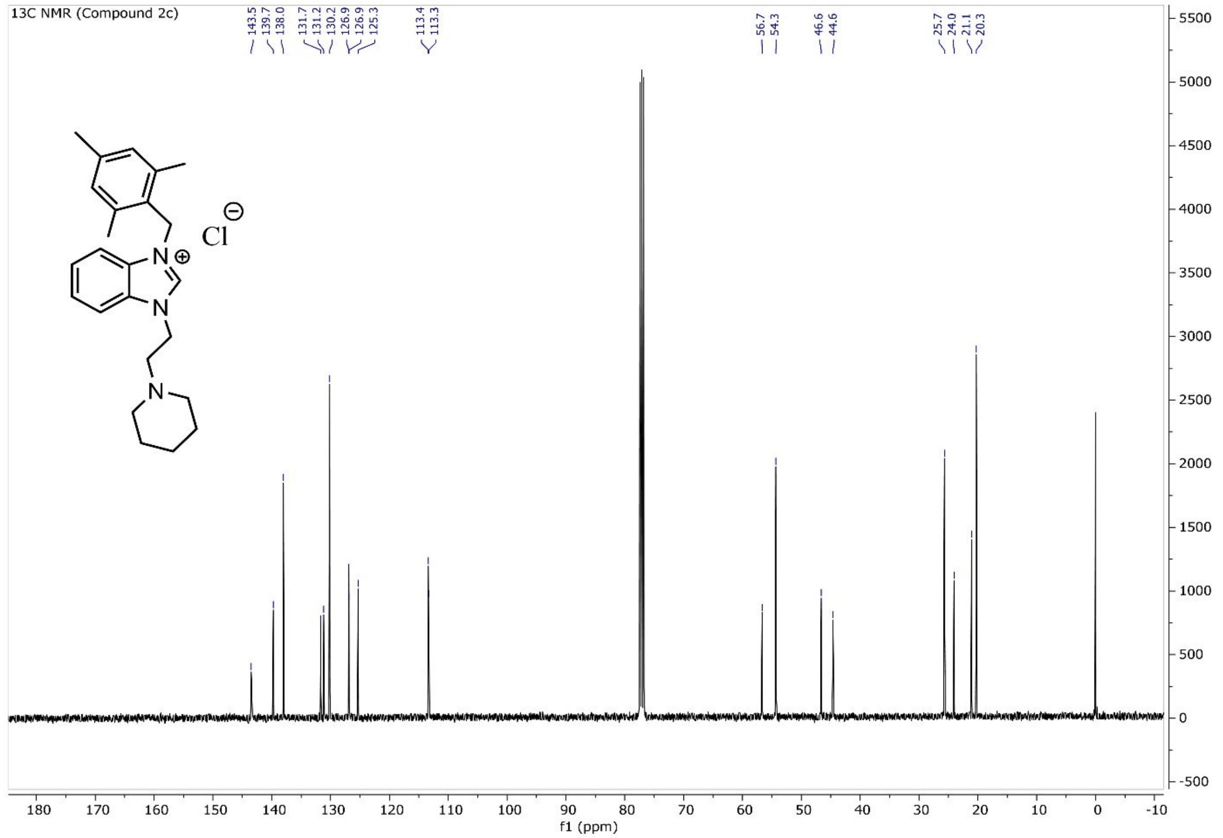

Figure S6. ^{13}C NMR spectrum of compound **2c** ($CDCl_3$)

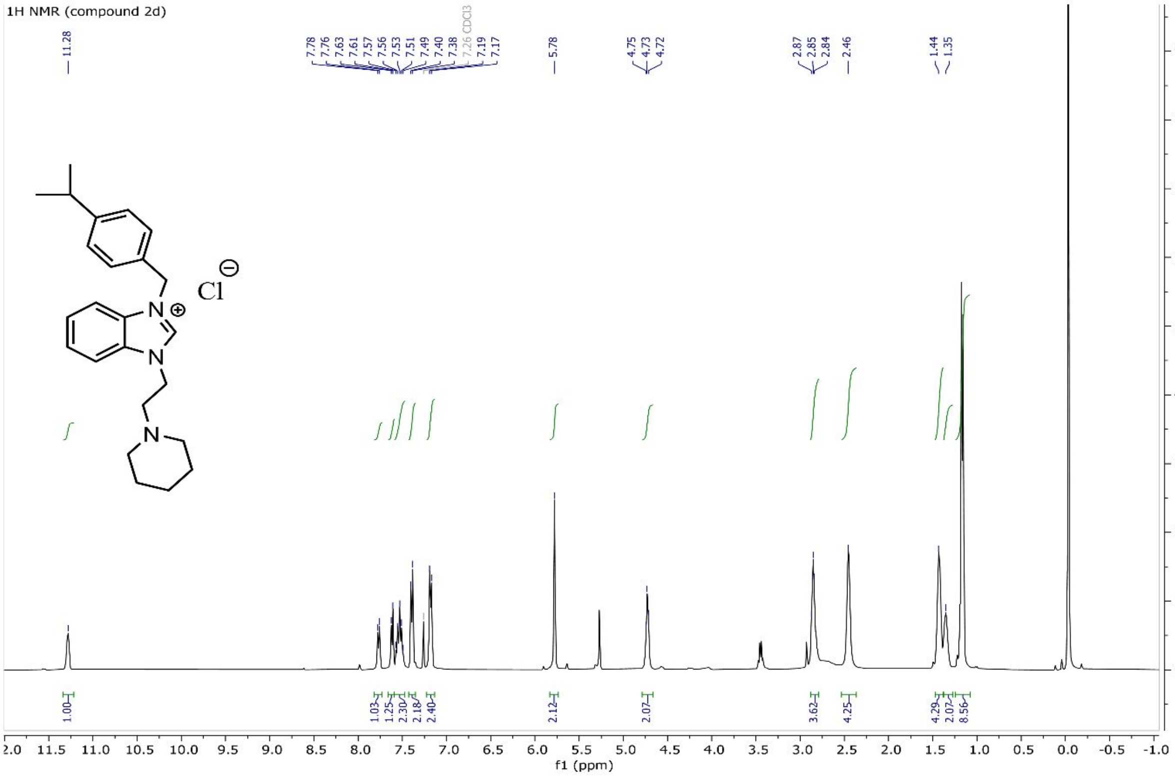

Figure S7. ^{1}H NMR spectrum of compound **2d** ($CDCl_3$)

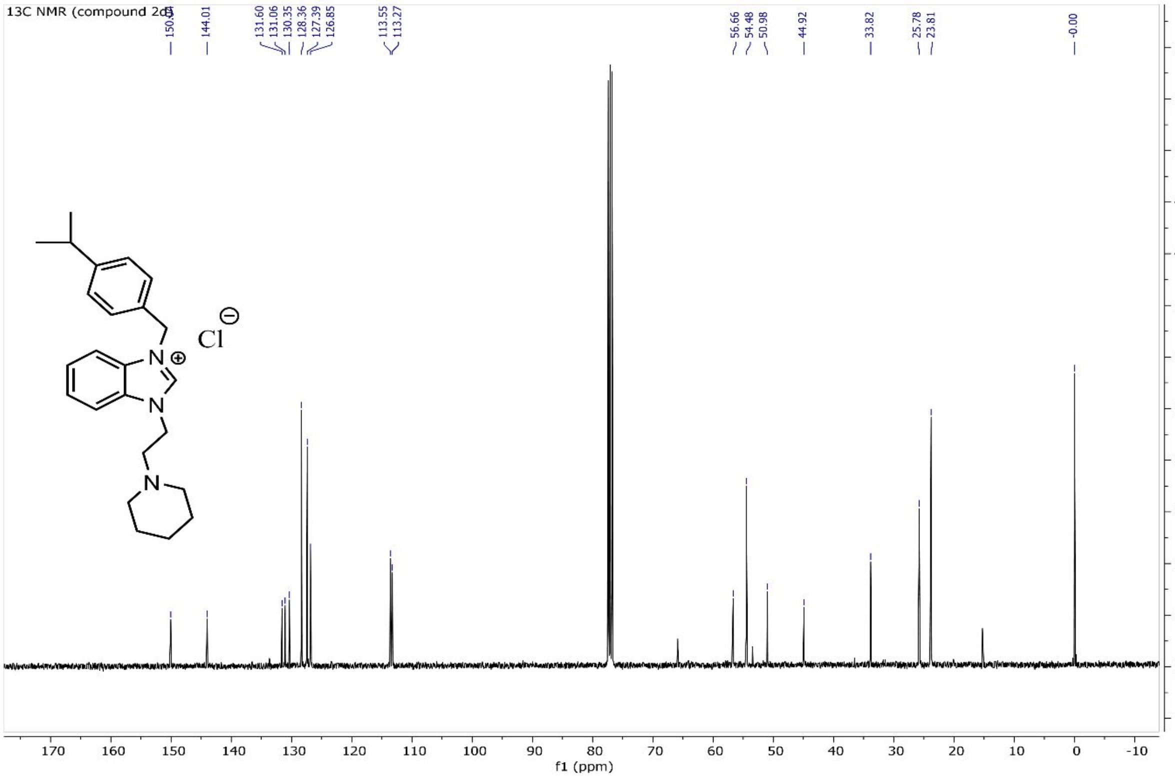

Figure S8. ^{13}C NMR spectrum of compound **2d** ($CDCl_3$)

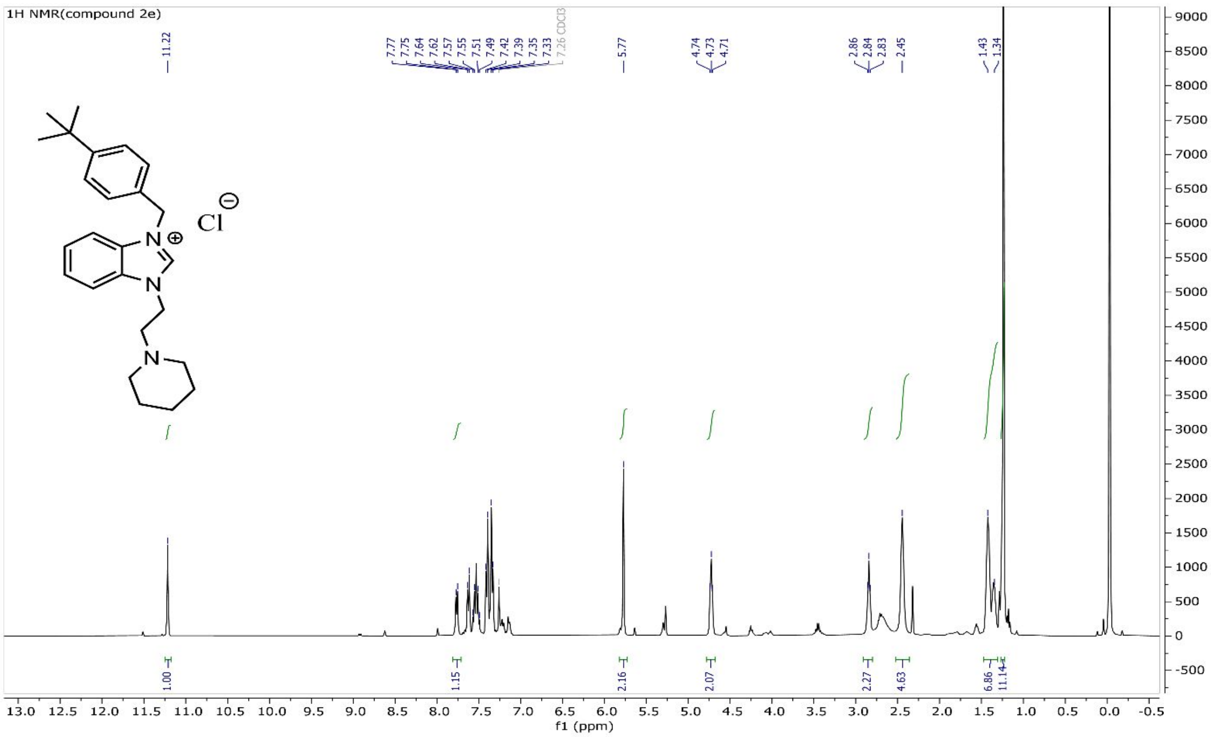

Figure S9. ^{1}H NMR spectrum of compound **2e** ($CDCl_3$)

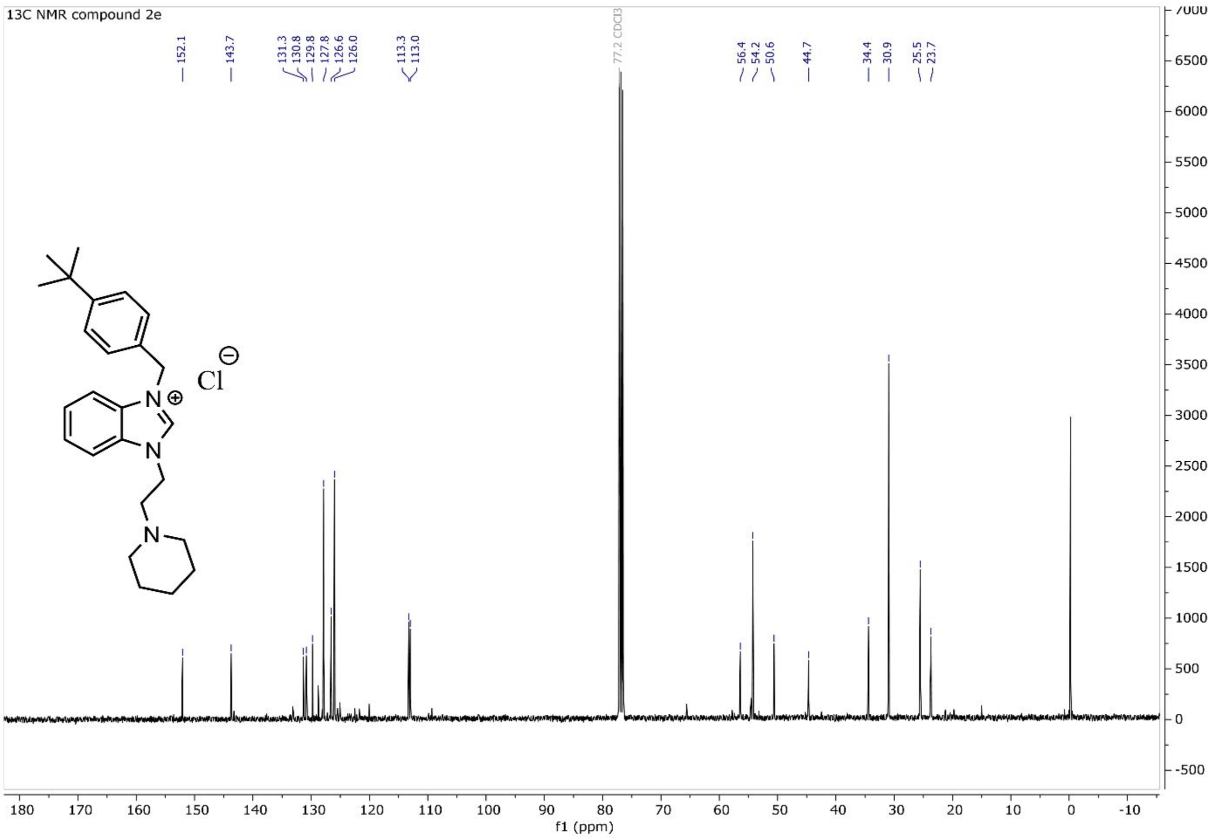

Figure S10. ^{13}C NMR spectrum of compound **2e** ($CDCl_3$)

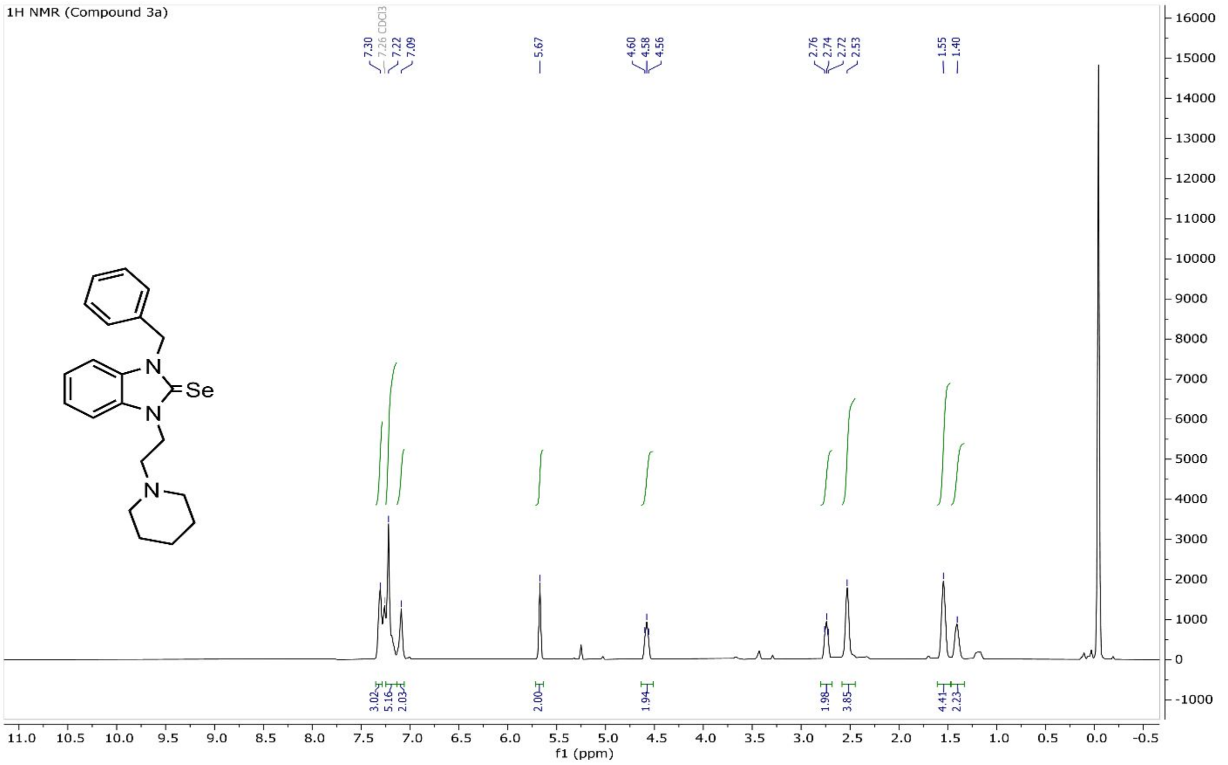

Figure S11. ^{1}H NMR spectrum of compound **3a** ($CDCl_3$)

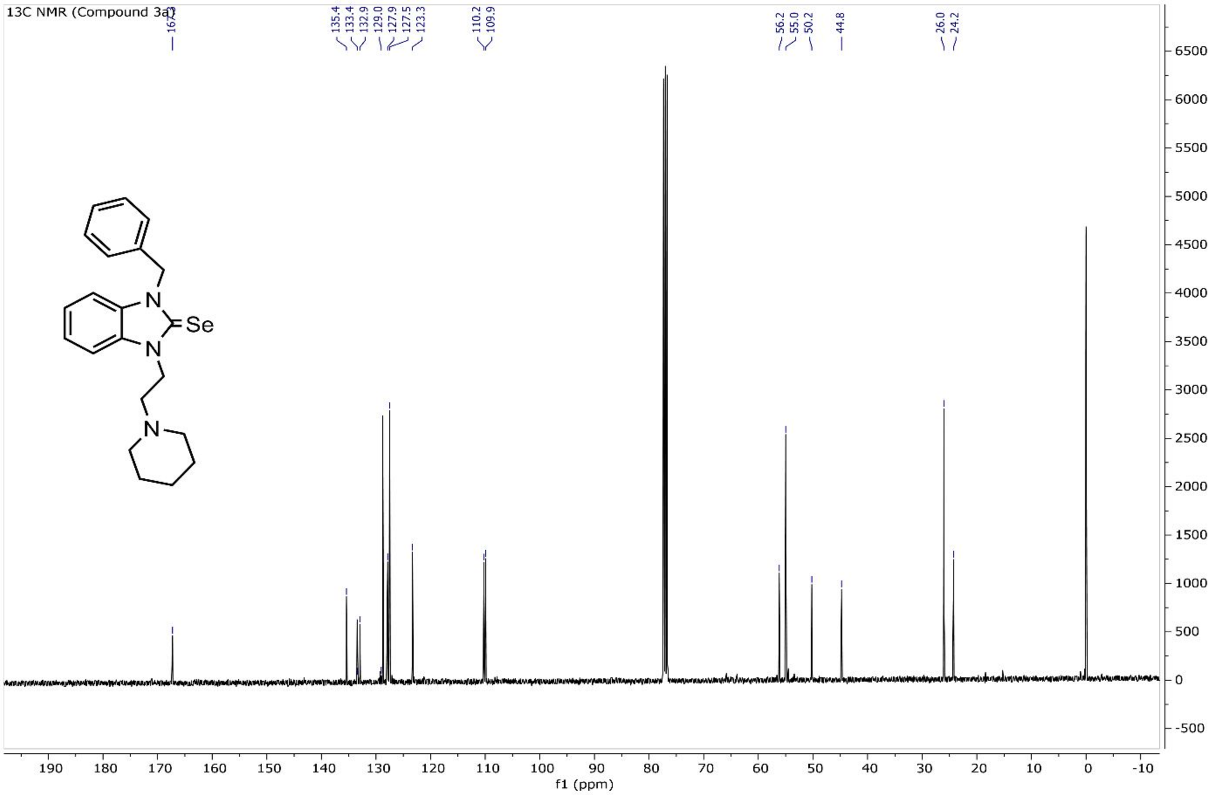

Figure S12. ^{13}C NMR spectrum of compound **3a** ($CDCl_3$)

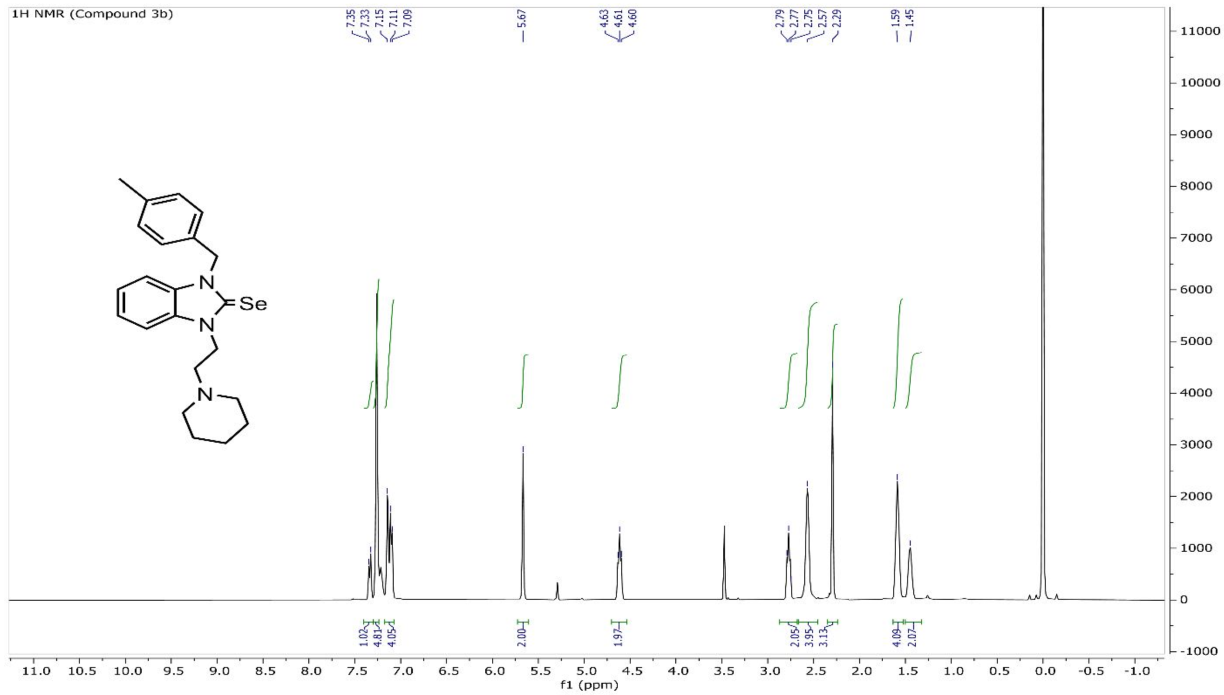

Figure S13. ^{1}H NMR spectrum of compound **3b** ($CDCl_3$)

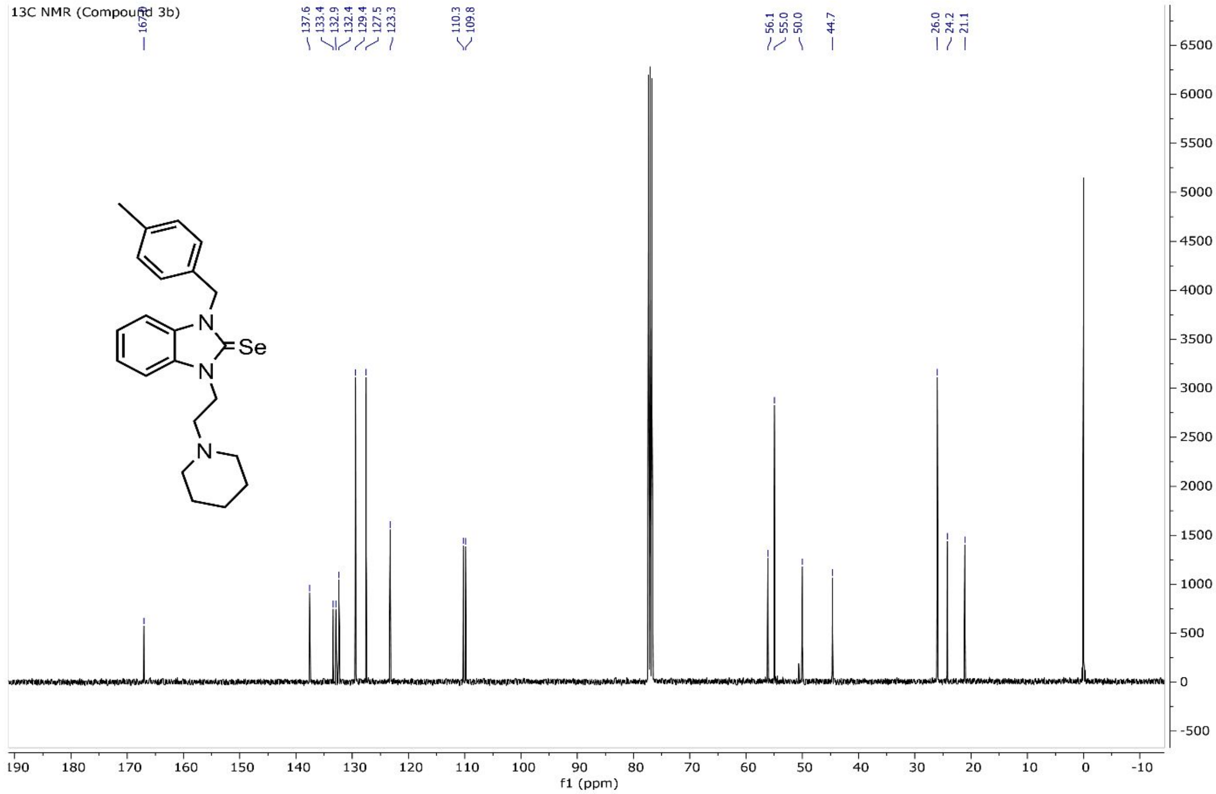

Figure S14. ^{13}C NMR spectrum of compound **3b** ($CDCl_3$)

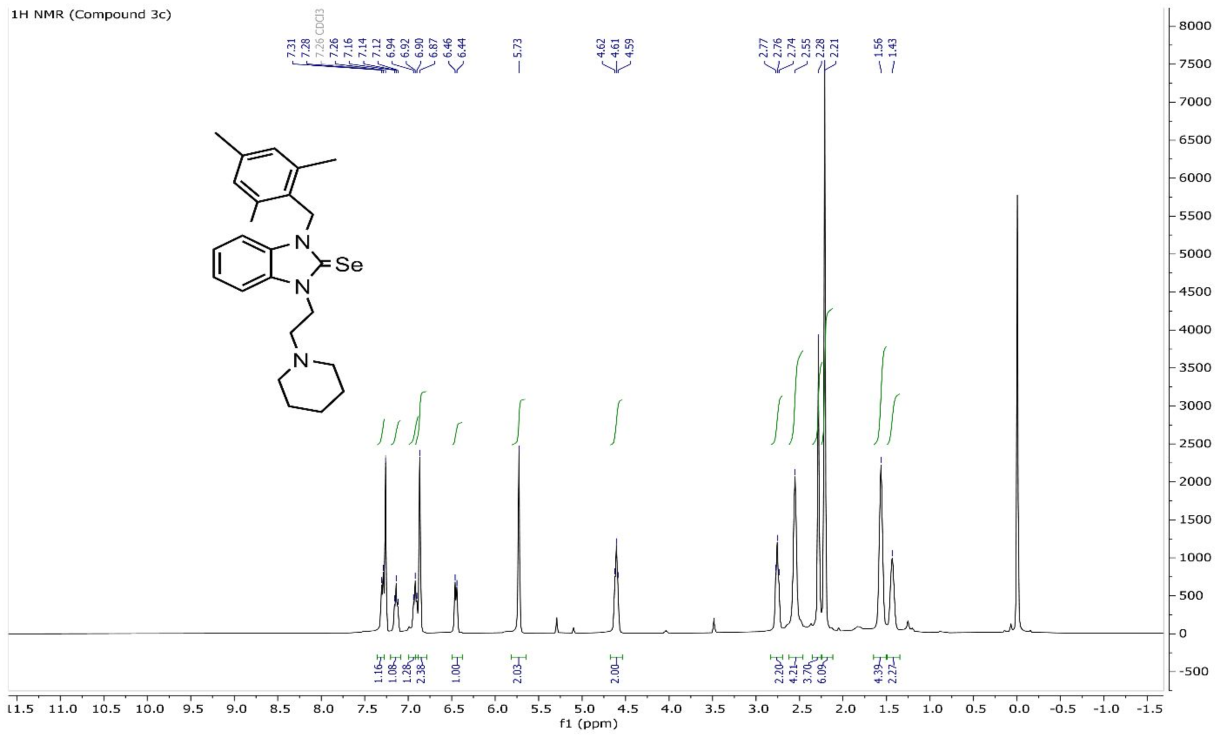

Figure S15. ^{1}H NMR spectrum of compound **3c** ($CDCl_3$)

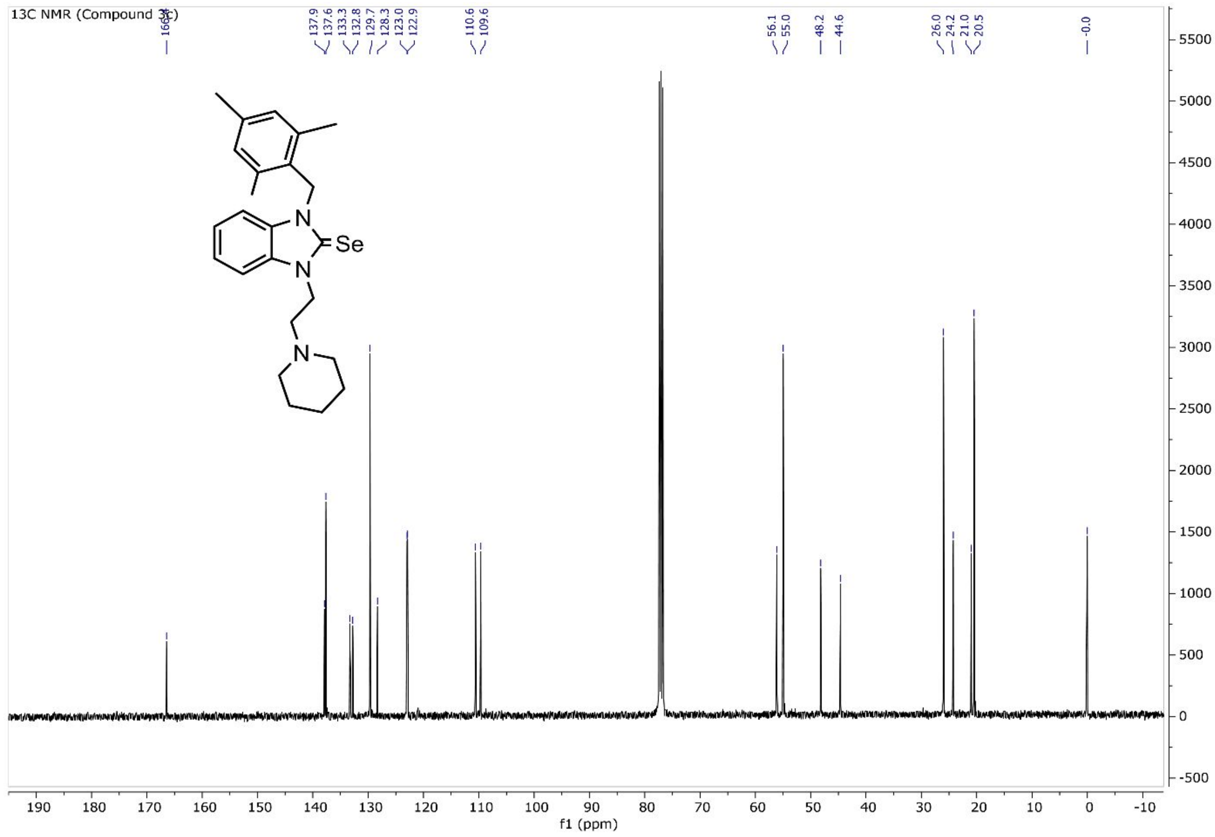

Figure S16. ^{13}C NMR spectrum of compound **3c** ($CDCl_3$)

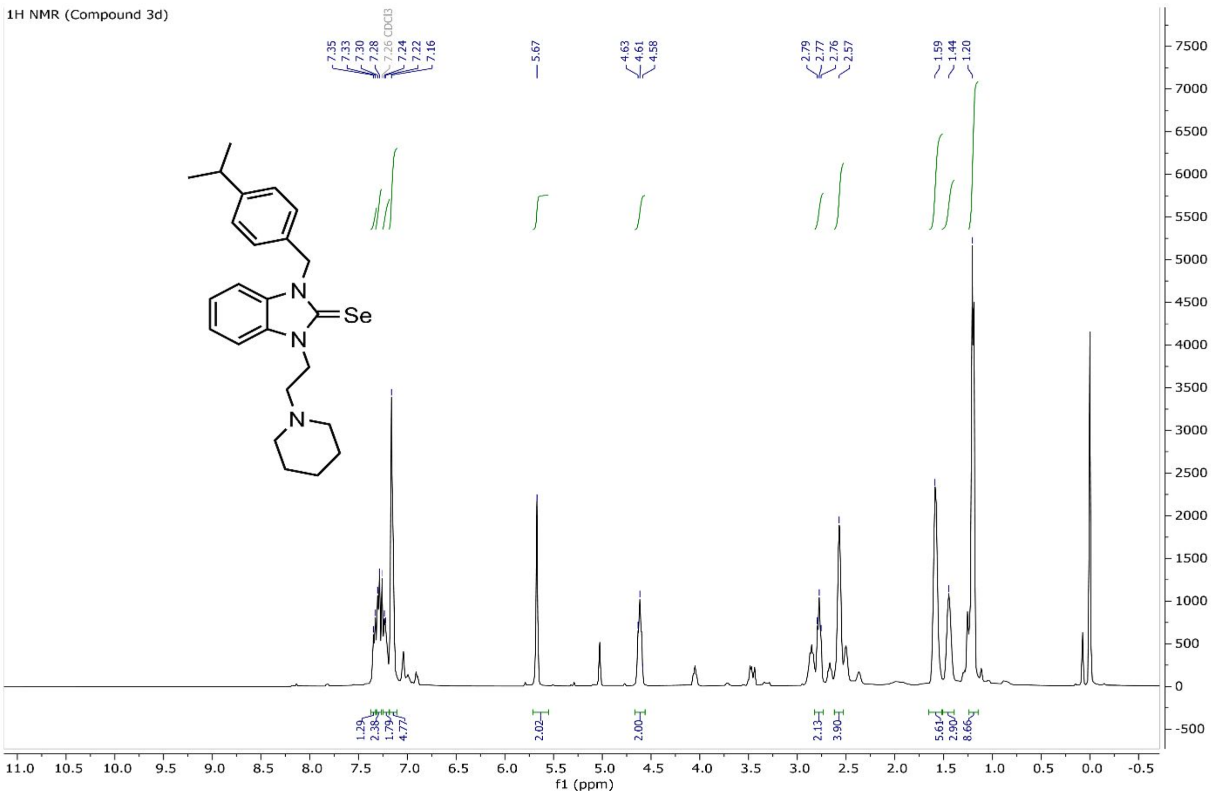

Figure S17. ^{1}H NMR spectrum of compound **3d** ($CDCl_3$)

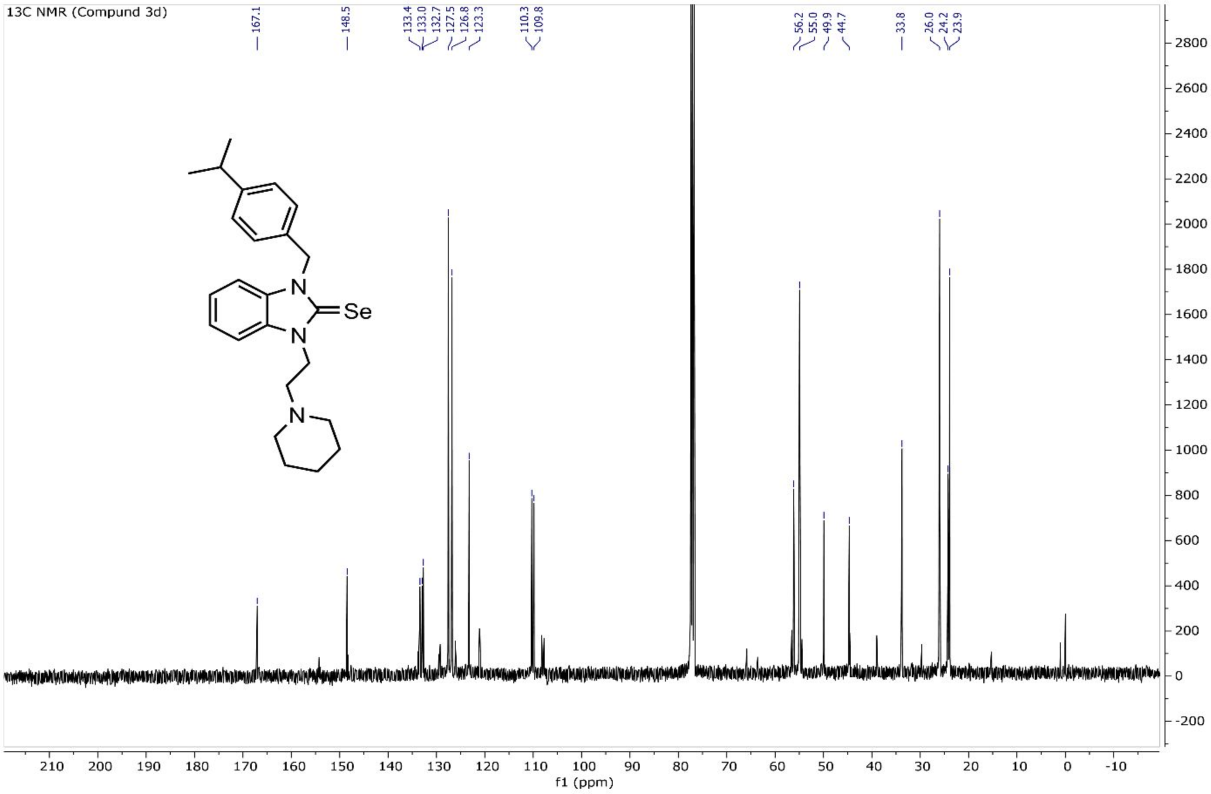

Figure S18. ^{13}C NMR spectrum of compound **3d** ($CDCl_3$)

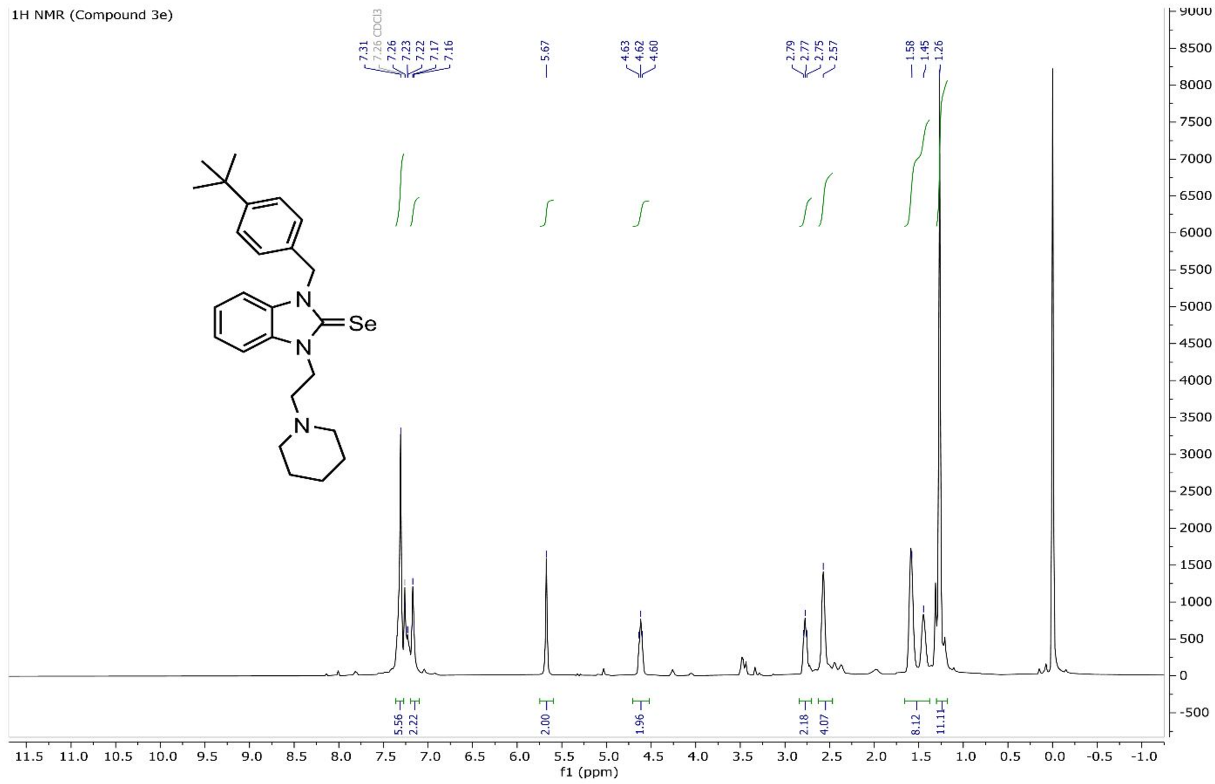

Figure S19. ^{1}H NMR spectrum of compound **3e** ($CDCl_3$)

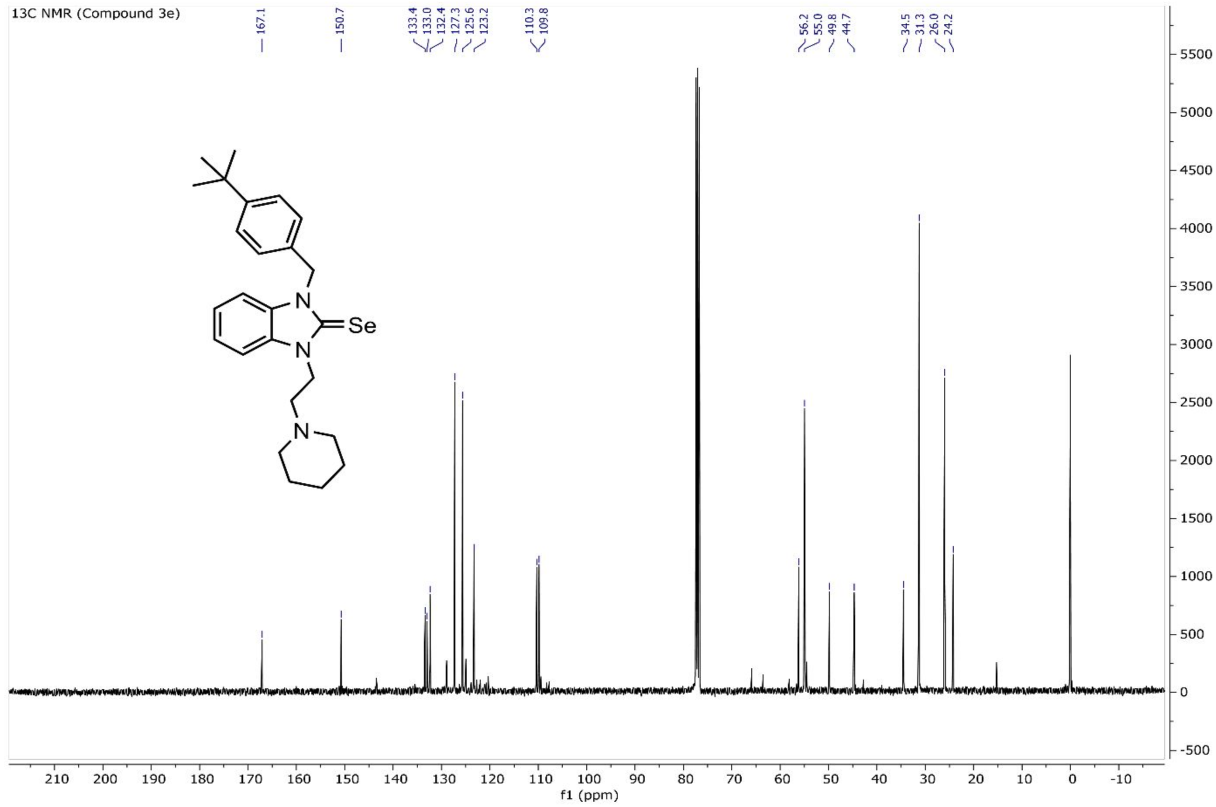

Figure S20. ^{13}C NMR spectrum of compound **3e** ($CDCl_3$)

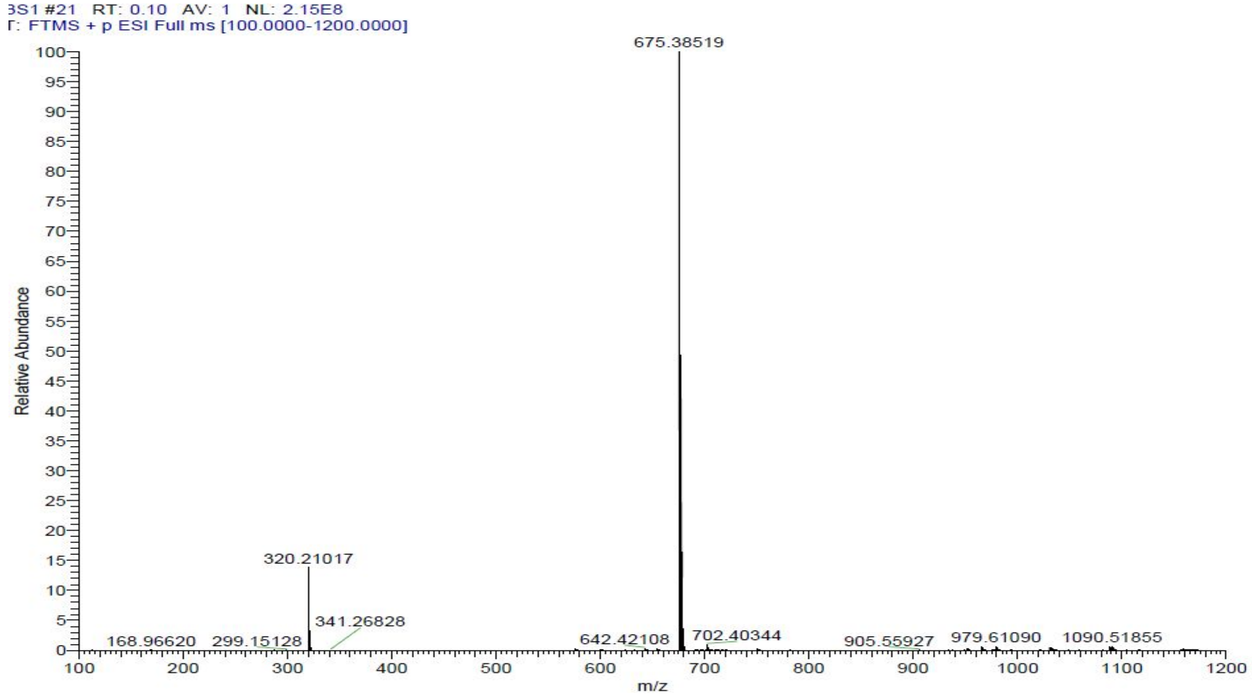

Figure S21. Mass spectrum of compound **2a**

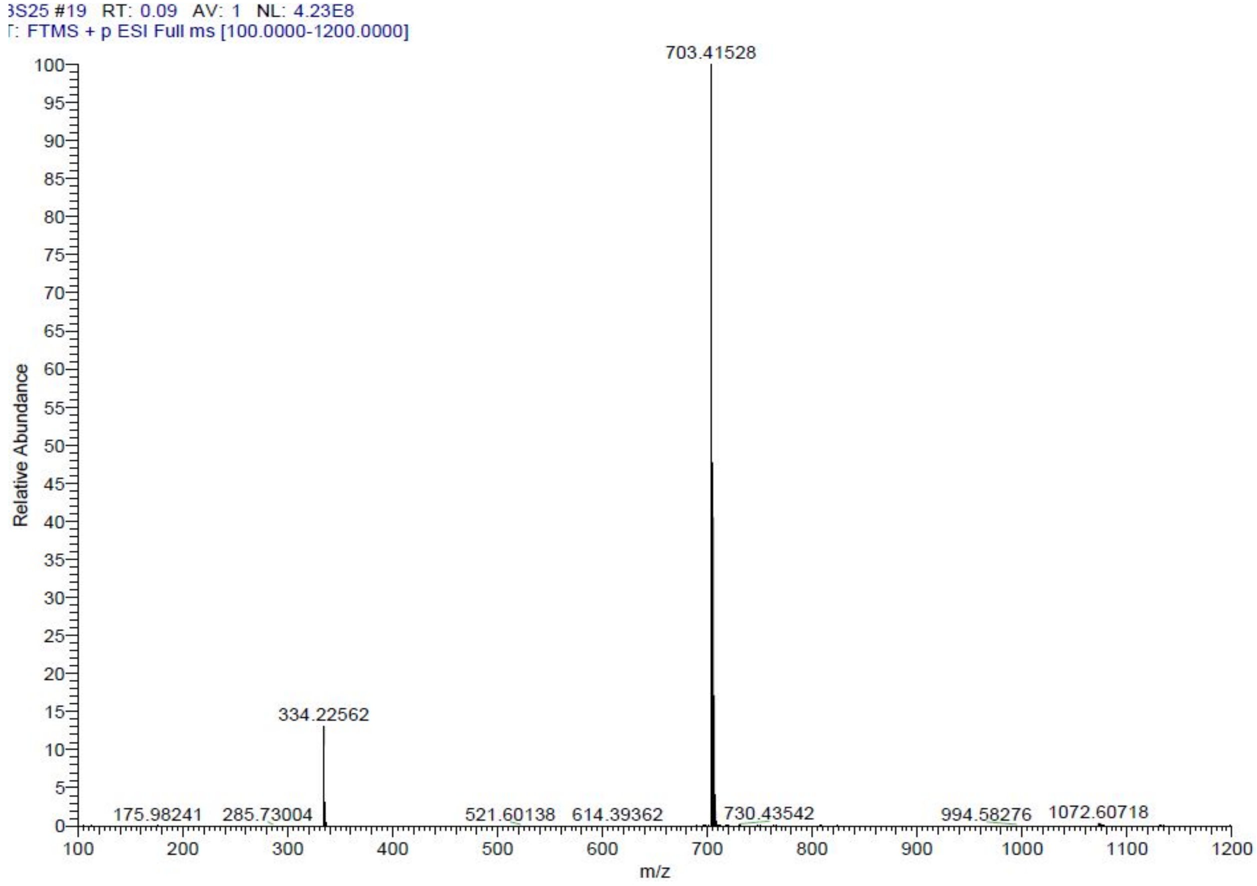

Figure S22. Mass spectrum of compound **2b**

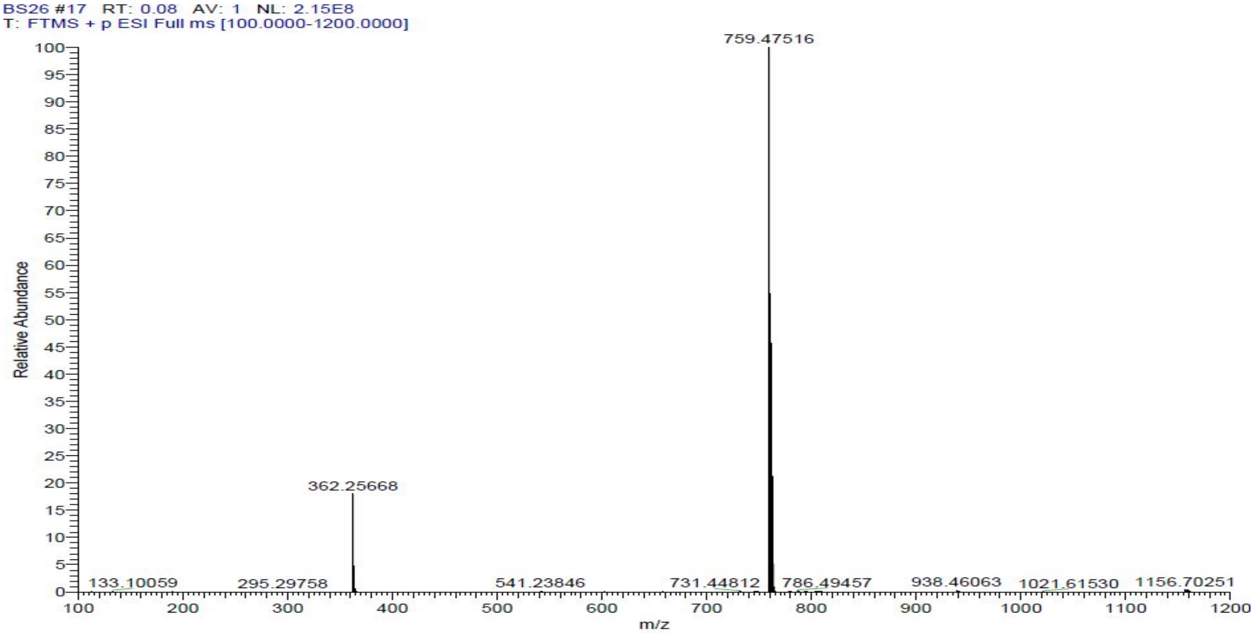

Figure S23. Mass spectrum of compound **2c**

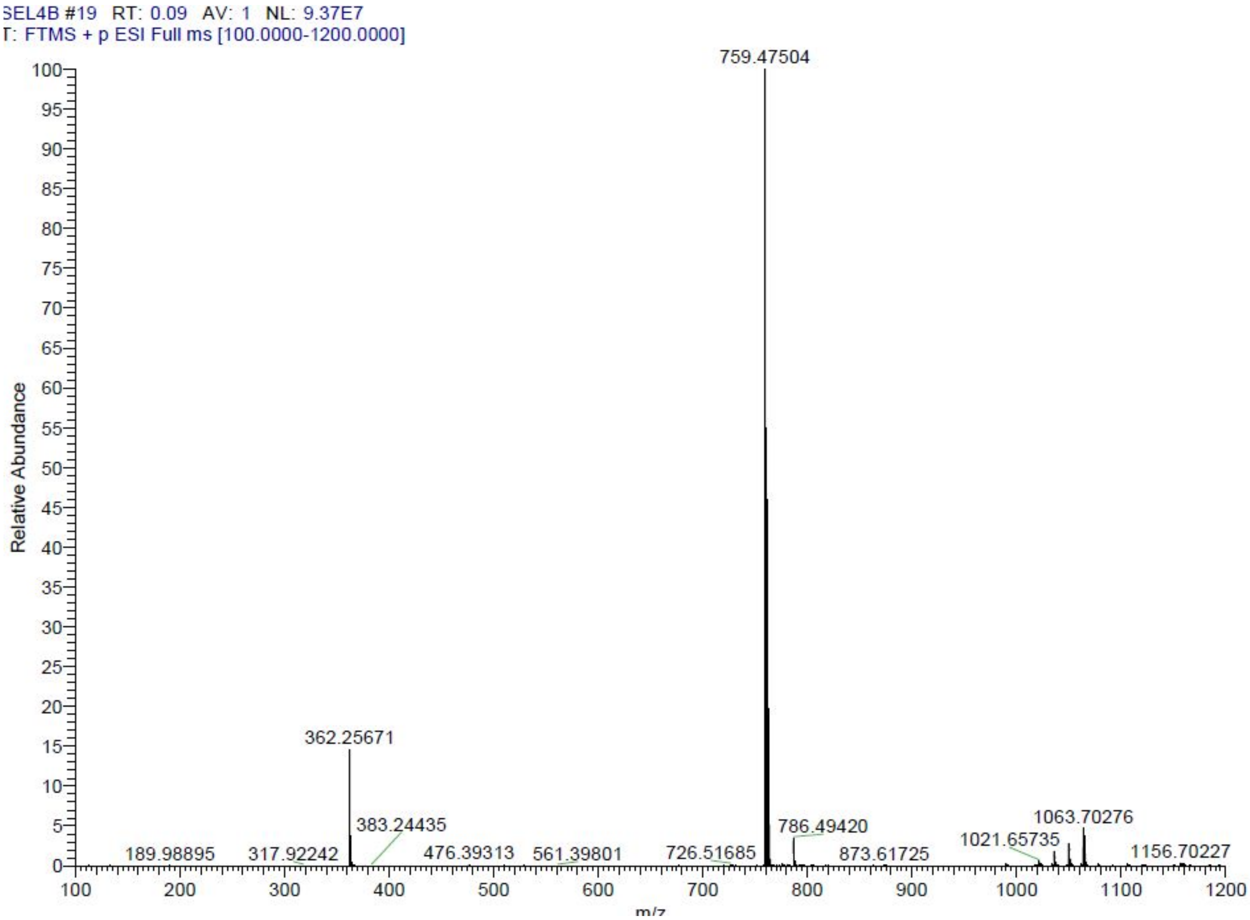

Figure S24. Mass spectrum of compound **2d**

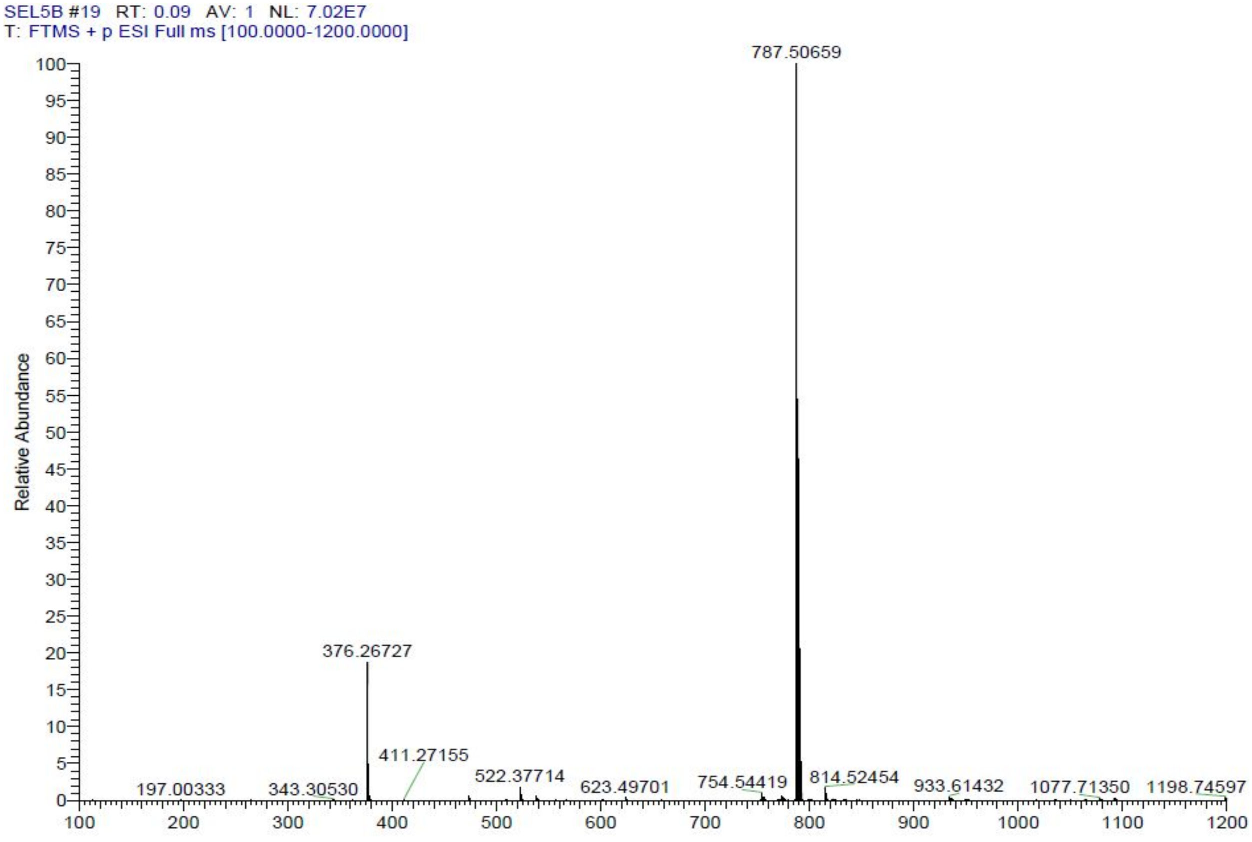

Figure S25. Mass spectrum of compound **2e**

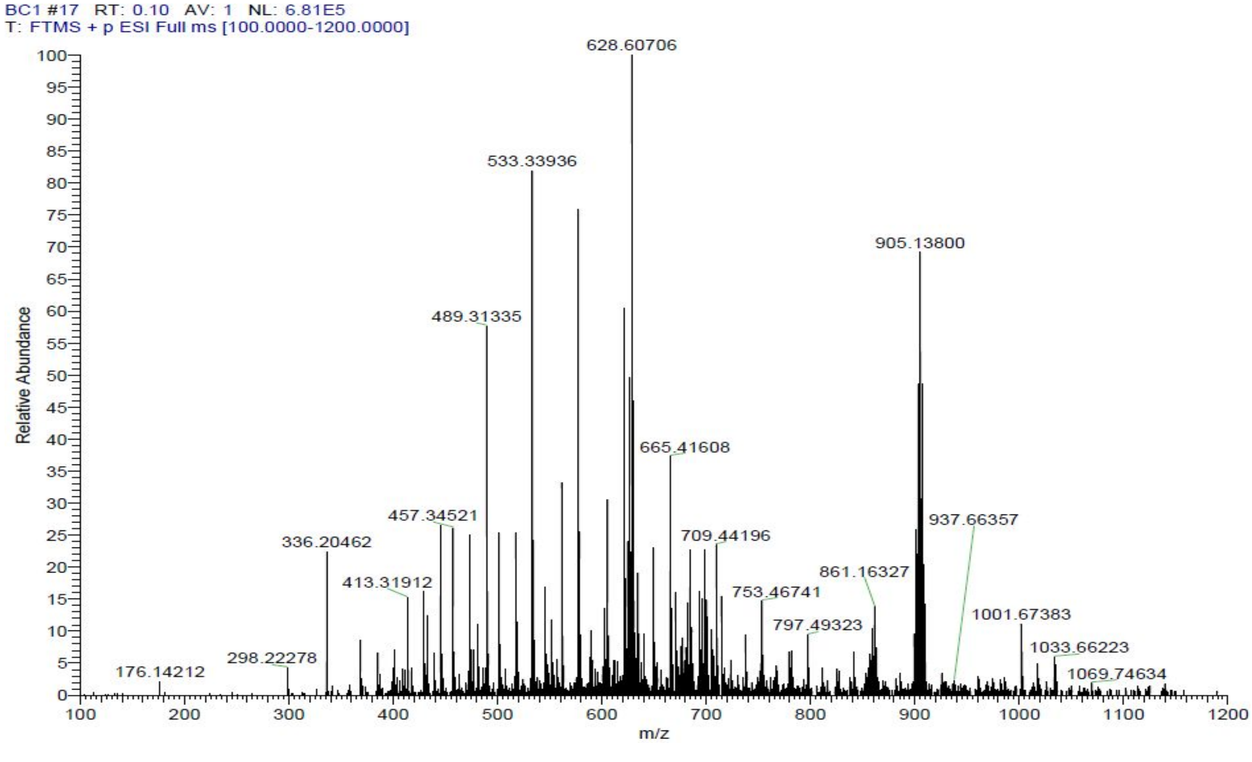

Figure S26. Mass spectrum of compound **3b**

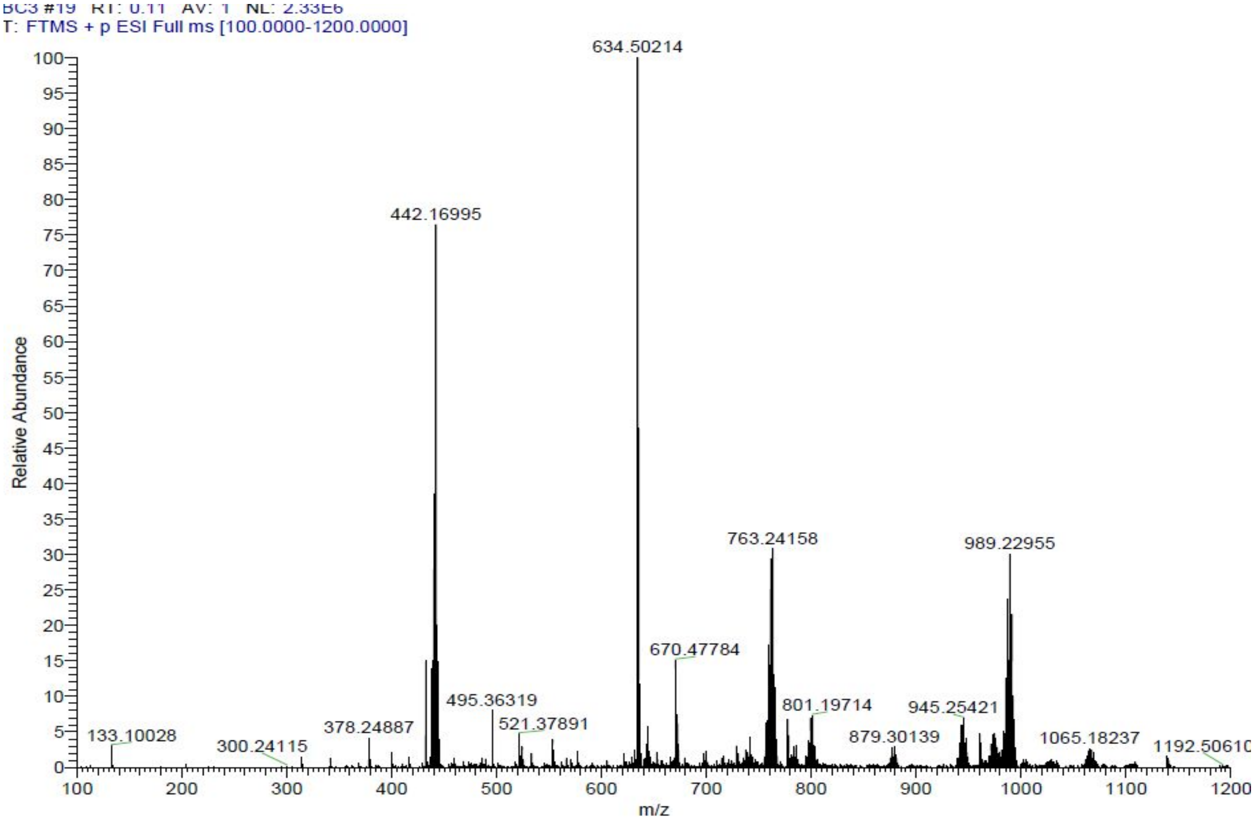

Figure S27. Mass spectrum of compound **3c**

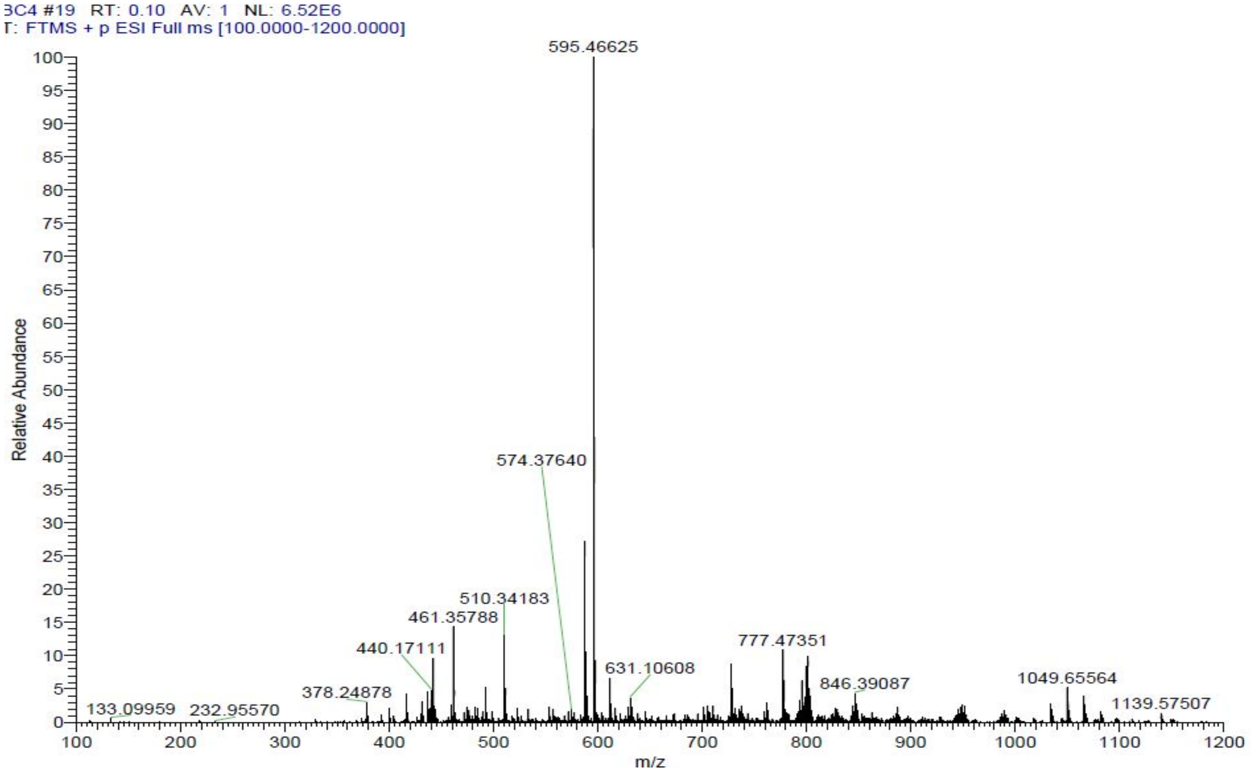

Figure S28. Mass spectrum of compound **3d**

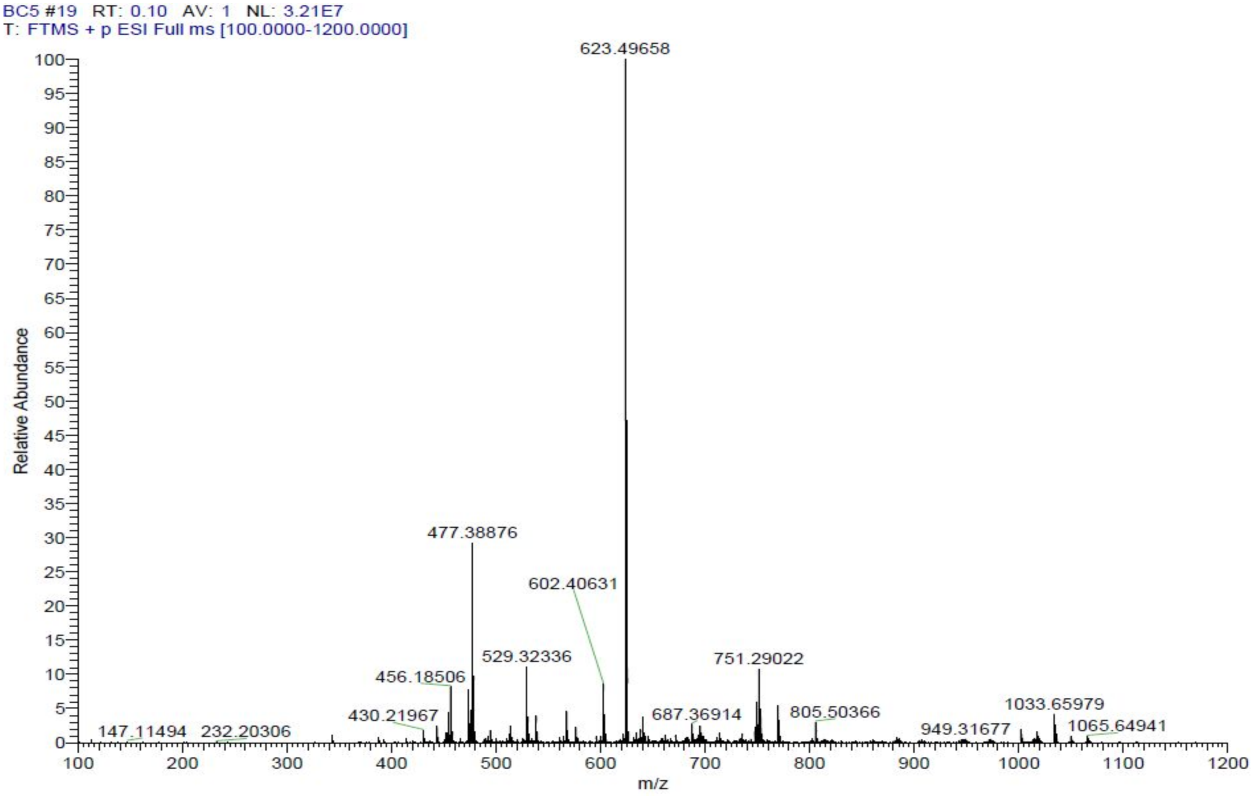

Figure S29. Mass spectrum of compound **3e**

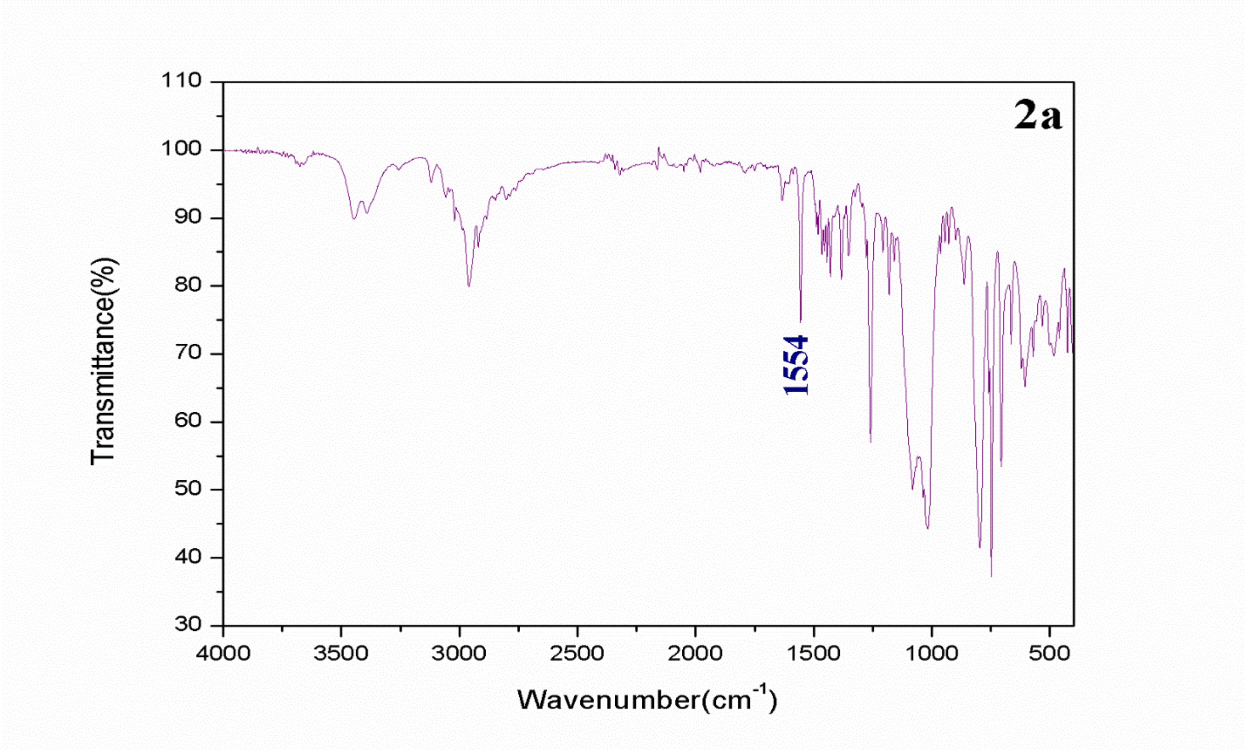

Figure S30. IR spectrum of compound **2a**

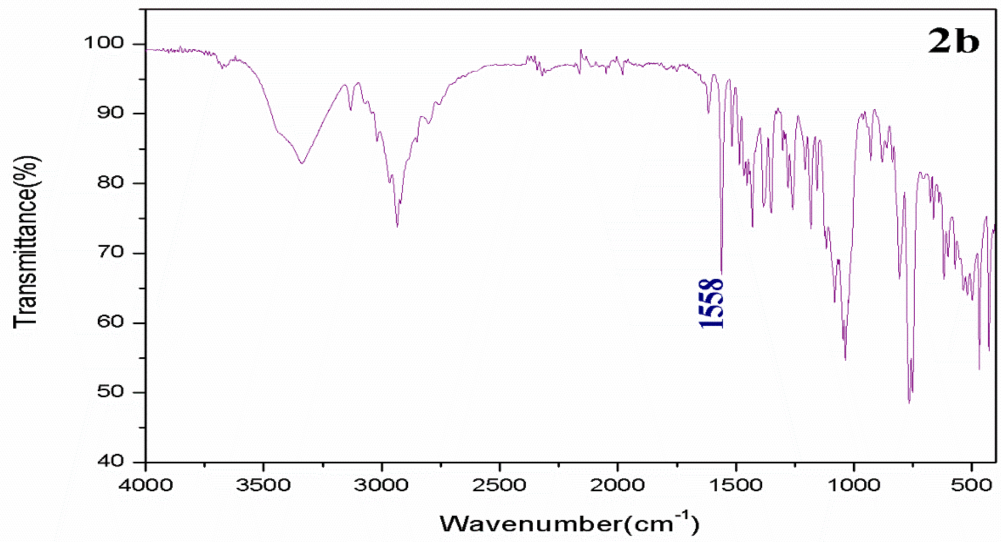

Figure S31. IR spectrum of compound **2b**

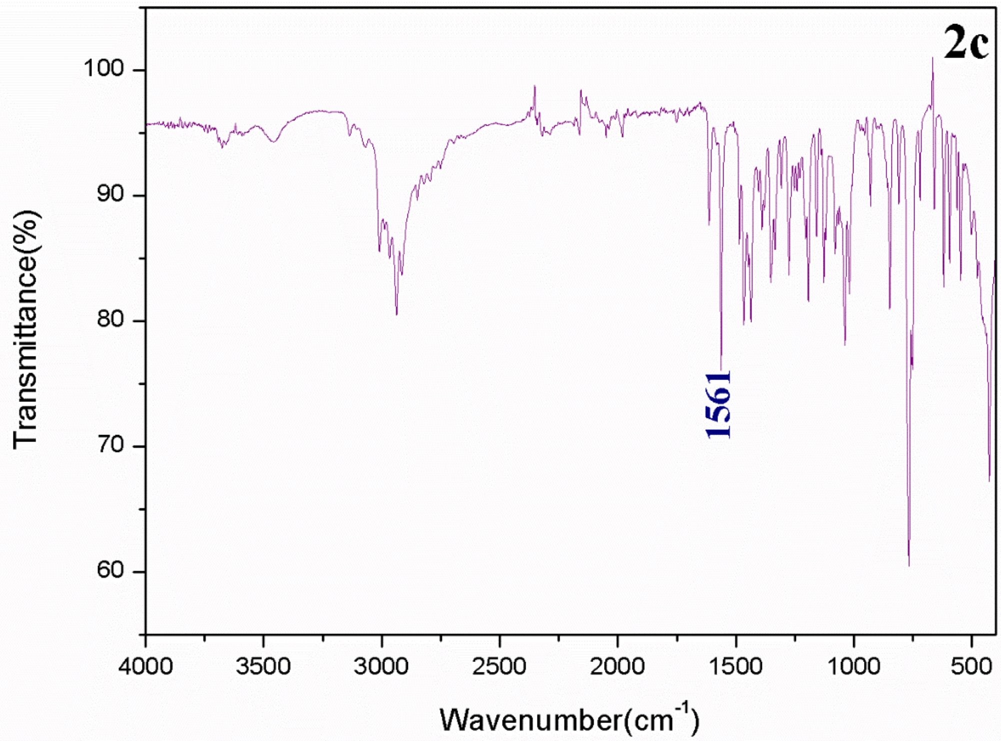

Figure S32. IR spectrum of compound **2c**

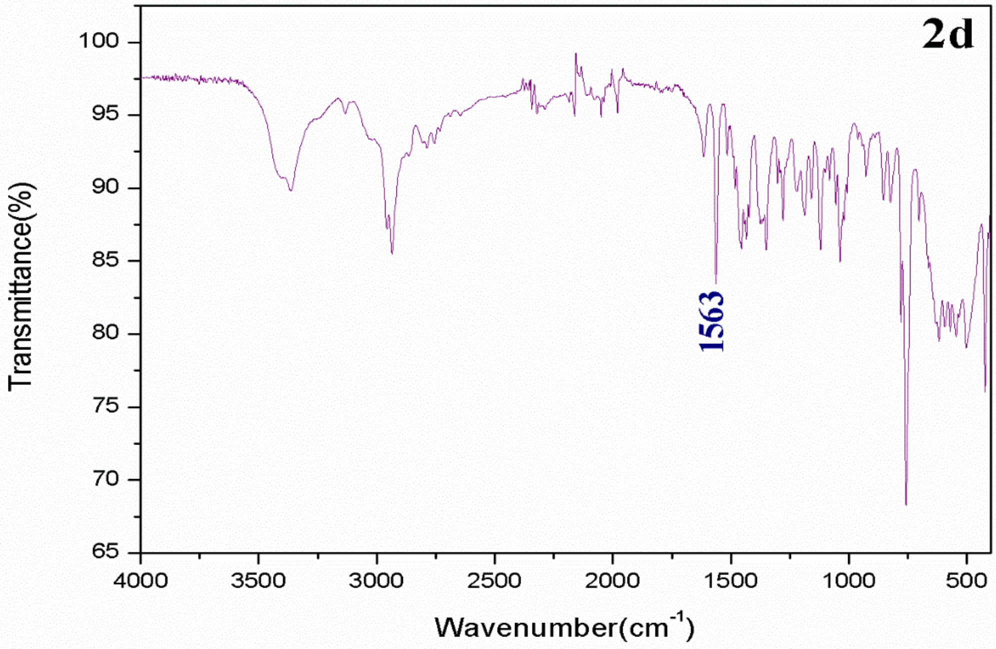

Figure S33. IR spectrum of compound **2e**

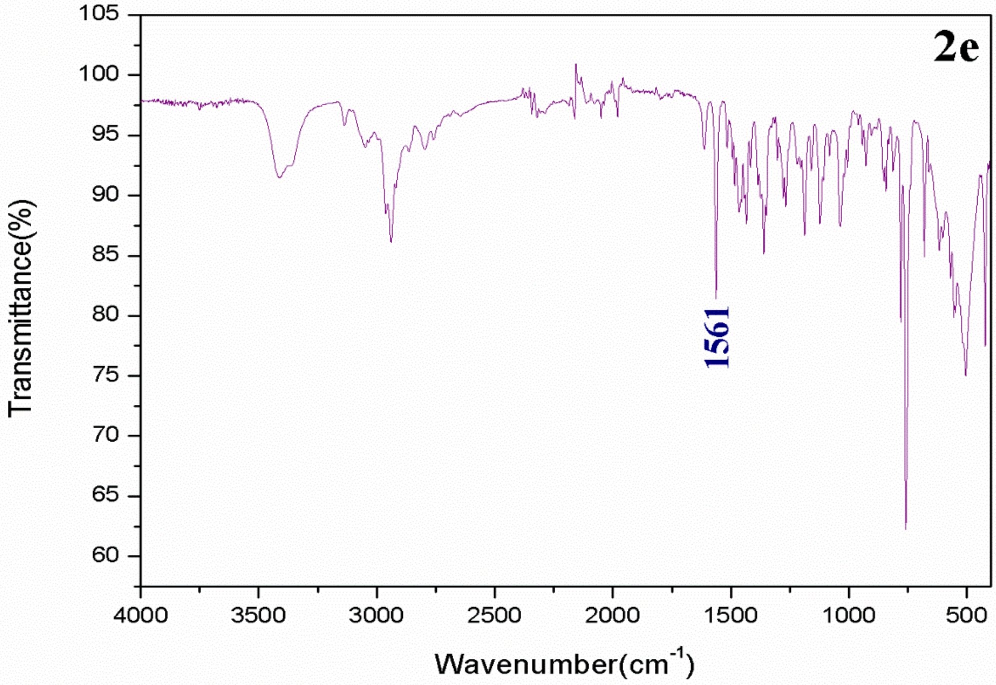

Figure S34. IR spectrum of compound **2e**

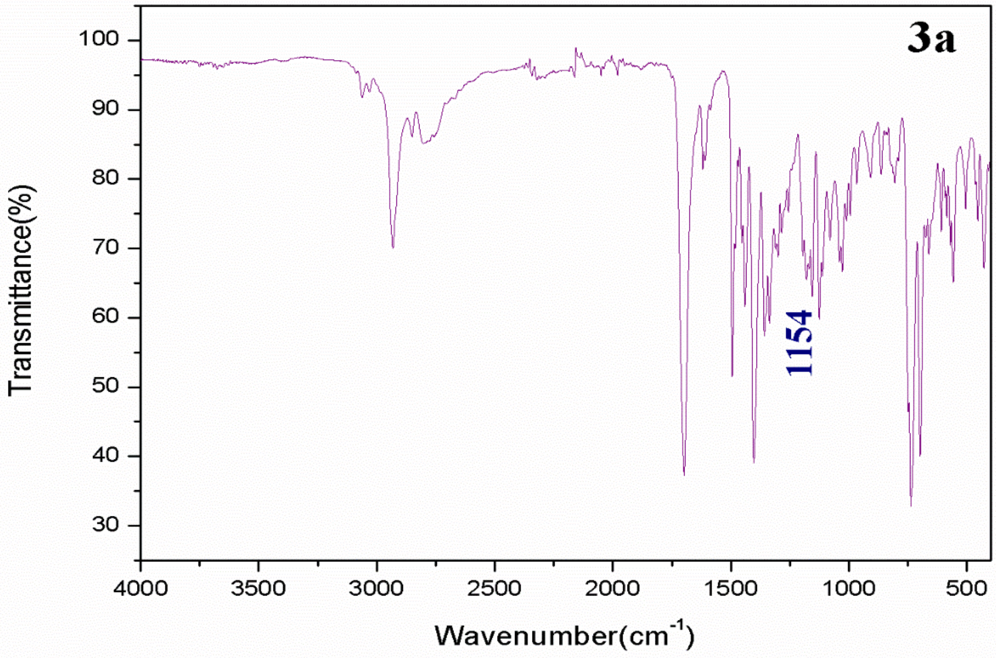

Figure S35. IR spectrum of compound **3a**

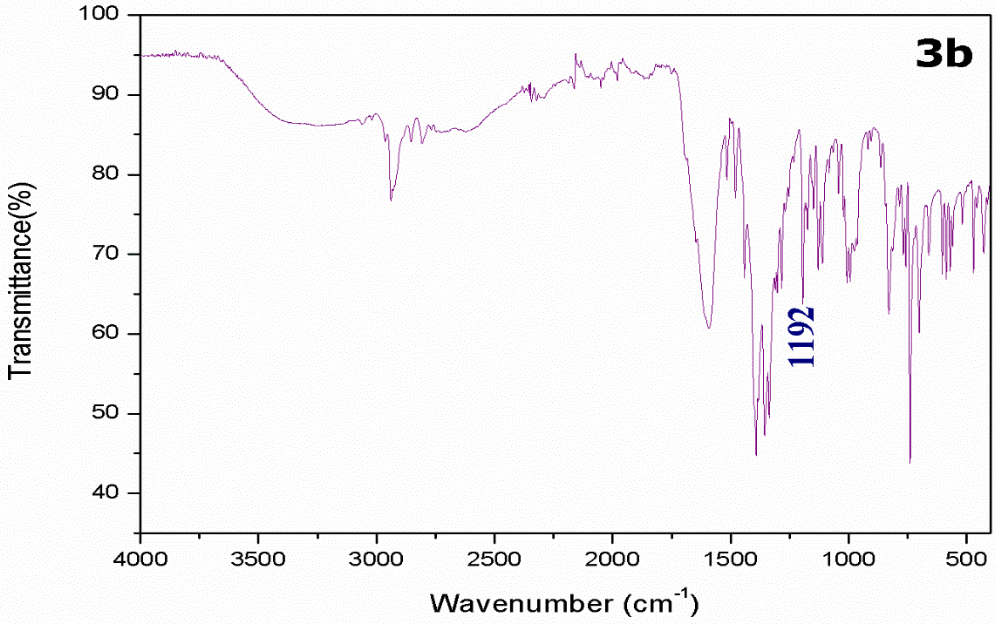

Figure S36. IR spectrum of compound **3b**

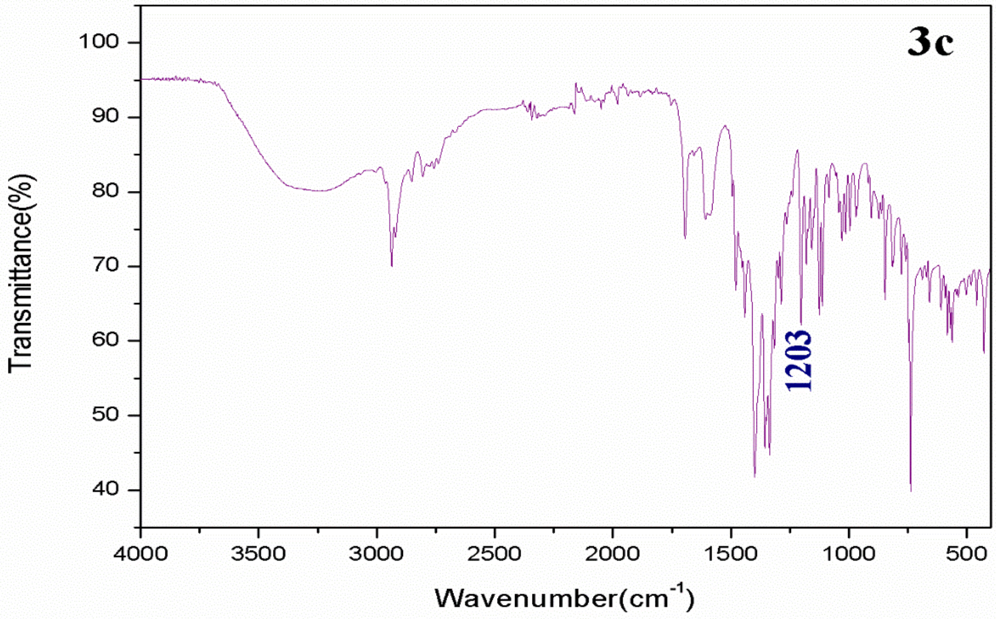

Figure S37. IR spectrum of compound **3c**

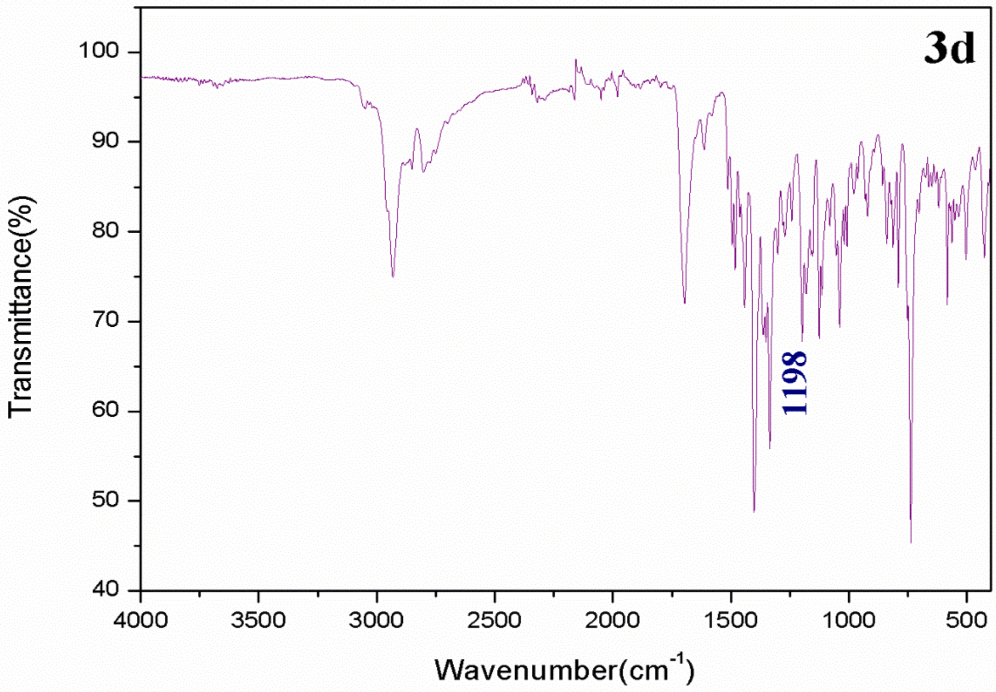

Figure S38. IR spectrum of compound **3d**

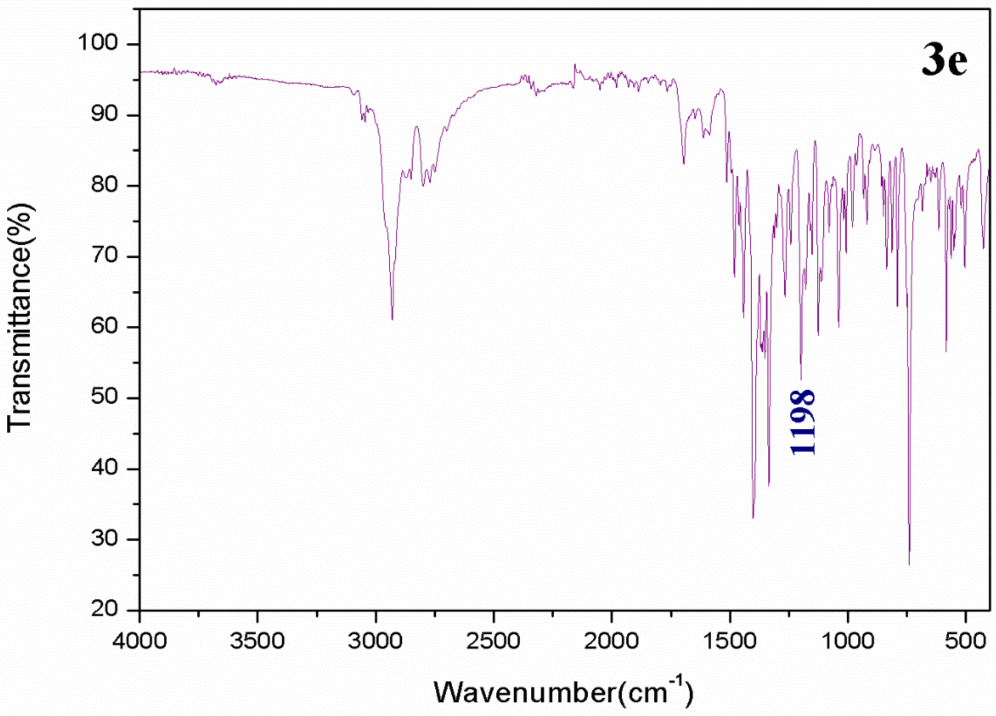

Figure S39. IR spectrum of compound **3e**
